# Illumina-based Analysis of Endophytic Bacterial Diversity of four *Allium* species

**DOI:** 10.1038/s41598-019-51707-7

**Published:** 2019-10-24

**Authors:** Yonghong Huang

**Affiliations:** College of Horticulture, Qingdao Agricultural University, Qingdao, 266109 China

**Keywords:** Symbiosis, Rhizobial symbiosis

## Abstract

*Allium* species are popular vegetables in China and possess antifungal and antibacterial activities. This study aimed to compare the endophytic bacterial community in the four crucial *Allium* species in China, Chinese leek (CL), garlic (GA), onion (ON,) and Welsh onion (WO), using sequences of the V3–V4 region of the bacterial 16S rRNA gene. A total of 1,036,637 high-quality sequences and 719 operational taxonomic units (OTUs) were obtained across all libraries. A total of 20 phyla, 50 classes, 80 orders, 134 families, and 234 genera were identified. Among them, 18 OTUs and 19 genera were shared among the four *Allium* species. *Proteobacteria* (42.68%) and *Bacteroidetes* (20.18%) were the dominant phyla in CL, while one unclassified (>70%) was the dominant phyla in the other three *Allium* species. The alpha-diversity analysis showed the bacterial richness and diversity in CL were significantly higher than those in the other three *Allium* species. Principal coordinate analysis (PCA) showed endophytic bacterial communities in GA, WO, and ON were more similar than those in CL. Phylogenetic Investigation of Communities by Reconstruction of Unobserved States (PICRUSt) analysis revealed endophytic bacteria mostly enriched in Membrane Transport, Amino Acid Metabolism and Carbohydrate Metabolism pathway. 17 of the 23 Kyoto Encyclopedia of Genes and Genomes (KEGG) categories and 159 of the 206 lower-level KEGG pathways in CL were significantly higher than those in the other three *Allium* species. Pearson’s correlation indicated that KEGG pathways with significant differences among the *Allium* species were closely related to the bacterial genera with significant differences between the *Allium* species. The findings of our study provided insight into the complex endophytic microbial communities in *Allium* species.

## Introduction

Healthy plant tissues contain endophytic bacteria that do not cause pathogenic reactions. In recent decades, studies have shown that these diverse and active microbial communities are not merely “passengers” within plants, but instead play an essential role in plant growth, development, and resistance to biotic and abiotic stresses^1^. Previous studies have shown that endophytic bacteria enhance the growth and development of host plants^2,3^, improve the efficiency of phytoremediation in heavy metal-degraded soils^4–7^, inhibit the growth of plant pathogens and effectively reduce the incidence of plant disease^8–10^, and increase tolerance to salinity stress^11,12^. Therefore, isolating, identifying, and applying endophytic bacteria have become of interest in research to identify safer biological additives.

*Allium* species are used worldwide as spices, vegetables, and medicinal plants. Traditionally, they are essential in the daily diet of people in many Asian countries and are used to flavor foods^13^. They are also an abundant source of endophytic microorganisms. An endophytic bacterium *Sphingomonas* sp. strain HJY isolated from the leaves of Chinese leek (*Allium tuberosum*) showed the potential ability to degrade *Chlorpyrifos*^14^. The fungal endophyte *Clonostachys rosea* ICIPE 707 isolated from healthy onion (*Allium cepa*) colonized onion plants and significantly repressed the feeding punctures, oviposition, and numbers of thrips on plants^15^. *Streptomyces* sp.TP-A0569 isolated from the stem of the Welsh onion (*Allium fistulosum*) and *Streptomyces* sp.TP-A0595 isolated from Chinese leek produced fistupyrone and 6-prenylindole, respectively, both of which significantly inhibited the infection of Chinese cabbage by *Alternaria brassicicola*^16,17^. The endophytic fungus strain *Trichoderma brevicompactum* 0248 isolated from garlic (*Allium sativum*) has a marked inhibitory activity on *Rhizoctonia solani* and *Botrytis cinerea*^18^.

Garlic (GA), onion (ON), Welsh onion (WO), and Chinese leek (CL) are typical *Allium* species that are popular vegetables in China. To some extent, they have similar physicochemical properties. For example, they all are perennial herbs with various kinds of bulbs and used as a flavoring for daily food. They all contain the well-studied sulfur compounds, saponins, flavonoids, nitrogen compounds, and peptides that make *Allium* species effective against pathogenic microbes^19^. But so far, very little is known about their endophytes. What is the composition of the endophyte community of the four *Allium* species? What is the relationship between them? In this study, we comparatively analyzed the endophytic bacterial communities in these four species using Illumina sequencing, to provide valuable information for isolating and utilizing endophytic bacteria with beneficial properties from *Allium* species.

## Results

### Sequence characteristics of the four *Allium* species

**Figure 1:**
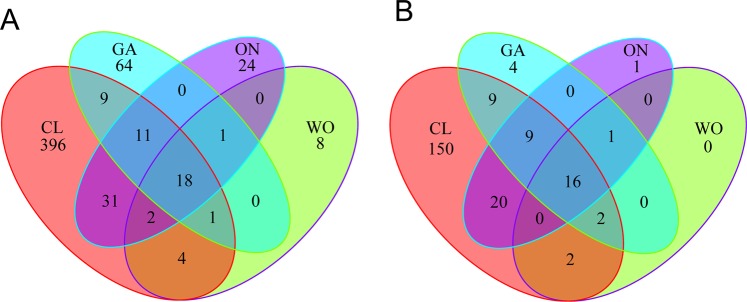
Venn diagrams of the endophytic bacteria of the four *Allium* species. The OTUs (**A**) and bacterial genera (**B**) shared between *Allium* species. CL: Chinese leek; WO: Welsh onion; GA: Garlic; ON: Onion.

After processing, a total of 1,036,637 filtered sequences were obtained from the four *Allium* species, the numbers of which ranged from 61,541 to 101,135 in these samples (Table 1). Rarefaction curves (Supplementary Fig. S1), together with the estimated coverage values (Table 2), suggested that the libraries were sufficiently large to capture most of the bacterial diversity in the samples. A total of 719 operational taxonomic units (OTUs) were obtained across all libraries at 97% identity. A total of 18 OTUs, accounting for 29.57% of the total abundance, were shared among all samples. There were 396, 64, 24, and 8 OTUs exclusive to CL, GA, ON, and WO, respectively (Fig. 1A). Besides the shared OTUs, 16 bacterial genera were common to all samples. There were 150, 4, 1, and 0 bacterial genus exclusive to CL, GA, ON, and WO, respectively (Fig. 1B).

**Table 1:** Statistics of sequences from the four *Allium* species.

Sample	Clean sequences	Filter sequences	Percent
CL1	104537	101,135	96.74%
CL2	101538	97,814	96.33%
CL3	75316	73,726	97.88%
WO1	80666	78,952	97.87%
WO2	63260	61,541	97.28%
WO3	103209	99,372	96.28%
GA1	101393	98,498	97.14%
GA2	90773	89,212	98.28%
GA3	84732	83,482	98.52%
ON1	90120	87,924	97.56%
ON2	83307	81,838	98.23%
ON3	84192	83,143	98.75%
Total	1,063,043	1,036,637	98.75%

Note: Clean sequences: the numbers of combinated sequences; Filter sequences: the numbers of sequences after filtration.

**Table 2:** Microbial community richness and diversity indices of the four *Allium* species at a 97% similarity threshold.

Sample	Nseqs	OTUs	Coverage	Chao	Ace	Shannon	Npshannon
CL	5121.33 ± 1503.40a	435.33 ± 55.19a	0.98 ± 0.01a	484.10 ± 48.97a	494.02 ± 44.63a	4.95 ± 0.11a	5.03 ± 0.10a
WO	1071.67 ± 564.35b	70.00 ± 35.10b	0.98 ± 0.01a	91.70 ± 47.00b	110.32 ± 51.25c	2.54 ± 0.51b	2.62 ± 0.53b
GA	1433.33 ± 117.02b	113.67 ± 24.10b	0.97 ± 0.00a	176.82 ± 28.97b	243.01 ± 15.87b	3.07 ± 0.29b	3.18 ± 0.30b
ON	2093.33 ± 995.73ab	110.00 ± 26.84b	0.97 ± 0.01a	165.59 ± 26.48b	200.50 ± 35.24bc	3.04 ± 0.30b	3.14 ± 0.27b

Note: Nseqs, number of sequences analyzed; OTU, operational taxonomical unit; ACE, abundance-based coverage estimator. Values are means ± standard error (n = 3). Different letters indicate statistically significant differences at the 0.05 probability level according to Fisher’s least significant difference (LSD) test. CL: Chinese leek; WO: Welsh onion; GA: Garlic; ON: onion.

### Microbial diversity in CL was higher than that in the other three *Allium* species

The Alpha diversity of the bacterial communities was calculated using MOTHUR. The numbers of sequences (nseqs) (F = 3.79, P = 0.0584) clustered within OTUs in each of the *Allium* species were not statistically different, although the OTUs (F = 20.69, P = 0.0004) detected in CL were significantly higher than those in the other three *Allium* species. The microbial richness parameters such as Chao estimator (F = 19.67, P = 0.0005) and abundance-based coverage estimator (ACE) (F = 17.67, P = 0. 0007) of CL samples were higher than those of GA, ON and WO. The microbial diversity parameters including Shannon (F = 9.94, P = 0.0045) and npshannon (F = 9.7, P = 0.0048) indexes were also statistically higher in CL than those in other three other *Allium* species. These indicated that the richness and diversity of bacterial communities in CL were significantly higher than those in the other three *Allium* species **(**Table 2).

### Bacteria in CL was more abundant than those in the other three *Allium* species at various taxonomic levels

**Figure 2:**
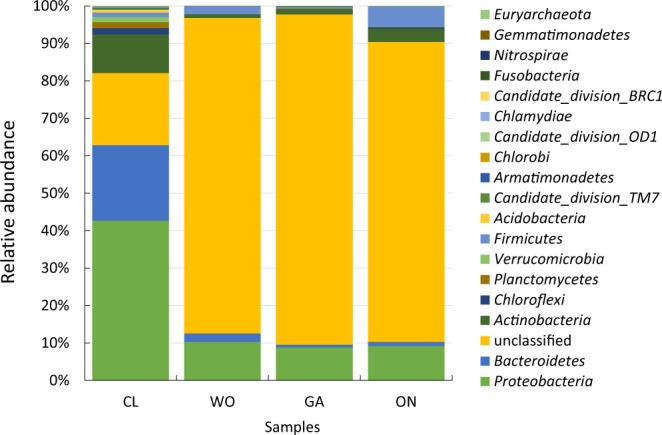
The relative abundance of bacterial phyla in the four *Allium* species. CL: Chinese leek; WO: Welsh onion; GA: Garlic; ON: onion.

**Figure 3:**
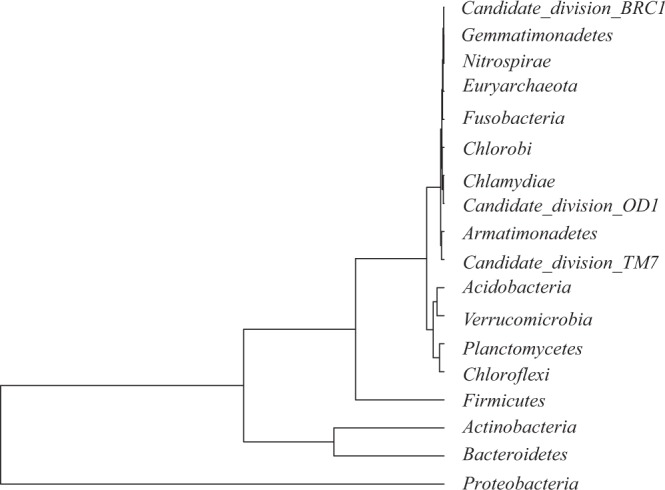
Hierarchical cluster tree of bacterial phyla in the four *Allium* species.

The OTUs were classified into 20 phyla, 50 classes, 81 orders, 134 families, and 234 genera (Supplementary Table S1). Among the bacterial communities with relative amounts more than 1.0% of total abundance, 7 phyla, 11 classes, 14 orders, and 18 families were significantly different (Supplementary Table S2). *Proteobacteria*, *Bacteroidetes*, and an unclassified phylum were the three dominant phyla, accounting for 80.31, 94.72, 80.13 and 87.27% of the total bacterial communities in CL, WO, GA, and ON, respectively (Fig. 2). Except for the unclassified phylum, all phyla in CL were significantly more abundant than those in the other three *Allium* species (Supplementary Table S2). Based on their relative abundances, all the bacterial phyla were clustered into three groups: *Proteobacteria* was clustered into one independent group, *Bacteroidetes* and *Actinobacteria* were clustered into the second group, and the other phyla were gathered into the third group (Fig. 3). The abundance of one unclassified bacterial community in CL *was* significantly lower than that in the other *Allium* species at the class (F = 16.84, P = 0.0008), order (F = 16.84, P = 0.0008), and family (F = 16.84, P = 0.0008) levels. while the abundances of other bacterial communities in CL were significantly higher than those in the other three *Allium* species (Supplementary Table S2).

**Figure 4:**
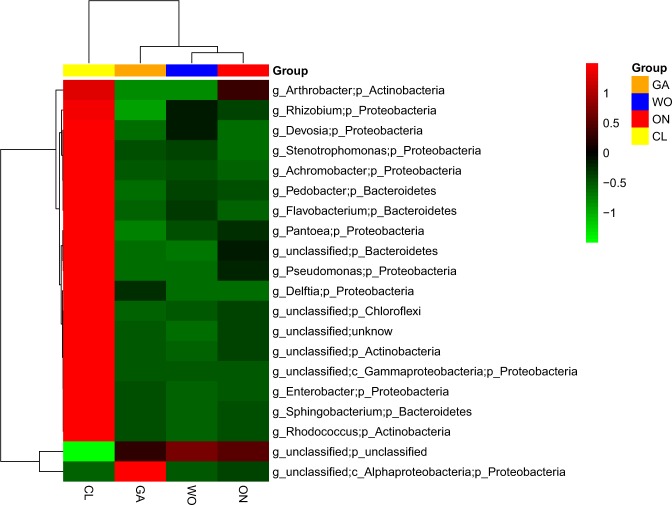
The heatmap displaying the relative abundances of the most dominant genera (top 20, accounting for more than 1.0% total abundance). CL: Chinese leek; WO: Welsh onion; GA: Garlic; ON: onion.

At the genus level, a total of 234 genera were detected in the four *Allium* species. Among these, 20 genera with relative amounts larger than 1.0% were selected for further analysis. The selected genera, belonging to 5 phyla, accounted for 62.61, 88.97, 90.97, and 86.04% in CL, WO, GA, and ON, respectively. Among these, 10 genera belonged to *Proteobacteria*, 4 to *Bacteroidetes*, 3 to *Actinobacteria*, 1 to *Chloroflexi* and 2 to the unclassified phylum. The heatmap showed all the genera were enriched in CL except for the 2 unclassified genera (g_unclassified;p_unclassified and g_unclassified;c_*Alphaproteobacteria*;p_*Proteobacteria*) (Fig. 4). The statistical analysis showed 15 of the 20 selected genera were significantly different among the four *Allium* species. The abundance of one unclassified genus (g_unclassified;p_unclassified) in CL (17.45%) was significantly lower than that in WO (82.12%), GA (70.56%) and ON (76.95%) (F = 16.84, P = 0.0008), while the other 14 genera in CL were significantly higher than those in the other three *Allium* species (Supplementary Table S2).

### Bacterial communities in WO, GA, and ON were more similar than those in CL samples

**Figure 5:**
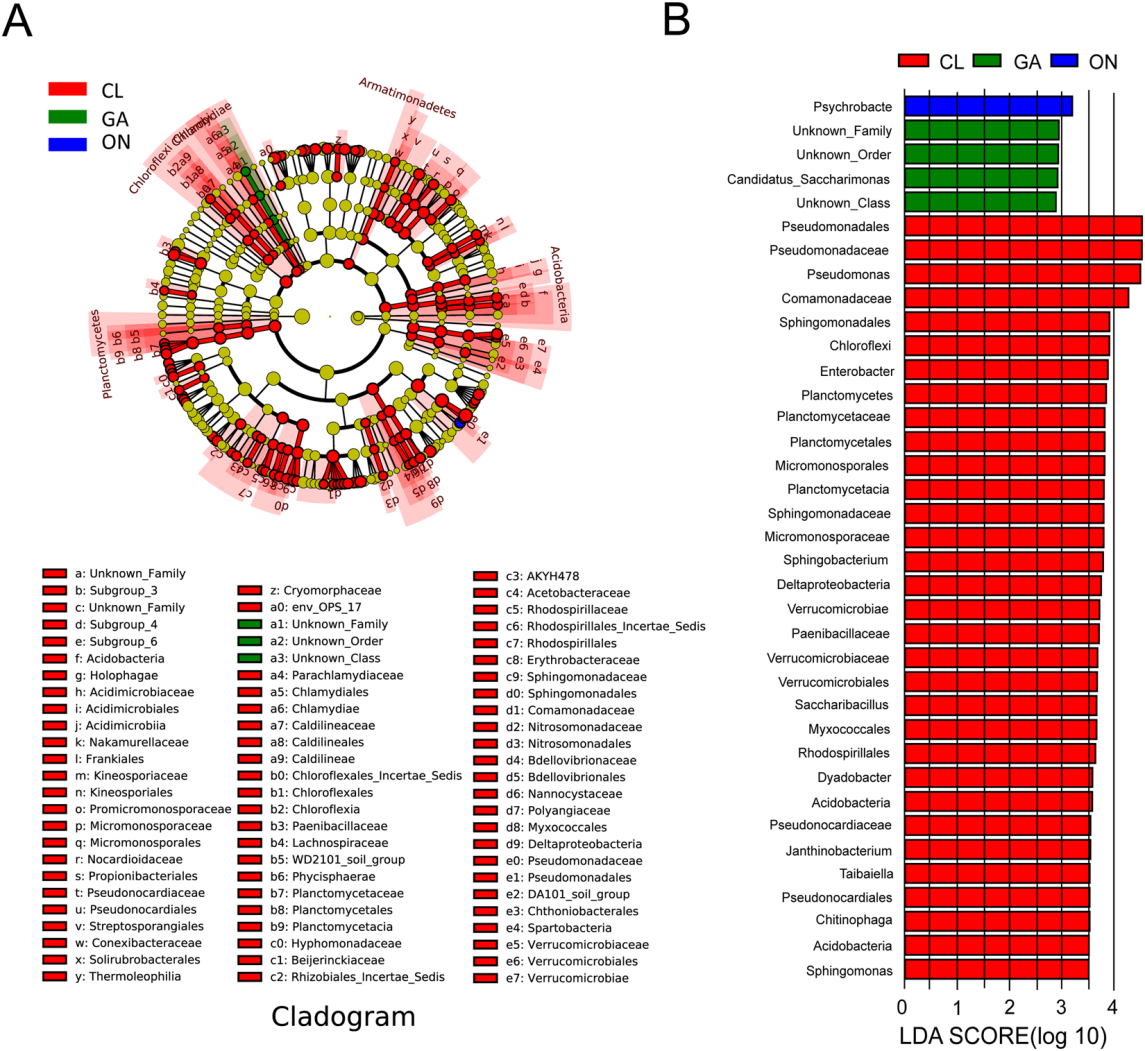
Groups from the phylum-to-genus levels determined to be significant representatives of their sample group, based on LEfSe software analysis. (**A**) Cladogram representing the taxonomic hierarchical structure of the identified habitat biomarkers generated using LEfSe. Each ring represents a taxonomic level, with phylum, class, order, and family emanating from the center to the periphery. Each circle is a taxonomic unit in the dataset, with circles or nodes shown in color where the taxon represents a significantly more abundant group. (**B**) Identified biomarkers ranked by their effect size in different samples. The habitat biomarkers were identified as being significantly abundant (p < 0.05) when compared among samples. CL: Chinese leek; WO: Welsh onion; GA: Garlic; ON: onion.

Important distinctions existed in the compositions of bacterial communities among the four *Allium* species. The LDA Effect Size (LEfSe) (Fig. 5) showed significantly different taxonomic abundances among the four *Allium* species. Interestingly, at the phylum-to-family level, bacterial communities in CL exhibited relatively higher abundances than those in the other three *Allium* species. Five phyla (including *Chloroflexi* and *Planctomycetes*), 13 classes (including *Deltaproteobacteria*, *Verrucomicrobiae*, *Acidobacteria*), 25 orders (including *Micromonosporales*, *Propionibacteriales*, and *Pseudonocardiales*), and 36 families (including *Micromonosporaceae*, *Nocardioidaceae*, and *Pseudonocardiaceae*) were significantly enriched in the CL samples (Fig. 5A). These abundant taxa could be considered as potential biomarkers (LDA > 3.5, P < 0.05) (Fig. 5B).

**Figure 6:**
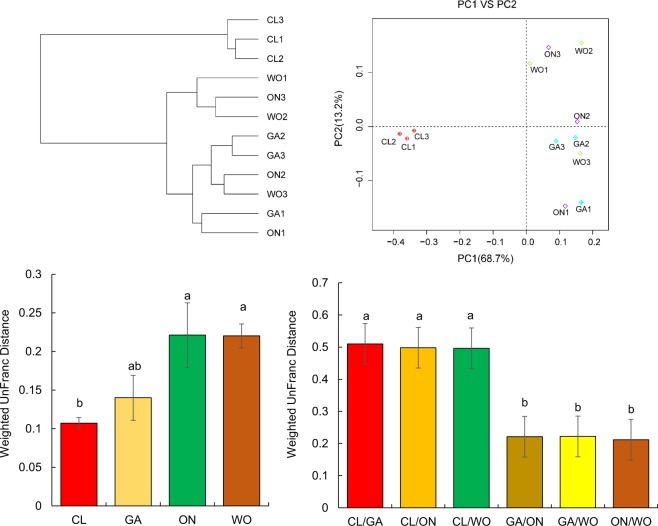
The endophytic bacterial profile of the four *Allium* species. Hierarchical cluster tree (**A**) and principal coordinate analysis (PCA) (**B**) of different microbiota in different samples based on weighted UniFrac distances. Comparison of within (**C**) and between (**D**) group distances for the *Allium* species. CL: Chinese leek; WO: Welsh onion; GA: Garlic; ON: onion.

To compare overall microbial communities between the four *Allium* species, beta-diversity analysis based on a phylogenetic tree (Fig. 6A) and PCA (Fig. 6B) was conducted using weighted UniFrac phylogenetic distances. The phylogenetic tree indicated that the 12 *Allium* samples were clustered into two groups: group 1 consisted of 3 samples of CL; group 2 consisted of 9 samples of WO, GA, and ON. The highest PCA variations in the bacteria were 68.7% (PC1) and 13.2% (PC2), representing a strong separation of different samples. The samples of CL were clustered together and distinguished from those of the other three *Allium* species which were overlapped with each other. Furthermore, comparison of within- and between-group distances for all *Allium* samples revealed that CL tended to be the most homogeneous, while ON was the most heterogenous (Fig. 6C), and that WO, GA, and ON samples were more alike than CL samples (Fig. 6D). B-diversity indicated that bacterial structure and composition of CL was significantly different from those of WO, GA, and ON.

### The predicted KEGG pathways in CL is more abundant than those in the other three Allium species

**Figure 7:**
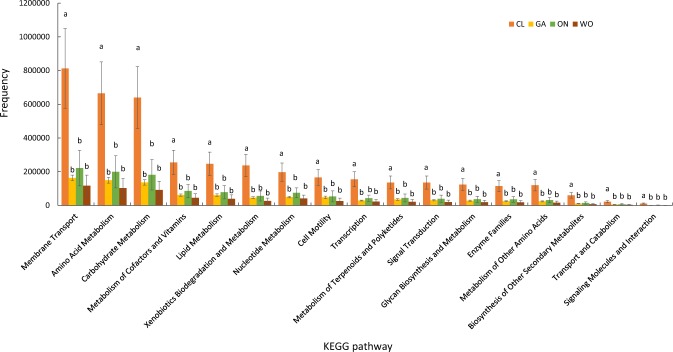
KEGG pathways with significant differences among the four *Allium* species. CL: Chinese leek; WO: Welsh onion; GA: Garlic; ON: onion.

Microbiota functions were predicted using the PICRUSt algorithm based on the Greengenes database. A total of 23 relevant KEGG categories were predicted, including Cellular Processes, Environmental Information Processing, Genetic Information Processing and Metabolism. The frequencies ranged from 24 to 3,944,197 (Supplementary Table S3). 17 of these KEGG pathways in CL exhibited significantly higher abundance than those in the other three *Allium* species. (Supplementary Table S3). Membrane Transport (F = 5.98, P = 0.0193), Amino Acid Metabolism (F = 5.73, P = 0.0216), Carbohydrate Metabolism (F = 5.76, P = 0.0213), Metabolism of Cofactors and Vitamins (F = 5.16, P = 0.0283) and Lipid Metabolism (F = 5.16, P = 0.0282) were the five most enriched KEGG (Fig. 7, Supplementary Table S3).

To further identify microbiota function in the four *Allium* species, we analyzed **206** lower-level KEGG pathways within the **23** KEGG categories the results of which showed that 1**59** of the lower-level KEGG pathways (accounting for **71.81**% of the total abundance) were significantly different among the four *Allium* species (Supplementary Table S4). Interestingly, all these KEGG pathways in CL, containing Transporters (F = 6.68, P = 0.0143), ABC transporters (F = 6.29, P = 0.0169), Secretion system (F = 4.50, P = 0.0395), Peptidases (F = 5.57, P = 0.0233), Streptomycin biosynthesis (F = 6.62, P = 0.0147), Sulfur metabolism (F = 7.68, P = 0.0097) and Steroid hormone biosynthesis (F = 8.19, P = 0.0080), were also significantly higher than those in the other three *Allium* species. Otherwise, the **47** KEGG categories were not statistically different among the four *Allium* species (Supplementary Table S4).

### Bacteria were very closely related to genes

**Figure 8:**
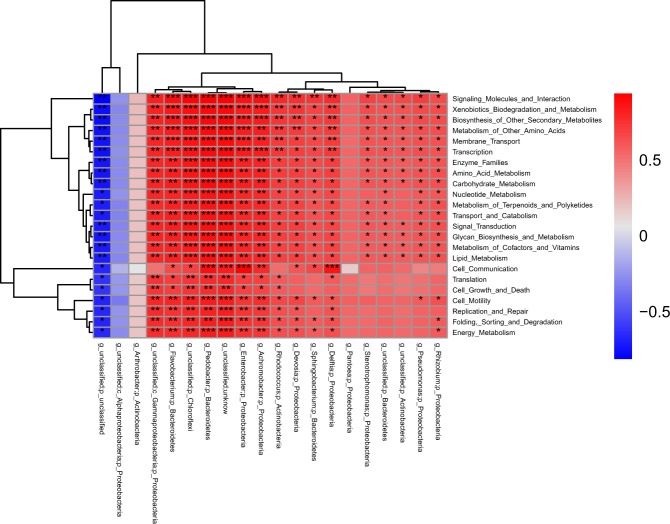
KEGG pathways with significant differences among the four *Allium* species were highly correlated with bacterial genera with significant differences. *0.01 < p < 0.05, **0.001 < p < 0.01, ***p < 0.001.

To clarify the relationship among the bacterial communities and predicted bacterial functions, the correlation of the 20 bacterial genera (>1.0%) and the 23 KEGG categories were analyzed using the Pearson method. The results showed that the KEGG pathways were firmly related to bacterial genera. Among these relations, 100 pairs comprised of 9 genera and 21 KEGG pathways showed a very significant correlation (r > 0.8, P < 0.01) (Supplementary Table S5, Fig. 8).

## Discussion

In the present study, we compared the endophytic bacterial communities between the four *Allium* species: CL, GA, ON, and WO by using high-throughput sequencing technology. The results showed that the bacterial communities in CL were significantly different from those in the other three *Allium* species. The diversity and richness of endophytic bacterial communities in CL were more abundant than those in the GA, WO and ON. Also, the predicted gene functions of endophytic bacteria mainly focused on Membrane Transport, Amino Acid Metabolism, and Carbohydrate Metabolism pathway.

*Proteobacteria* was core phyla in the plant endosphere. In a review, it summarized that *Proteobacteria* was the dominant phylum, the average abundance of which was approximately 50%^1^. Other previous studies also supported this conclusion. Endophytic *Proteobacteria* in apple shoots of different rootstock/scion combinations was as high as 58.4%^20^, and *Proteobacteria* comprised approximately 42.40–64.70% of reads across all roots and leaves of the peony tree^21^. *Proteobacteria* was also the most abundant phylum (90%) in the leaves of tomato^22^, and it represented 84% of the total bacteria in sweet clover root^23^. The results of our present study showed that the endophytic bacterial community was significantly different in the four *Allium* species. At the phylum level, *Proteobacteria* was the dominant bacterial community in CL (42.68%), and also the dominant bacterial community in WO (10.31%), GA (8.80%), and ON (9.20%). However, the amount in the three *Allium* species was significantly lower than that in CL (F = 20.21, P = 0.0004).

Our study revealed that one unclassified phylum was the most dominant bacterial community in WO (82.12%), GA(70.56%) and ON(76.95%) besides *Proteobacteria*, the abundance of which was significantly higher in the three *Allium* species than that in CL (17.45%) (F = 16.84, P = 0.0008). The vast abundance of the unclassified phyla was also found in the previous studies^24^. On the one hand, these unclassified bacterial species reflected the advantages of high-throughput sequencing technology to detect minor, rare, and uncultured bacterial species. On the other hand, it showed that *Allium* species were abundant in the beneficial bacterial communities^25^.

The comparative analysis showed significant differences between the four *Allium* species at the phylum, class, order, family, and genus level. Interestingly, the majority of the different bacterial communities in CL were significantly higher than that in the other *Allium* species. The alpha diversity indicated higher bacterial richness and diversity in CL than those in the other three *Allium* species. Meanwhile, the beta-diversity analysis showed that bacterial communities in WO, GA, and ON were more similar than those in CL. This was consistent with the conclusion that the GA, ON, and WO were closer related to each other, and farther away from CL in a genetic relationship^26,27^. In China, CL was planted once and harvested continuously for 3–5 years, while GA, ON, and WO were planted once in a year and harvested once. That was to say, under this cultivation pattern, the growth period of CL in the soil was 3–5 times more than those of GA, ON and WO. The numbers of sequences (nseqs) and the operational taxonomic units (OTUs) detected in CL were 2.45–4.78 and 3.96–6.22 times higher than those in the other three *Allium* species. However, the dominant bacteria in the GA, ON and WO was more evident than that in the CL. For example, one unclassified genus was the most dominant bacteria in the four *Allium* species. The amounts accounted for 82.12, 70.56 and 76.95% in WO, GA and ON, which were 4.04–4.71 times more than that in CL, respectively. These factors, including heredity, cultivation patterns, and dominant bacteria, have contributed to the occurrence of this phenomenon.

Endophytes and host plants established a relationship of mutualistic symbiosis. The growth scale and speed of endophytes in plants were rather crucial for maintaining the relationship. Previous studies have shown that there was a balance point between endophytes and plants. Once the balance was broken, this mutualistic symbiosis would be destroyed. Endophytes obtained a large number of nutrients from plants, degraded the nutrients and cell walls of plants, and changed from mutualistic symbiosis to parasitic relationship. It might even turn into a “pathogen”^28–30^. This balance was determined by the genetic material of endophytes and plants, and it also was the result of mutual adaptation and natural selection. So the different endophytic bacterial communities in four *Allium* species might be formed through long-term evolution and were also a necessary condition for maintaining the mutualistic symbiosis relationship between endogenous bacteria and plants.

In the present study, the microbiota function analysis revealed the endophytic bacteria were annotated in 23 KEGG categories. Membrane transport was the richest pathway. It included three lower-level pathway: Transporters, ABC transporters, and secretion system. Transport protein/ABC transporter is vital for bacterial growth and survival for their nutrient absorption and resistance to endogenous and environmental pressures^31,32^. In plants, it participates in the transport of many substances, such as hormones, lipids, secondary metabolites, and exogenous organisms. It also participates in the interaction between plants and pathogens and the regulation of ion channels^33^. Secretion system can deliver bacterial effector proteins into host cells to kill or inhibit their growth, and help the bacteria thrive in its host^34,35^. That is to say, the three pathways are closely related to the survival of endophytic bacteria and host plants. This explained why Transporters, ABC transporters, and secretion system were the three most enriched pathway among the 206 lower-lever pathways in our study.

Amino acids are not only the main components of proteins but also play an essential role in many other physiological processes. They have osmotic effects, regulate ion transport, control stomatal opening, participate in the detoxification of heavy metals, promote redox homeostasis, affect gene expression, and influence the synthesis and activity of some enzymes. Also, amino acids are precursors of many secondary metabolites, which have vital functions such as signal, defense, interaction with other organisms, and photoprotection^36^. Glycolysis and the citric acid cycle (TCA cycle) are key respiratory pathways in carbohydrate metabolism. They provide plant and bacteria with energy and metabolites for growth and development. Besides, they produce many intermediates for synthesizing other substances^37,38^. In our study, amino acid metabolism and carbohydrate metabolism pathway were the second and third most enriched pathway in the 23 KEGG categories, which revealed that the majority of the endophytic bacteria of the four *Allium* species are involved in the two pathways. That may because endophytic bacteria live on the host plants and form a symbiotic relationship with the host. The growth of endophytic bacteria requires various nutrient sources and energy. They participate in the synthesis, degradation, and absorption of amino acids and carbohydrate in the host to provide the nutrient sources and energy for its growth, on the other hand, provide essential amino acids and energy for the host to meet the physiological needs.

*Allium* plants are known to possess antibacterial and antifungal properties, which are due to their organic sulfides, especially thiosulfinates^39^. Thiosulfinates decomposed into various sulfur compounds with potent antimicrobial properties^19,40^. The results showed that endophytic bacteria possess sulfur metabolism function, and it may help the host to regulate the organosulfur compounds.

Besides the well-known sulfur compounds, there exist other kinds of compounds such as saponins, flavonoids, nitrogen compounds, and peptides in *Allium* species with vigorous inhibitive effects on pathogenic microbes^19^. Our results showed the endophytic bacteria also involved in other important physiological activities such as peptidases, nitrogen metabolism, terpenoid backbone biosynthesis, streptomycin biosynthesis, tetracycline biosynthesis, biosynthesis of ansamycins, and steroid hormone biosynthesis. Previous studies pointed out that plants and endophytes may have similar or identical pathways for the synthesis of secondary metabolites. Moreover, they can synthesize the same or similar active substances^30^. Therefore, all these multifunctional endophytic bacteria corporately regulated the metabolism of organosulfur compounds, secondary metabolites, and antibiotics, which demonstrated the antimicrobial properties of *Allium* species.

PICRUSt can predict the corresponding bacterial metabolic function spectrum through the 16S rRNA gene sequence^41^. PICRUSt can unscramble potential functions from the microbiome composition data. With PICRUSt, it acts as a bridge between the “composition” and “function” of the microbiome. However, the greengenes reference database tends to study human-related microorganisms, which reduces the accuracy of predicting non-human metagenomes. In our present study, to test the accuracy of the bacterial function prediction, we performed the correlations between bacterial genera and the KEGG pathways, the results revealed they were closely related to each other. For example, *Achromobacter* spp. were predicted to possess the function of Xenobiotics Biodegradation and Metabolism (r = 0.8346, P = 0.0007). The previous studies reported *Achromobacter* spp. is efficient in degrading atrazine^42^; glyphosate^43^; 2,4-dichlorophenoxyacetic acid (2,4-D) and 2-methyl-4-chlorophenoxy acetic acid (MCPA)^44^. *Enterobacter* spp. were estimated to have the function of Biosynthesis of Other Secondary Metabolites (r = 0.836, P = 0.0007) and Amino Acid Metabolism (r = 0.810, P = 0.0014). Previous studies demonstrated *Enterobacter* spp. produce secondary metabolites such as siderophores and indole-3-acetic acid(IAA)^45–47^, and enzymes such as pectinase, 1-ami-nocyclopropane-1-carboxylate(ACC) deaminase, and arginine decarboxylase^46,48,49^. *Flavobacterium* spp. were estimated to have the function Biosynthesis of Other Secondary Metabolites (r = 0.8445, P = 0.0005) and Glycan Biosynthesis and Metabolism (r = 0.8157, P = 0.0012). The previous studies showed that *Flavobacterium* spp. synthesized carotenoid zeaxanthin^50^, produced indolic compounds and biosurfactant^51^; *Flavobacterium* spp. also digests alginate oligosaccharides^52^ and many polysaccharides, including chitin^53^. So all these previous studies indicated that the predicted function was consistent with the actual function of endophytic bacteria.

## Methods

### Sample collection

GA, ON, WO, CL were collected from the experimental base of the Qingdao Agricultural University located at Jiaozhuo, Qingdao city, China. The four *Allium* species were cultured according to conventional agricultural practices, and the underground parts of each plant were used to analyze the endophytic bacterial communities. Individually, the bulbs of garlic and onion, the white pseudostem of Welsh onion, and the rhizome of Chinese leek were analyzed. Three biological replications were sampled; each consisted of five individual plants.

Sample pretreatment was carried out according to references with minor modifications^25^. The sampled bulbs, pseudostems, and rhizomes were washed using tap water and cut into small pieces of 2-cm long. Then, they were soaked in 70% ethyl alcohol for 2 min, immersed in 2.5% sodium hypochlorite for 5 min, and finally washed with sterile water 4 times. From the fourth wash, 100 μl of the water was added to potato dextrose agar (PDA) plates and incubated at 28 °C for 7 d to determine the disinfection effect. Disinfection grade experiment materials were used to analyze the endophytic bacterial community.

### DNA extraction, PCR amplification, and Illumina library generation

DNA of the four *Allium* species was extracted using TIANamp Bacteria DNA Kit(Tiangen Biotech, Beijing, China) according to manufacturer’s instructions and stored at −20 °C until analysis. The target-specific primers B341F (5′-CCTACGGGNGGCWGCAG-3′) and B785R (5′-GACTACHVGGGTATCTAATCC-3′) were used to amplify the V3-V4 region of the bacterial 16S rRNA gene^25,54^. PCR reaction referenced to our previous study with minor modifications^25^. Each PCR reaction was performed using a KAPA HiFi Hotstart PCR Kit (KAPA Biosystems, Wilmington, MA, USA) on a T100^TM^ Thermal Cycler (Bio-Rad, Hercules, CA, USA). Each reaction (25 μL) contained 10 ng of DNA template, 12.5 μL 2 × KAPA HiFi HotStart ReadyMix, and 0.25 μmol L^−1^ of each primer. The following PCR conditions were used: initial denaturation at 95 °C for 3 min, followed by 25 cycles consisting of denaturation (95 °C for 30 s), annealing (55 °C for 30 s) and extension (72 °C for 30 s) and a final extension step at 72 °C for 5 min. PCR amplicon libraries were purified from a 1.2% agarose gel by QIAquick Gel Extraction Kit (Qiagen, Hilden, Germany) and quantified using the KAPA Library Quantification Kit (KAPA Biosystems, Wilmington, MA, USA), and finally sequenced using the Illumina MiSeq platform (Illumina Inc., San Diego, CA, USA) at Beijing Ori-Gene Science and Technology (China) to create paired-end reads. The raw reads have been deposited at the Sequence Read Archive (SRA) database of NCBI (SRR8240026 to SRR8240037).

### Sequence processing and analysis

FLASH^55^ was used to merge the paired-end reads. The combined reads were filtered using MOTHUR^56^ to remove sequences with low quality scores (≤20), to remove sequences with N, to remove sequences with too long homopolymer (>10 bp), to remove sequences with excessive primer mismatch (≥4 bp) and the primer sequence, to remove sequences that are too short (≤200 bp) and too long (≥500 bp). Moreover, using UCHIME^57^ to remove chimera with Gold dataset as reference^58^. The filtered sequences were clustered into operational taxonomic units (OTUs) at 97% identity using USEARCH^59^. Each OTU was systematically classified with the reference of the SILVA database^60^ using RDP classifiers^61^. The diversity indices for each species were analyzed using MOTHUR^56^.

OTUs were aligned to SILVA database^60^ using PyNAST software^62^ to construct a phylogenetic tree using FastTree^63^. UniFrac software^64^ was used to generate the weighted distance matrix among bacterial communities. Furthermore, we used the LDA Effect Size (LEfSe) to identify differentially abundant families among samples for biomarker discovery^65^. We also predicted the functional profiling of microbial communities using the Phylogenetic Investigation of Communities by Reconstruction of Unobserved States (PICRUSt) algorithm^41^. The 16S rRNA sequences were assigned with the Greengenes database to perform closed reference picking, and then the resulting sequences were uploaded onto the PICRUSt website (http://huttenhower.sph.harvard.edu/galaxy/), to generate predicted functional metagenomes using the Kyoto Encyclopedia of Genes and Genomes (KEGG) database^66,67^ as a functional reference.

### Statistical analysis

The data were analyzed using a one-way ANOVA in SAS ver. 8.0 software. Significance differences among the four *Allium* species were determined using Fisher’s least significant difference test (P ≤ 0.05). Venn diagrams were generated using the “Venn diagram” package, heatmap images were produced using the “pheatmap” package, and Principal coordinate analysis (PCA) figures were created using the “princomp” program in R (v3.1.2). LefSe and PICRUSt were analyzed using LefSe (https://bitbucket.org/biobakery/biobakery/wiki/lefse) and PICRUSt software (http://huttenhower.sph.harvard.edu/galaxy/), respectively. Pearson’s correlations were used to assess the relationships between gene functions and bacterial communities.

## Supplementary information

Spplementary Figure S1, table S1-5

## Data Availability

Data are available from the Sequence Read Archive (SRA) database of NCBI (SRR8240026 to SRR8240037) https://www.ncbi.nlm.nih.gov/bioproject/PRJNA506734.
