# Supplementary material for: Illumina-based Analysis of Endophytic Bacterial Diversity of four Allium species
Source: Sci Rep. 2019 Oct 24;9:15271. doi: 10.1038/s41598-019-51707-7 (PMC6813343; doi:10.1038/s41598-019-51707-7)
Supplement: Supplementary file 1 — Spplementary Figure S1, table S1-5 [file 41598_2019_51707_MOESM1_ESM.pdf]

Illumina-based Analysis of Endophytic Bacterial Diversity of four *Allium* species

Yonghong Huang

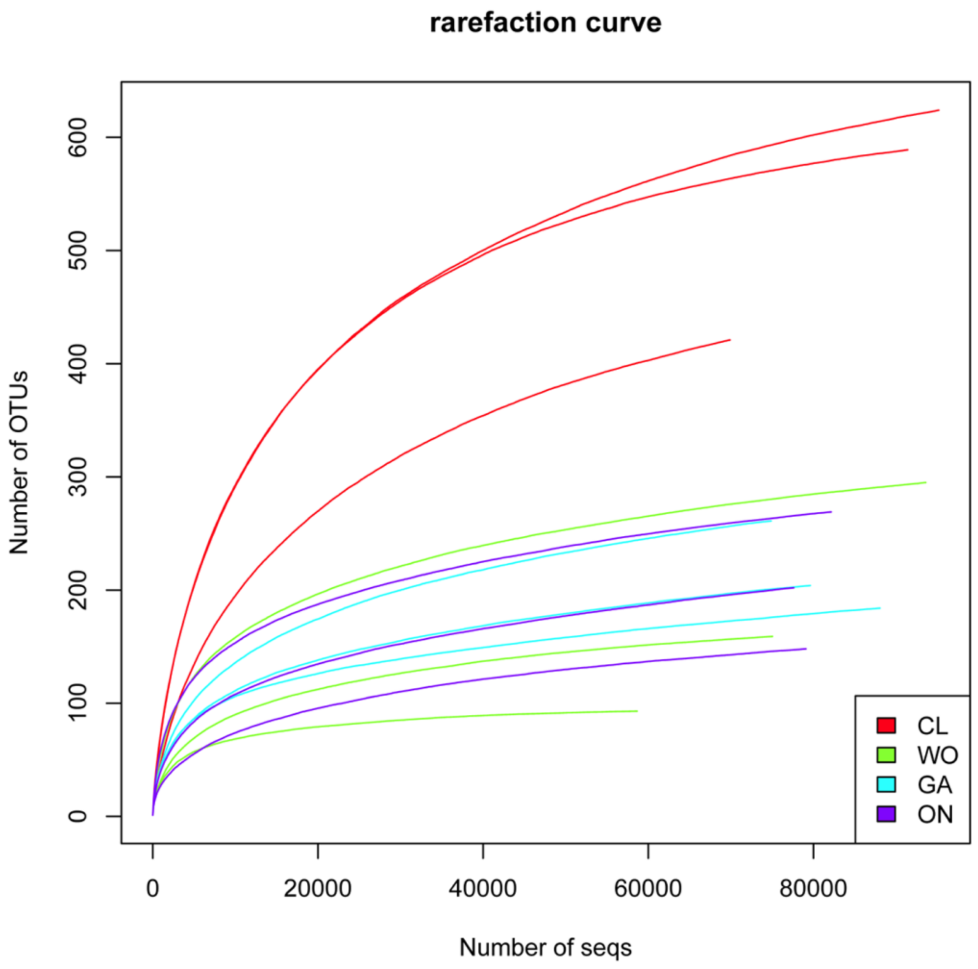

Supplementary Figure S1. Rarefaction curves based on the sequences of the V4–V5 region of the 16S rRNA gene from four *Allium* species CL: Chinese leek; WO: Welsh onion; GA: Garlic; ON: Onion.

Spplementary table S1 Bacterial species indentified from the four Allium spp.

taxlevel	rankID	taxon	daughterlevels	mean	CL1	CL2	CL3	WO1	WO2	WO3	GA1	GA2	GA3	ON1	ON2	ON3
0	0	*Root*	3	100%	100%	100%	100%	100%	100%	100%	100%	100%	100%	100%	100%	100%
1	0.1	*Archaea*	1	0.02%	0.03%	0%	0%	0%	0%	0%	0%	0%	0.07%	0%	0%	0.15%
1	0.2	*Bacteria*	18	93.82%	98.98%	95.88%	99.68%	100%	99.45%	94.33%	96.80%	75.44%	74.92%	93.47%	97.68%	99.26%
1	0.3	*unknown*	1	6.16%	0.99%	4.12%	0.32%	0%	0.55%	5.67%	3.20%	24.56%	25.02%	6.53%	2.32%	0.60%
2	0.1.1	*Euryarchaeota*	1	0.02%	0.03%	0%	0%	0%	0%	0%	0%	0%	0.07%	0%	0%	0.15%
2	0.2.1	*Acidobacteria*	2	0.20%	0.64%	1.01%	0.64%	0%	0%	0.09%	0%	0%	0.07%	0%	0%	0%
2	0.2.2	*Actinobacteria*	4	4.05%	8.62%	10.05%	12.15%	1.96%	0%	1.10%	0.75%	1.24%	2.20%	1.20%	1.00%	8.33%
2	0.2.3	*Armatimonadetes*	2	0.09%	0.18%	0.46%	0.23%	0%	0%	0%	0%	0%	0.07%	0.05%	0.06%	0%
2	0.2.4	*Bacteroidetes*	5	6.09%	22.31%	20.90%	17.33%	6.17%	0%	0.69%	0.31%	0.41%	1.60%	1.22%	1.25%	0.89%
2	0.2.5	*Candidate_division_BRC1*	1	0.01%	0%	0.05%	0.05%	0%	0%	0%	0%	0%	0%	0%	0%	0%
2	0.2.6	*Candidate_division_OD1*	1	0.03%	0.22%	0.12%	0%	0%	0%	0%	0%	0%	0%	0.07%	0%	0%
2	0.2.7	*Candidate_division_TM7*	2	0.12%	0.31%	0.18%	0.41%	0%	0%	0%	0.19%	0.17%	0.07%	0.10%	0%	0%
2	0.2.8	*Chlamydiae*	1	0.02%	0.14%	0.08%	0.05%	0%	0%	0%	0%	0%	0%	0%	0%	0%
2	0.2.9	*Chlorobi*	2	0.03%	0.06%	0.05%	0.28%	0%	0%	0%	0%	0%	0%	0%	0%	0%
2	0.2.10	*Chloroflexi*	8	0.54%	1.25%	1.97%	1.97%	0%	0%	0%	0%	0.08%	0.13%	0.22%	0%	0.89%
2	0.2.11	*Firmicutes*	3	2.23%	1.74%	0.76%	1.05%	0.15%	0%	6.13%	0.25%	0.25%	0.60%	14.81%	0.63%	0.45%
2	0.2.12	*Fusobacteria*	1	0.05%	0.06%	0%	0%	0%	0%	0.09%	0.13%	0.17%	0%	0%	0%	0.15%
2	0.2.13	*Gemmatimonadetes*	1	0%	0.01%	0.03%	0%	0%	0%	0%	0%	0%	0%	0%	0%	0%
2	0.2.14	*Nitrospirae*	1	0%	0%	0.05%	0%	0%	0%	0%	0%	0%	0%	0%	0%	0%
2	0.2.15	*Planctomycetes*	3	0.46%	0.99%	2.22%	1.56%	0%	0%	0.05%	0.06%	0.08%	0.13%	0.07%	0%	0.30%
2	0.2.16	*Proteobacteria*	5	17.75%	44.24%	41.34%	42.46%	22.89%	2.20%	5.85%	3.77%	8.05%	14.57%	6.46%	8.33%	12.80%
2	0.2.17	*Verrucomicrobia*	5	0.37%	1.02%	2.22%	0.73%	0%	0%	0.05%	0%	0%	0.13%	0.12%	0%	0.15%
2	0.2.18	*unclassified*	1	61.77%	17.18%	14.39%	20.77%	68.83%	97.25%	80.29%	91.33%	64.98%	55.36%	69.14%	86.40%	75.30%
2	0.3.1	*unclassified*	1	6.16%	0.99%	4.12%	0.32%	0%	0.55%	5.67%	3.20%	24.56%	25.02%	6.53%	2.32%	0.60%
3	0.1.1.1	*Halobacteria*	1	0.02%	0.03%	0%	0%	0%	0%	0%	0%	0%	0.07%	0%	0%	0.15%
3	0.2.1.1	*Acidobacteria*	3	0.16%	0.55%	0.69%	0.55%	0%	0%	0.09%	0%	0%	0.07%	0%	0%	0%
3	0.2.1.2	*Holophagae*	1	0.04%	0.10%	0.31%	0.09%	0%	0%	0%	0%	0%	0%	0%	0%	0%
3	0.2.2.1	*Acidimicrobiia*	1	0.13%	0.35%	0.60%	0.37%	0%	0%	0%	0%	0%	0%	0.05%	0%	0.15%
3	0.2.2.2	*Actinobacteria*	10	3.81%	7.85%	8.96%	11.28%	1.96%	0%	1.10%	0.75%	1.24%	2.20%	1.15%	1.00%	8.18%
3	0.2.2.3	*Thermoleophilia*	1	0.10%	0.35%	0.41%	0.46%	0%	0%	0%	0%	0%	0%	0%	0%	0%
3	0.2.2.4	*unclassified*	1	0.02%	0.07%	0.08%	0.05%	0%	0%	0%	0%	0%	0%	0%	0%	0%
3	0.2.3.1	*Armatimonadia*	1	0.01%	0%	0.03%	0.09%	0%	0%	0%	0%	0%	0%	0%	0%	0%
3	0.2.3.2	*unclassified*	1	0.08%	0.18%	0.43%	0.14%	0%	0%	0%	0%	0%	0.07%	0.05%	0.06%	0%
3	0.2.4.1	*Bacteroidia*	1	0.05%	0.04%	0%	0.09%	0.15%	0%	0%	0%	0.17%	0%	0%	0.19%	0%
3	0.2.4.2	*Cytophagia*	1	0.51%	1.68%	1.98%	1.56%	0.30%	0%	0.23%	0%	0%	0.20%	0.17%	0%	0%
3	0.2.4.3	*Flavobacteriia*	1	3.37%	13.28%	10.34%	9.40%	4.67%	0%	0.27%	0.25%	0.08%	0.86%	0.62%	0.38%	0.30%
3	0.2.4.4	*Sphingobacteriia*	1	2.12%	7.05%	8.40%	6.24%	1.05%	0%	0.18%	0.06%	0.17%	0.53%	0.42%	0.69%	0.60%
3	0.2.4.5	*unclassified*	1	0.04%	0.25%	0.18%	0.05%	0%	0%	0%	0%	0%	0%	0%	0%	0%
3	0.2.5.1	*unclassified*	1	0.01%	0%	0.05%	0.05%	0%	0%	0%	0%	0%	0%	0%	0%	0%
3	0.2.6.1	*unclassified*	1	0.03%	0.22%	0.12%	0%	0%	0%	0%	0%	0%	0%	0.07%	0%	0%
3	0.2.7.1	*Unknown_Class*	1	0.02%	0.01%	0%	0%	0%	0%	0%	0.06%	0.08%	0.07%	0.02%	0%	0%
3	0.2.7.2	*unclassified*	1	0.10%	0.29%	0.18%	0.41%	0%	0%	0%	0.13%	0.08%	0%	0.07%	0%	0%
3	0.2.8.1	*Chlamydiae*	1	0.02%	0.14%	0.08%	0.05%	0%	0%	0%	0%	0%	0%	0%	0%	0%
3	0.2.9.1	*Chlorobia*	1	0%	0.04%	0.02%	0%	0%	0%	0%	0%	0%	0%	0%	0%	0%
3	0.2.9.2	*unclassified*	1	0.03%	0.01%	0.03%	0.28%	0%	0%	0%	0%	0%	0%	0%	0%	0%
3	0.2.10.1	*Anaerolineae*	1	0.01%	0%	0.02%	0.05%	0%	0%	0%	0%	0%	0%	0.02%	0%	0%
3	0.2.10.2	*Caldilineae*	1	0.01%	0.06%	0.05%	0.05%	0%	0%	0%	0%	0%	0%	0%	0%	0%
3	0.2.10.3	*Chloroflexia*	1	0.07%	0.21%	0.15%	0.23%	0%	0%	0%	0%	0.08%	0%	0%	0%	0.15%
3	0.2.10.4	*KD4-96*	1	0.01%	0.03%	0.03%	0%	0%	0%	0%	0%	0%	0%	0%	0%	0%
3	0.2.10.5	*Ktedonobacteria*	1	0.01%	0%	0%	0%	0%	0%	0%	0%	0%	0%	0.12%	0%	0%
3	0.2.10.6	*S085*	1	0.03%	0.06%	0.15%	0.09%	0%	0%	0%	0%	0%	0.07%	0%	0%	0%
3	0.2.10.7	*Thermomicrobia*	2	0.38%	0.74%	1.37%	1.51%	0%	0%	0%	0%	0%	0.07%	0.07%	0%	0.74%
3	0.2.10.8	*unclassified*	1	0.03%	0.15%	0.20%	0.05%	0%	0%	0%	0%	0%	0%	0%	0%	0%
3	0.2.11.1	*Bacilli*	2	2.19%	1.65%	0.71%	1.01%	0.15%	0%	5.99%	0.13%	0.25%	0.60%	14.73%	0.63%	0.45%
3	0.2.11.2	*Clostridia*	1	0.03%	0.08%	0.05%	0.05%	0%	0%	0.09%	0%	0%	0%	0.05%	0%	0%
3	0.2.11.3	*unclassified*	1	0.02%	0%	0%	0%	0%	0%	0.05%	0.13%	0%	0%	0.02%	0%	0%
3	0.2.12.1	*Fusobacteriia*	1	0.05%	0.06%	0%	0%	0%	0%	0.09%	0.13%	0.17%	0%	0%	0%	0.15%
3	0.2.13.1	*Gemmatimonadetes*	1	0%	0.01%	0.03%	0%	0%	0%	0%	0%	0%	0%	0%	0%	0%
3	0.2.14.1	*Nitrospira*	1	0%	0%	0.05%	0%	0%	0%	0%	0%	0%	0%	0%	0%	0%
3	0.2.15.1	*Phycisphaerae*	1	0.05%	0.03%	0.31%	0.23%	0%	0%	0%	0%	0%	0%	0%	0%	0%
3	0.2.15.2	*Planctomycetacia*	1	0.39%	0.85%	1.85%	1.33%	0%	0%	0.05%	0.06%	0.08%	0.13%	0.07%	0%	0.30%
3	0.2.15.3	*unclassified*	1	0.01%	0.11%	0.05%	0%	0%	0%	0%	0%	0%	0%	0%	0%	0%
3	0.2.16.1	*Alphaproteobacteria*	7	6.43%	11.62%	14.45%	16.96%	9.34%	1.10%	2.24%	1.76%	3.90%	5.19%	2.09%	2.82%	5.65%
3	0.2.16.2	*Betaproteobacteria*	6	2.96%	7.05%	8.70%	7.11%	8.28%	0%	0.27%	0.25%	0.50%	2.26%	0.32%	0.44%	0.30%
3	0.2.16.3	*Deltaproteobacteria*	3	0.29%	0.85%	1.59%	0.92%	0%	0%	0.05%	0%	0%	0%	0.07%	0%	0%
3	0.2.16.4	*Gammaproteobacteria*	6	8.01%	24.61%	16.19%	17.29%	5.27%	1.10%	3.29%	1.76%	3.65%	7.05%	3.96%	5.08%	6.85%
3	0.2.16.5	*unclassified*	1	0.06%	0.11%	0.41%	0.18%	0%	0%	0%	0%	0%	0.07%	0%	0%	0%
3	0.2.17.1	*OPB35_soil_group*	1	0.01%	0%	0%	0%	0%	0%	0%	0%	0%	0.13%	0%	0%	0%
3	0.2.17.2	*Opitutae*	1	0.01%	0%	0.02%	0.05%	0%	0%	0%	0%	0%	0%	0%	0%	0%
3	0.2.17.3	*Spartobacteria*	1	0.07%	0.21%	0.41%	0.14%	0%	0%	0.05%	0%	0%	0%	0%	0%	0%
3	0.2.17.4	*Verrucomicrobiae*	1	0.26%	0.74%	1.60%	0.55%	0%	0%	0%	0%	0%	0%	0.12%	0%	0.15%
3	0.2.17.5	*unclassified*	1	0.02%	0.07%	0.18%	0%	0%	0%	0%	0%	0%	0%	0%	0%	0%
3	0.2.18.1	*unclassified*	1	61.77%	17.18%	14.39%	20.77%	68.83%	97.25%	80.29%	91.33%	64.98%	55.36%	69.14%	86.40%	75.30%
3	0.3.1.1	*unclassified*	1	6.16%	0.99%	4.12%	0.32%	0%	0.55%	5.67%	3.20%	24.56%	25.02%	6.53%	2.32%	0.60%
4	0.1.1.1.1	*Halobacteriales*	1	0.02%	0.03%	0%	0%	0%	0%	0%	0%	0%	0.07%	0%	0%	0.15%
4	0.2.1.1.1	*Subgroup_3*	1	0.05%	0.14%	0.17%	0.23%	0%	0%	0%	0%	0%	0.07%	0%	0%	0%
4	0.2.1.1.2	*Subgroup_4*	1	0.09%	0.20%	0.46%	0.28%	0%	0%	0.09%	0%	0%	0%	0%	0%	0%
4	0.2.1.1.3	*Subgroup_6*	1	0.03%	0.21%	0.07%	0.05%	0%	0%	0%	0%	0%	0%	0%	0%	0%
4	0.2.1.2.1	*unclassified*	1	0.04%	0.10%	0.31%	0.09%	0%	0%	0%	0%	0%	0%	0%	0%	0%

4	0.2.2.1.	*Acidimicrobiales*	2	0.13%	0.35%	0.60%	0.37%	0%	0%	0%	0%	0%	0%	0.05%	0%	0.15%
4	0.2.2.2.	*Corynebacteriales*	3	1%	2.07%	2.18%	2.71%	0.45%	0%	0.27%	0.13%	0.25%	0.80%	0.20%	0.31%	2.68%
4	0.2.2.2.	*Frankiales*	1	0.01%	0.01%	0.02%	0.09%	0%	0%	0%	0%	0%	0%	0%	0%	0%
4	0.2.2.2.	*Kineosporiales*	1	0.04%	0.07%	0.10%	0.32%	0%	0%	0%	0%	0%	0%	0%	0%	0%
4	0.2.2.2.	*Micrococcales*	7	1.70%	2.40%	3.31%	4.68%	1.51%	0%	0.64%	0.44%	0.83%	1.13%	0.60%	0.56%	4.32%
4	0.2.2.2.	*Micromonosporales*	1	0.42%	0.74%	1.32%	2.20%	0%	0%	0%	0%	0%	0.13%	0.02%	0.06%	0.60%
4	0.2.2.2.	*Propionibacteriales*	1	0.23%	0.43%	0.84%	0.60%	0%	0%	0.05%	0.06%	0.08%	0.13%	0.07%	0.06%	0.45%
4	0.2.2.2.	*Pseudonocardiales*	1	0.14%	1.40%	0.26%	0.05%	0%	0%	0%	0%	0%	0%	0%	0%	0%
4	0.2.2.2.	*Streptomycetales*	1	0.18%	0.59%	0.41%	0.50%	0%	0%	0.14%	0.13%	0.08%	0%	0.25%	0%	0%
4	0.2.2.2.	*Streptosporangiales*	2	0.05%	0.04%	0.45%	0.09%	0%	0%	0%	0%	0%	0%	0%	0%	0%
4	0.2.2.2.	*unclassified*	1	0.03%	0.08%	0.07%	0.05%	0%	0%	0%	0%	0%	0%	0%	0%	0.15%
4	0.2.2.3.	*Solirubrobacterales*	2	0.10%	0.35%	0.41%	0.46%	0%	0%	0%	0%	0%	0%	0%	0%	0%
4	0.2.2.4.	*unclassified*	1	0.02%	0.07%	0.08%	0.05%	0%	0%	0%	0%	0%	0%	0%	0%	0%
4	0.2.3.1.	*Armatimonadales*	1	0.01%	0%	0.03%	0.09%	0%	0%	0%	0%	0%	0%	0%	0%	0%
4	0.2.3.2.	*unclassified*	1	0.08%	0.18%	0.43%	0.14%	0%	0%	0%	0%	0%	0.07%	0.05%	0.06%	0%
4	0.2.4.1.	*Bacteroidales*	2	0.05%	0.04%	0%	0.09%	0.15%	0%	0%	0%	0.17%	0%	0%	0.19%	0%
4	0.2.4.2.	*Cytophagales*	2	0.51%	1.68%	1.98%	1.56%	0.30%	0%	0.23%	0%	0%	0.20%	0.17%	0%	0%
4	0.2.4.3.	*Flavobacteriales*	2	3.37%	13.28%	10.34%	9.40%	4.67%	0%	0.27%	0.25%	0.08%	0.86%	0.62%	0.38%	0.30%
4	0.2.4.4.	*Sphingobacteriales*	6	2.12%	7.05%	8.40%	6.24%	1.05%	0%	0.18%	0.06%	0.17%	0.53%	0.42%	0.69%	0.60%
4	0.2.4.5.	*unclassified*	1	0.04%	0.25%	0.18%	0.05%	0%	0%	0%	0%	0%	0%	0%	0%	0%
4	0.2.5.1.	*unclassified*	1	0.01%	0%	0.05%	0.05%	0%	0%	0%	0%	0%	0%	0%	0%	0%
4	0.2.6.1.	*unclassified*	1	0.03%	0.22%	0.12%	0%	0%	0%	0%	0%	0%	0%	0.07%	0%	0%
4	0.2.7.1.	*Unknown_Order*	1	0.02%	0.01%	0%	0%	0%	0%	0%	0.06%	0.08%	0.07%	0.02%	0%	0%
4	0.2.7.2.	*unclassified*	1	0.10%	0.29%	0.18%	0.41%	0%	0%	0%	0.13%	0.08%	0%	0.07%	0%	0%
4	0.2.8.1.	*Chlamydiales*	1	0.02%	0.14%	0.08%	0.05%	0%	0%	0%	0%	0%	0%	0%	0%	0%
4	0.2.9.1.	*Chlorobiales*	1	0%	0.04%	0.02%	0%	0%	0%	0%	0%	0%	0%	0%	0%	0%
4	0.2.9.2.	*unclassified*	1	0.03%	0.01%	0.03%	0.28%	0%	0%	0%	0%	0%	0%	0%	0%	0%
4	0.2.10.1.	*Anaerolineales*	1	0.01%	0%	0.02%	0.05%	0%	0%	0%	0%	0%	0%	0.02%	0%	0%
4	0.2.10.2.	*Caldilineales*	1	0.01%	0.06%	0.05%	0.05%	0%	0%	0%	0%	0%	0%	0%	0%	0%
4	0.2.10.3.	*Chloroflexales*	2	0.07%	0.21%	0.15%	0.23%	0%	0%	0%	0%	0.08%	0%	0%	0%	0.15%
4	0.2.10.4.	*unclassified*	1	0.01%	0.03%	0.03%	0%	0%	0%	0%	0%	0%	0%	0%	0%	0%
4	0.2.10.5.	*Ktedonobacterales*	1	0.01%	0%	0%	0%	0%	0%	0%	0%	0%	0%	0.12%	0%	0%
4	0.2.10.6.	*unclassified*	1	0.03%	0.06%	0.15%	0.09%	0%	0%	0%	0%	0%	0.07%	0%	0%	0%
4	0.2.10.7.	*AKYG1722*	1	0.02%	0.11%	0.10%	0%	0%	0%	0%	0%	0%	0%	0%	0%	0%
4	0.2.10.7.	*JG30-KF-CM45*	1	0.36%	0.63%	1.27%	1.51%	0%	0%	0%	0%	0%	0.07%	0.07%	0%	0.74%
4	0.2.10.8.	*unclassified*	1	0.03%	0.15%	0.20%	0.05%	0%	0%	0%	0%	0%	0%	0%	0%	0%
4	0.2.11.1.	*Bacillales*	4	1.72%	1.64%	0.69%	1.01%	0.15%	0%	4.53%	0.06%	0.25%	0.47%	11.24%	0.50%	0.15%
4	0.2.11.1.	*Lactobacillales*	3	0.47%	0.01%	0.02%	0%	0%	0%	1.46%	0.06%	0%	0.13%	3.49%	0.13%	0.30%
4	0.2.11.2.	*Clostridiales*	2	0.03%	0.08%	0.05%	0.05%	0%	0%	0.09%	0%	0%	0%	0.05%	0%	0%
4	0.2.11.3.	*unclassified*	1	0.02%	0%	0%	0%	0%	0%	0.05%	0.13%	0%	0%	0.02%	0%	0%
4	0.2.12.1.	*Fusobacteriales*	1	0.05%	0.06%	0%	0%	0%	0%	0.09%	0.13%	0.17%	0%	0%	0%	0.15%
4	0.2.13.1.	*Gemmatimonadales*	1	0%	0.01%	0.03%	0%	0%	0%	0%	0%	0%	0%	0%	0%	0%
4	0.2.14.1.	*Nitrospirales*	1	0%	0%	0.05%	0%	0%	0%	0%	0%	0%	0%	0%	0%	0%
4	0.2.15.1.	*WD2101_soil_group*	1	0.05%	0.03%	0.31%	0.23%	0%	0%	0%	0%	0%	0%	0%	0%	0%
4	0.2.15.2.	*Planctomycetales*	1	0.39%	0.85%	1.85%	1.33%	0%	0%	0.05%	0.06%	0.08%	0.13%	0.07%	0%	0.30%
4	0.2.15.3.	*unclassified*	1	0.01%	0.11%	0.05%	0%	0%	0%	0%	0%	0%	0%	0%	0%	0%
4	0.2.16.1.	*Caulobacterales*	2	0.26%	0.77%	0.46%	1.28%	0.30%	0%	0.05%	0.06%	0.08%	0%	0.05%	0%	0%
4	0.2.16.1.	*DB1-14*	1	0.03%	0.03%	0.05%	0.23%	0%	0%	0%	0%	0%	0%	0.05%	0%	0%
4	0.2.16.1.	*Rhizobiales*	10	4.90%	8.84%	11.05%	10.82%	9.04%	1.10%	2.10%	1.38%	3.24%	2.59%	1.97%	2.44%	4.17%
4	0.2.16.1.	*Rhodobacterales*	1	0.09%	0.01%	0.05%	0.87%	0%	0%	0.05%	0%	0%	0.07%	0%	0%	0%
4	0.2.16.1.	*Rhodospirillales*	6	0.22%	0.66%	1.24%	0.69%	0%	0%	0.05%	0%	0%	0.07%	0%	0%	0%
4	0.2.16.1.	*Sphingomonadales*	3	0.59%	1.21%	1.29%	2.38%	0%	0%	0%	0.13%	0.08%	0.13%	0.02%	0.38%	1.49%
4	0.2.16.1.	*unclassified*	1	0.34%	0.10%	0.31%	0.69%	0%	0%	0%	0.19%	0.50%	2.33%	0%	0%	0%
4	0.2.16.2.	*Burkholderiales*	4	2.30%	5.65%	7.49%	5.82%	4.82%	0%	0.23%	0.19%	0.50%	2.13%	0.32%	0.25%	0.15%
4	0.2.16.2.	*Methylophilales*	1	0.49%	0.81%	0.63%	0.83%	3.16%	0%	0%	0.06%	0%	0.13%	0%	0.13%	0.15%
4	0.2.16.2.	*Nitrosomonadales*	1	0.02%	0.01%	0.08%	0.09%	0%	0%	0%	0%	0%	0%	0%	0%	0%
4	0.2.16.2.	*Rhodocyclales*	1	0.12%	0.45%	0.28%	0.32%	0.30%	0%	0%	0%	0%	0%	0%	0.06%	0%
4	0.2.16.2.	*TRA3-20*	1	0.02%	0.03%	0.12%	0%	0%	0%	0.05%	0%	0%	0%	0%	0%	0%
4	0.2.16.2.	*unclassified*	1	0.02%	0.10%	0.10%	0.05%	0%	0%	0%	0%	0%	0%	0%	0%	0%
4	0.2.16.3.	*Bdellovibrionales*	1	0.02%	0.10%	0.08%	0.05%	0%	0%	0%	0%	0%	0%	0.02%	0%	0%
4	0.2.16.3.	*Myxococcales*	3	0.25%	0.71%	1.37%	0.87%	0%	0%	0.05%	0%	0%	0%	0.05%	0%	0%
4	0.2.16.3.	*unclassified*	1	0.01%	0.04%	0.13%	0%	0%	0%	0%	0%	0%	0%	0%	0%	0%
4	0.2.16.4.	*Enterobacteriales*	1	2.38%	13.00%	6.14%	3.58%	1.36%	0%	0.46%	0.13%	0.58%	1.13%	0.45%	0.25%	1.49%
4	0.2.16.4.	*NKB5*	1	0%	0%	0.03%	0%	0%	0%	0%	0%	0%	0%	0%	0%	0%
4	0.2.16.4.	*Oceanospirillales*	2	1.53%	0.77%	0.74%	0.09%	1.96%	1.10%	1.60%	1.19%	2.16%	3.73%	1.67%	2.01%	1.34%
4	0.2.16.4.	*Pseudomonadales*	2	3.07%	8.27%	5.59%	9.49%	1.36%	0%	1.05%	0.38%	0.75%	1.60%	1.67%	2.69%	4.02%
4	0.2.16.4.	*Xanthomonadales*	2	0.88%	2.26%	2.98%	3.48%	0.60%	0%	0.14%	0.06%	0.17%	0.53%	0.17%	0.13%	0%
4	0.2.16.4.	*unclassified*	1	0.15%	0.31%	0.71%	0.64%	0%	0%	0.05%	0%	0%	0.07%	0%	0%	0%
4	0.2.16.5.	*unclassified*	1	0.06%	0.11%	0.41%	0.18%	0%	0%	0%	0%	0%	0.07%	0%	0%	0%
4	0.2.17.1.	*unclassified*	1	0.01%	0%	0%	0%	0%	0%	0%	0%	0%	0.13%	0%	0%	0%
4	0.2.17.2.	*unclassified*	1	0.01%	0%	0.02%	0.05%	0%	0%	0%	0%	0%	0%	0%	0%	0%
4	0.2.17.3.	*Chthoniobacterales*	3	0.07%	0.21%	0.41%	0.14%	0%	0%	0.05%	0%	0%	0%	0%	0%	0%
4	0.2.17.4.	*Verrucomicrobiales*	1	0.26%	0.74%	1.60%	0.55%	0%	0%	0%	0%	0%	0%	0.12%	0%	0.15%
4	0.2.17.5.	*unclassified*	1	0.02%	0.07%	0.18%	0%	0%	0%	0%	0%	0%	0%	0%	0%	0%
4	0.2.18.1.	*unclassified*	1	61.77%	17.18%	14.39%	20.77%	68.83%	97.25%	80.29%	91.33%	64.98%	55.36%	69.14%	86.40%	75.30%
4	0.3.1.1.	*unclassified*	1	6.16%	0.99%	4.12%	0.32%	0%	0.55%	5.67%	3.20%	24.56%	25.02%	6.53%	2.32%	0.60%
5	0.1.1.1.	*Halobacteriaceae*	1	0.02%	0.03%	0%	0%	0%	0%	0%	0%	0%	0.07%	0%	0%	0.15%
5	0.2.1.1.	*Unknown_Family*	1	0.05%	0.14%	0.17%	0.23%	0%	0%	0%	0%	0%	0.07%	0.07%	0%	0%
5	0.2.1.1.	*Unknown_Family*	1	0.09%	0.20%	0.46%	0.28%	0%	0%	0.09%	0%	0%	0%	0%	0%	0%
5	0.2.1.1.	*unclassified*	1	0.03%	0.21%	0.07%	0.05%	0%	0%	0%	0%	0%	0%	0%	0%	0%
5	0.2.1.2.	*unclassified*	1	0.04%	0.10%	0.31%	0.09%	0%	0%	0%	0%	0%	0%	0%	0%	0%
5	0.2.2.1.	*Acidimicrobiaceae*	2	0.09%	0.21%	0.40%	0.23%	0%	0%	0%	0%	0%	0%	0.05%	0%	0.15%
5	0.2.2.1.	*unclassified*	1	0.04%	0.14%	0.20%	0.14%	0%	0%	0%	0%	0%	0%	0%	0%	0%
5	0.2.2.2.	*Corynebacteriaceae*	1	0.08%	0.03%	0.02%	0.09%	0.15%	0%	0.14%	0.13%	0.08%	0.13%	0.10%	0.06%	0%
5	0.2.2.2.	*Mycobacteriaceae*	1	0.45%	0.60%	1.07%	0.87%	0.15%	0%	0%	0%	0%	0.33%	0%	0.19%	2.23%
5	0.2.2.2.	*Nocardiaceae*	3	0.47%	1.44%	1.09%	1.74%	0.15%	0%	0.14%	0%	0.17%	0.33%	0.10%	0.06%	0.45%
5	0.2.2.2.	*Nakamurellaceae*	1	0.01%	0.01%	0.02%	0.09%	0%	0%	0%	0%	0%	0%	0%	0%	0%
5	0.2.2.2.	*Kineosporiaceae*	1	0.04%	0.07%	0.10%	0.32%	0%	0%	0%	0%	0%	0%	0%	0%	0%
5	0.2.2.2.	*Cellulomonadaceae*	1	0.03%	0.10%	0%	0%	0%	0%	0%	0%	0%	0.07%	0%	0%	0.15%

5	0.2.2.2.4	*Dermabacteraceae*	1	0.02%	0.06%	0%	0%	0%	0%	0.09%	0%	0%	0%	0%	0.06%	0%
5	0.2.2.2.4	*Intrasporangiaceae*	1	0.05%	0.03%	0.10%	0.05%	0%	0%	0%	0%	0.08%	0%	0%	0.06%	0.30%
5	0.2.2.2.4	*Microbacteriaceae*	3	0.59%	1.01%	1.89%	1.01%	1.20%	0%	0.05%	0%	0.17%	0.53%	0.05%	0.19%	1.04%
5	0.2.2.2.4	*Micrococcaceae*	2	0.91%	0.87%	1.04%	3.16%	0.30%	0%	0.50%	0.44%	0.58%	0.53%	0.55%	0.25%	2.68%
5	0.2.2.2.4	*Promicromonosporaceae*	1	0.09%	0.31%	0.17%	0.46%	0%	0%	0%	0%	0%	0%	0%	0%	0.15%
5	0.2.2.2.4	*unclassified*	1	0.01%	0.03%	0.12%	0%	0%	0%	0%	0%	0%	0%	0%	0%	0%
5	0.2.2.2.5	*Micromonosporaceae*	4	0.42%	0.74%	1.32%	2.20%	0%	0%	0%	0%	0%	0.13%	0.02%	0.06%	0.60%
5	0.2.2.2.6	*Nocardioidaceae*	3	0.23%	0.43%	0.84%	0.60%	0%	0%	0.05%	0.06%	0.08%	0.13%	0.07%	0.06%	0.45%
5	0.2.2.2.7	*Pseudonocardiaceae*	2	0.14%	1.40%	0.26%	0.05%	0%	0%	0%	0%	0%	0%	0%	0%	0%
5	0.2.2.2.8	*Streptomycetaceae*	1	0.18%	0.59%	0.41%	0.50%	0%	0%	0.14%	0.13%	0.08%	0%	0.25%	0%	0%
5	0.2.2.2.9	*Streptosporangiaceae*	1	0.01%	0%	0.05%	0.09%	0%	0%	0%	0%	0%	0%	0%	0%	0%
5	0.2.2.2.9	*Thermomonosporaceae*	1	0.04%	0.04%	0.40%	0%	0%	0%	0%	0%	0%	0%	0%	0%	0%
5	0.2.2.2.	*unclassified*	1	0.03%	0.08%	0.07%	0.05%	0%	0%	0%	0%	0%	0%	0%	0%	0.15%
5	0.2.2.3.	*Conexibacteraceae*	1	0.01%	0.06%	0.07%	0.05%	0%	0%	0%	0%	0%	0%	0%	0%	0%
5	0.2.2.3.	*unclassified*	1	0.09%	0.29%	0.35%	0.41%	0%	0%	0%	0%	0%	0%	0%	0%	0%
5	0.2.2.4.	*unclassified*	1	0.02%	0.07%	0.08%	0.05%	0%	0%	0%	0%	0%	0%	0%	0%	0%
5	0.2.3.1.	*Armatimonadaceae*	1	0.01%	0%	0.03%	0.09%	0%	0%	0%	0%	0%	0%	0%	0%	0%
5	0.2.3.2.	*unclassified*	1	0.08%	0.18%	0.43%	0.14%	0%	0%	0%	0%	0%	0.07%	0.05%	0.06%	0%
5	0.2.4.1.	*Bacteroidaceae*	1	0.05%	0.04%	0%	0%	0.15%	0%	0%	0%	0.17%	0%	0%	0.19%	0%
5	0.2.4.1.	*Prevotellaceae*	1	0.01%	0%	0%	0.09%	0%	0%	0%	0%	0%	0%	0%	0%	0%
5	0.2.4.2.	*Cytophagaceae*	7	0.49%	1.63%	1.84%	1.51%	0.30%	0%	0.23%	0%	0%	0.20%	0.17%	0%	0%
5	0.2.4.2.	*unclassified*	1	0.02%	0.06%	0.15%	0.05%	0%	0%	0%	0%	0%	0%	0%	0%	0%
5	0.2.4.3.	*Cryomorphaceae*	1	0.03%	0.15%	0.15%	0.05%	0%	0%	0%	0%	0%	0%	0%	0%	0%
5	0.2.4.3.	*Flavobacteriaceae*	4	3.34%	13.13%	10.19%	9.35%	4.67%	0%	0.27%	0.25%	0.08%	0.86%	0.62%	0.38%	0.30%
5	0.2.4.4.	*Chitinophagaceae*	6	0.74%	3.36%	2.55%	2.25%	0%	0%	0.14%	0.06%	0%	0.07%	0.07%	0.38%	0%
5	0.2.4.4.	*KD3-93*	1	0.01%	0.03%	0%	0.14%	0%	0%	0%	0%	0%	0%	0%	0%	0%
5	0.2.4.4.	*NS11-12_marine_group*	1	0.02%	0.04%	0%	0.18%	0%	0%	0%	0%	0%	0%	0%	0%	0%
5	0.2.4.4.	*Sphingobacteriaceae*	5	1.31%	3.43%	5.74%	3.58%	1.05%	0%	0.05%	0%	0.17%	0.47%	0.35%	0.31%	0.60%
5	0.2.4.4.	*env.OPS_17*	1	0.02%	0.10%	0.07%	0.05%	0%	0%	0%	0%	0%	0%	0%	0%	0%
5	0.2.4.4.	*unclassified*	1	0.01%	0.08%	0.05%	0.05%	0%	0%	0%	0%	0%	0%	0%	0%	0%
5	0.2.4.5.	*unclassified*	1	0.04%	0.25%	0.18%	0.05%	0%	0%	0%	0%	0%	0%	0%	0%	0%
5	0.2.5.1.	*unclassified*	1	0.01%	0%	0.05%	0.05%	0%	0%	0%	0%	0%	0%	0%	0%	0%
5	0.2.6.1.	*unclassified*	1	0.03%	0.22%	0.12%	0%	0%	0%	0%	0%	0%	0%	0.07%	0%	0%
5	0.2.7.1.	*Unknown_Family*	1	0.02%	0.01%	0%	0%	0%	0%	0%	0.06%	0.08%	0.07%	0.02%	0%	0%
5	0.2.7.2.	*unclassified*	1	0.10%	0.29%	0.18%	0.41%	0%	0%	0%	0.13%	0.08%	0%	0.07%	0%	0%
5	0.2.8.1.	*Parachlamydiaceae*	2	0.02%	0.14%	0.08%	0.05%	0%	0%	0%	0%	0%	0%	0%	0%	0%
5	0.2.9.1.	*SJA-28*	1	0%	0.04%	0.02%	0%	0%	0%	0%	0%	0%	0%	0%	0%	0%
5	0.2.9.2.	*unclassified*	1	0.03%	0.01%	0.03%	0.28%	0%	0%	0%	0%	0%	0%	0%	0%	0%
5	0.2.10.1.	*Anaerolineaceae*	1	0.01%	0%	0.02%	0.05%	0%	0%	0%	0%	0%	0%	0.02%	0%	0%
5	0.2.10.2.	*Caldilineaceae*	2	0.01%	0.06%	0.05%	0.05%	0%	0%	0%	0%	0%	0%	0%	0%	0%
5	0.2.10.3.	*Chloroflexales_Incertae_*	1	0.02%	0.11%	0.05%	0.09%	0%	0%	0%	0%	0%	0%	0%	0%	0%
5	0.2.10.3.	*Roseiflexaceae*	1	0.05%	0.10%	0.10%	0.14%	0%	0%	0%	0%	0.08%	0%	0%	0%	0.15%
5	0.2.10.4.	*unclassified*	1	0.01%	0.03%	0.03%	0%	0%	0%	0%	0%	0%	0%	0%	0%	0%
5	0.2.10.5.	*Thermosporotrichaceae*	1	0.01%	0%	0%	0%	0%	0%	0%	0%	0%	0%	0.12%	0%	0%
5	0.2.10.6.	*unclassified*	1	0.03%	0.06%	0.15%	0.09%	0%	0%	0%	0%	0%	0.07%	0%	0%	0%
5	0.2.10.7.	*unclassified*	1	0.02%	0.11%	0.10%	0%	0%	0%	0%	0%	0%	0%	0%	0%	0%
5	0.2.10.7.	*unclassified*	1	0.36%	0.63%	1.27%	1.51%	0%	0%	0%	0%	0%	0.07%	0.07%	0%	0.74%
5	0.2.10.8.	*unclassified*	1	0.03%	0.15%	0.20%	0.05%	0%	0%	0%	0%	0%	0%	0%	0%	0%
5	0.2.11.1.	*Bacillaceae*	1	1.34%	0.13%	0%	0%	0.15%	0%	4.30%	0.06%	0.08%	0.27%	10.82%	0.25%	0%
5	0.2.11.1.	*Paenibacillaceae*	2	0.26%	1.40%	0.66%	1.01%	0%	0%	0%	0%	0%	0.07%	0%	0%	0%
5	0.2.11.1.	*Planococcaceae*	1	0.08%	0.06%	0.02%	0%	0%	0%	0.09%	0%	0%	0.07%	0.35%	0.19%	0.15%
5	0.2.11.1.	*Staphylococcaceae*	1	0.05%	0.06%	0.02%	0%	0%	0%	0.14%	0%	0.17%	0.07%	0.07%	0.06%	0%
5	0.2.11.1.	*Carnobacteriaceae*	1	0.06%	0%	0.02%	0%	0%	0%	0.05%	0%	0%	0.07%	0.17%	0.06%	0.30%
5	0.2.11.1.	*Enterococcaceae*	1	0.39%	0%	0%	0%	0%	0%	1.42%	0%	0%	0%	3.32%	0%	0%
5	0.2.11.1.	*Streptococcaceae*	1	0.02%	0.01%	0%	0%	0%	0%	0%	0.06%	0%	0.07%	0%	0.06%	0%
5	0.2.11.2.	*Lachnospiraceae*	1	0.01%	0.06%	0.05%	0.05%	0%	0%	0%	0%	0%	0%	0%	0%	0%
5	0.2.11.2.	*Ruminococcaceae*	2	0.01%	0.03%	0%	0%	0%	0%	0.09%	0%	0%	0%	0.05%	0%	0%
5	0.2.11.3.	*unclassified*	1	0.02%	0%	0%	0%	0%	0%	0.05%	0.13%	0%	0%	0.02%	0%	0%
5	0.2.12.1.	*Fusobacteriaceae*	1	0.05%	0.06%	0%	0%	0%	0%	0.09%	0.13%	0.17%	0%	0%	0%	0.15%
5	0.2.13.1.	*Gemmatimonadaceae*	1	0%	0.01%	0.03%	0%	0%	0%	0%	0%	0%	0%	0%	0%	0%
5	0.2.14.1.	*Nitrospiraceae*	1	0%	0%	0.05%	0%	0%	0%	0%	0%	0%	0%	0%	0%	0%
5	0.2.15.1.	*unclassified*	1	0.05%	0.03%	0.31%	0.23%	0%	0%	0%	0%	0%	0%	0%	0%	0%
5	0.2.15.2.	*Planctomycetaceae*	5	0.39%	0.85%	1.85%	1.33%	0%	0%	0.05%	0.06%	0.08%	0.13%	0.07%	0%	0.30%
5	0.2.15.3.	*unclassified*	1	0.01%	0.11%	0.05%	0%	0%	0%	0%	0%	0%	0%	0%	0%	0%
5	0.2.16.1.	*Caulobacteraceae*	4	0.19%	0.67%	0.28%	0.83%	0.30%	0%	0.05%	0.06%	0.08%	0%	0.05%	0%	0%
5	0.2.16.1.	*Hyphomonadaceae*	1	0.06%	0.10%	0.18%	0.46%	0%	0%	0%	0%	0%	0%	0%	0%	0%
5	0.2.16.1.	*unclassified*	1	0.03%	0.03%	0.05%	0.23%	0%	0%	0%	0%	0%	0%	0.05%	0%	0%
5	0.2.16.1.	*Beijerinckiaceae*	1	0.02%	0.03%	0.02%	0.18%	0%	0%	0%	0%	0%	0%	0%	0%	0%
5	0.2.16.1.	*Bradyrhizobiaceae*	4	0.27%	0.71%	0.88%	1.24%	0%	0%	0.09%	0.06%	0%	0.13%	0.07%	0%	0%
5	0.2.16.1.	*Brucellaceae*	1	0.24%	0.34%	0.61%	0.60%	0.75%	0%	0%	0.06%	0.17%	0.13%	0.07%	0%	0.15%
5	0.2.16.1.	*Hyphomicrobiaceae*	5	1.51%	2.10%	2.75%	2.52%	3.31%	0.55%	1.05%	0.38%	1.66%	1.06%	0.62%	1.50%	0.60%
5	0.2.16.1.	*Methylobacteriaceae*	2	0.07%	0.11%	0.07%	0.32%	0%	0%	0%	0%	0%	0.13%	0.05%	0%	0.15%
5	0.2.16.1.	*Phyllobacteriaceae*	2	0.89%	0.46%	0.98%	1.10%	1.66%	0.55%	0.91%	0.63%	1.08%	0.86%	0.65%	0.56%	1.19%
5	0.2.16.1.	*Rhizobiaceae*	3	1.33%	3.18%	3.21%	3.44%	3.31%	0%	0.05%	0.13%	0.17%	0.27%	0.37%	0.25%	1.64%
5	0.2.16.1.	*Rhizobiales_Incertae_Se*	3	0.08%	0.20%	0.45%	0.14%	0%	0%	0%	0.06%	0%	0%	0%	0.06%	0%
5	0.2.16.1.	*Xanthobacteraceae*	2	0.04%	0.21%	0.05%	0.05%	0%	0%	0%	0%	0%	0%	0%	0%	0.15%
5	0.2.16.1.	*unclassified*	1	0.46%	1.50%	2.05%	1.24%	0%	0%	0%	0.06%	0.17%	0%	0.12%	0.06%	0.30%
5	0.2.16.1.	*Rhodobacteraceae*	2	0.09%	0.01%	0.05%	0.87%	0%	0%	0.05%	0%	0%	0.07%	0%	0%	0%
5	0.2.16.1.	*AKYH478*	1	0.03%	0.06%	0.25%	0.05%	0%	0%	0.05%	0%	0%	0%	0%	0%	0%
5	0.2.16.1.	*Acetobacteraceae*	1	0.01%	0.03%	0.10%	0.05%	0%	0%	0%	0%	0%	0%	0%	0%	0%
5	0.2.16.1.	*MNC12*	1	0%	0.01%	0.02%	0%	0%	0%	0%	0%	0%	0%	0%	0%	0%
5	0.2.16.1.	*Rhodospirillaceae*	3	0.08%	0.31%	0.38%	0.23%	0%	0%	0%	0%	0%	0%	0%	0%	0%
5	0.2.16.1.	*Rhodospirillales_Incert*	2	0.05%	0.14%	0.23%	0.28%	0%	0%	0%	0%	0%	0%	0%	0%	0%
5	0.2.16.1.	*unclassified*	1	0.04%	0.11%	0.26%	0.09%	0%	0%	0%	0%	0%	0.07%	0%	0%	0%
5	0.2.16.1.	*Erythrobacteraceae*	1	0.04%	0.08%	0.08%	0.28%	0%	0%	0%	0%	0%	0%	0%	0%	0%
5	0.2.16.1.	*Sphingomonadaceae*	4	0.50%	0.92%	1.14%	1.88%	0%	0%	0%	0.06%	0.08%	0.13%	0.02%	0.25%	1.49%
5	0.2.16.1.	*unclassified*	1	0.06%	0.20%	0.07%	0.23%	0%	0%	0%	0.06%	0%	0%	0%	0.13%	0%
5	0.2.16.1.	*unclassified*	1	0.34%	0.10%	0.31%	0.69%	0%	0%	0%	0.19%	0.50%	2.33%	0%	0%	0%
5	0.2.16.2.	*Alcaligenaceae*	1	0.35%	0.97%	1.44%	1.10%	0.30%	0%	0.05%	0%	0.08%	0.13%	0.02%	0.06%	0%

5	0.2.16.2.	*Burkholderiaceae*	2	0.07%	0.03%	0.05%	0.09%	0%	0%	0.05%	0.06%	0%	0.47%	0.05%	0.06%	0%
5	0.2.16.2.	*Comamonadaceae*	10	1.14%	3.53%	4.37%	3.03%	1.05%	0%	0.14%	0.13%	0.25%	0.93%	0.10%	0.13%	0%
5	0.2.16.2.	*Oxalobacteraceae*	4	0.74%	1.12%	1.64%	1.60%	3.46%	0%	0%	0%	0.17%	0.60%	0.15%	0%	0.15%
5	0.2.16.2.	*Methylophilaceae*	4	0.49%	0.81%	0.63%	0.83%	3.16%	0%	0%	0.06%	0%	0.13%	0%	0.13%	0.15%
5	0.2.16.2.	*Nitrosomonadaceae*	1	0.02%	0.01%	0.08%	0.09%	0%	0%	0%	0%	0%	0%	0%	0%	0%
5	0.2.16.2.	*Rhodocyclaceae*	1	0.12%	0.45%	0.28%	0.32%	0.30%	0%	0%	0%	0%	0%	0%	0.06%	0%
5	0.2.16.2.	*unclassified*	1	0.02%	0.03%	0.12%	0%	0%	0%	0.05%	0%	0%	0%	0%	0%	0%
5	0.2.16.2.	*unclassified*	1	0.02%	0.10%	0.10%	0.05%	0%	0%	0%	0%	0%	0%	0%	0%	0%
5	0.2.16.3.	*Bdellovibrionaceae*	2	0.02%	0.10%	0.08%	0.05%	0%	0%	0%	0%	0%	0%	0.02%	0%	0%
5	0.2.16.3.	*Nannocystaceae*	1	0.04%	0.08%	0.30%	0.05%	0%	0%	0%	0%	0%	0%	0%	0%	0%
5	0.2.16.3.	*Polyangiaceae*	3	0.10%	0.38%	0.40%	0.37%	0%	0%	0%	0%	0%	0%	0%	0%	0%
5	0.2.16.3.	*unclassified*	1	0.12%	0.25%	0.68%	0.46%	0%	0%	0.05%	0%	0%	0%	0.05%	0%	0%
5	0.2.16.3.	*unclassified*	1	0.01%	0.04%	0.13%	0%	0%	0%	0%	0%	0%	0%	0%	0%	0%
5	0.2.16.4.	*Enterobacteriaceae*	5	2.38%	13.00%	6.14%	3.58%	1.36%	0%	0.46%	0.13%	0.58%	1.13%	0.45%	0.25%	1.49%
5	0.2.16.4.	*unclassified*	1	0%	0%	0.03%	0%	0%	0%	0%	0%	0%	0%	0%	0%	0%
5	0.2.16.4.	*Halomonadaceae*	1	1.52%	0.76%	0.66%	0.09%	1.96%	1.10%	1.60%	1.19%	2.16%	3.73%	1.67%	2.01%	1.34%
5	0.2.16.4.	*Oceanospirillaceae*	1	0.01%	0.01%	0.08%	0%	0%	0%	0%	0%	0%	0%	0%	0%	0%
5	0.2.16.4.	*Moraxellaceae*	2	0.33%	0.32%	0.20%	0.05%	0%	0%	0.50%	0.31%	0.17%	0.07%	0.67%	0.38%	1.34%
5	0.2.16.4.	*Pseudomonadaceae*	3	2.74%	7.95%	5.39%	9.45%	1.36%	0%	0.55%	0.06%	0.58%	1.53%	1.00%	2.32%	2.68%
5	0.2.16.4.	*Xanthomonadaceae*	9	0.84%	1.99%	2.84%	3.48%	0.60%	0%	0.14%	0.06%	0.17%	0.53%	0.17%	0.13%	0%
5	0.2.16.4.	*Xanthomonadales_Incerta*	1	0.03%	0.27%	0.13%	0%	0%	0%	0%	0%	0%	0%	0%	0%	0%
5	0.2.16.4.	*unclassified*	1	0.15%	0.31%	0.71%	0.64%	0%	0%	0.05%	0%	0%	0.07%	0%	0%	0%
5	0.2.16.5.	*unclassified*	1	0.06%	0.11%	0.41%	0.18%	0%	0%	0%	0%	0%	0.07%	0%	0%	0%
5	0.2.17.1.	*unclassified*	1	0.01%	0%	0%	0%	0%	0%	0%	0%	0%	0.13%	0%	0%	0%
5	0.2.17.2.	*unclassified*	1	0.01%	0%	0.02%	0.05%	0%	0%	0%	0%	0%	0%	0%	0%	0%
5	0.2.17.3.	*DA101_soil_group*	1	0.04%	0.17%	0.18%	0.14%	0%	0%	0%	0%	0%	0%	0%	0%	0%
5	0.2.17.3.	*Xiphinematobacteraceae*	1	0.02%	0.04%	0.17%	0%	0%	0%	0.05%	0%	0%	0%	0%	0%	0%
5	0.2.17.3.	*unclassified*	1	0.01%	0%	0.07%	0%	0%	0%	0%	0%	0%	0%	0%	0%	0%
5	0.2.17.4.	*Verrucomicrobiaceae*	5	0.26%	0.74%	1.60%	0.55%	0%	0%	0%	0%	0%	0%	0.12%	0%	0.15%
5	0.2.17.5.	*unclassified*	1	0.02%	0.07%	0.18%	0%	0%	0%	0%	0%	0%	0%	0%	0%	0%
5	0.2.18.1.	*unclassified*	1	61.77%	17.18%	14.39%	20.77%	68.83%	97.25%	80.29%	91.33%	64.98%	55.36%	69.14%	86.40%	75.30%
5	0.3.1.1.	*unclassified*	1	6.16%	0.99%	4.12%	0.32%	0%	0.55%	5.67%	3.20%	24.56%	25.02%	6.53%	2.32%	0.60%
6	0.1.1.1.	*Halorubrum*	0	0.02%	0.03%	0%	0%	0%	0%	0%	0%	0%	0.07%	0%	0%	0.15%
6	0.2.1.1.	*Bryobacter*	0	0.05%	0.14%	0.17%	0.23%	0%	0%	0%	0%	0%	0.07%	0%	0%	0%
6	0.2.1.1.	*Blastocatella*	0	0.09%	0.20%	0.46%	0.28%	0%	0%	0.09%	0%	0%	0%	0%	0%	0%
6	0.2.1.1.	*unclassified*	0	0.03%	0.21%	0.07%	0.05%	0%	0%	0%	0%	0%	0%	0%	0%	0%
6	0.2.1.2.	*unclassified*	0	0.04%	0.10%	0.31%	0.09%	0%	0%	0%	0%	0%	0%	0%	0%	0%
6	0.2.2.1.	*Ilumatobacter*	0	0.07%	0.13%	0.30%	0.18%	0%	0%	0%	0%	0%	0%	0.05%	0%	0.15%
6	0.2.2.1.	*unclassified*	0	0.02%	0.08%	0.10%	0.05%	0%	0%	0%	0%	0%	0%	0%	0%	0%
6	0.2.2.1.	*unclassified*	0	0.04%	0.14%	0.20%	0.14%	0%	0%	0%	0%	0%	0%	0%	0%	0%
6	0.2.2.2.	*Corynebacterium*	0	0.08%	0.03%	0.02%	0.09%	0.15%	0%	0.14%	0.13%	0.08%	0.13%	0.10%	0.06%	0%
6	0.2.2.2.	*Mycobacterium*	0	0.45%	0.60%	1.07%	0.87%	0.15%	0%	0%	0%	0%	0.33%	0%	0.19%	2.23%
6	0.2.2.2.	*Gordonia*	0	0.05%	0.04%	0.02%	0.09%	0.15%	0%	0%	0%	0.17%	0%	0.02%	0%	0.15%
6	0.2.2.2.	*Nocardia*	0	0.01%	0.01%	0%	0.09%	0%	0%	0%	0%	0%	0%	0.02%	0%	0%
6	0.2.2.2.	*Rhodococcus*	0	0.41%	1.39%	1.07%	1.56%	0%	0%	0.14%	0%	0%	0.33%	0.05%	0.06%	0.30%
6	0.2.2.2.	*Nakamurella*	0	0.01%	0.01%	0.02%	0.09%	0%	0%	0%	0%	0%	0%	0%	0%	0%
6	0.2.2.2.	*Kineosporia*	0	0.04%	0.07%	0.10%	0.32%	0%	0%	0%	0%	0%	0%	0%	0%	0%
6	0.2.2.2.	*Cellulomonas*	0	0.03%	0.10%	0%	0%	0%	0%	0%	0%	0%	0.07%	0%	0%	0.15%
6	0.2.2.2.	*Brachybacterium*	0	0.02%	0.06%	0%	0%	0%	0%	0.09%	0%	0%	0%	0%	0.06%	0%
6	0.2.2.2.	*unclassified*	0	0.05%	0.03%	0.10%	0.05%	0%	0%	0%	0%	0.08%	0%	0%	0.06%	0.30%
6	0.2.2.2.	*Agromyces*	0	0.04%	0.04%	0.13%	0.14%	0%	0%	0%	0%	0%	0.07%	0%	0.06%	0%
6	0.2.2.2.	*Microbacterium*	0	0.16%	0.38%	0.76%	0.23%	0.15%	0%	0%	0%	0%	0.20%	0.02%	0%	0.15%
6	0.2.2.2.	*unclassified*	0	0.40%	0.59%	0.99%	0.64%	1.05%	0%	0.05%	0%	0.17%	0.27%	0.02%	0.13%	0.89%
6	0.2.2.2.	*Arthrobacter*	0	0.73%	0.84%	0.91%	3.16%	0.30%	0%	0.18%	0.06%	0.17%	0.27%	0.42%	0.06%	2.38%
6	0.2.2.2.	*Nesterenkonia*	0	0.18%	0.03%	0.13%	0%	0%	0%	0.32%	0.38%	0.41%	0.27%	0.12%	0.19%	0.30%
6	0.2.2.2.	*Cellulosimicrobium*	0	0.09%	0.31%	0.17%	0.46%	0%	0%	0%	0%	0%	0%	0%	0%	0.15%
6	0.2.2.2.	*unclassified*	0	0.01%	0.03%	0.12%	0%	0%	0%	0%	0%	0%	0%	0%	0%	0%
6	0.2.2.2.	*Actinoplanes*	0	0.04%	0%	0.03%	0.23%	0%	0%	0%	0%	0%	0%	0%	0.06%	0.15%
6	0.2.2.2.	*Dactylosporangium*	0	0.05%	0.03%	0.10%	0.37%	0%	0%	0%	0%	0%	0.07%	0%	0%	0%
6	0.2.2.2.	*Virgisporangium*	0	0.04%	0.11%	0.12%	0.09%	0%	0%	0%	0%	0%	0%	0%	0%	0.15%
6	0.2.2.2.	*unclassified*	0	0.30%	0.60%	1.07%	1.51%	0%	0%	0%	0%	0%	0.07%	0.02%	0%	0.30%
6	0.2.2.2.	*Aeromicrobium*	0	0.07%	0.15%	0.23%	0.32%	0%	0%	0%	0.06%	0%	0.07%	0.02%	0%	0%
6	0.2.2.2.	*Kribbella*	0	0.02%	0.06%	0.12%	0%	0%	0%	0%	0%	0%	0%	0%	0.06%	0%
6	0.2.2.2.	*Nocardioides*	0	0.14%	0.22%	0.50%	0.28%	0%	0%	0.05%	0%	0.08%	0.07%	0.05%	0%	0.45%
6	0.2.2.2.	*Actinophytocola*	0	0.01%	0.04%	0.08%	0%	0%	0%	0%	0%	0%	0%	0%	0%	0%
6	0.2.2.2.	*Lentzea*	0	0.13%	1.36%	0.18%	0.05%	0%	0%	0%	0%	0%	0%	0%	0%	0%
6	0.2.2.2.	*Streptomyces*	0	0.18%	0.59%	0.41%	0.50%	0%	0%	0.14%	0.13%	0.08%	0%	0.25%	0%	0%
6	0.2.2.2.	*Nonomuraea*	0	0.01%	0%	0.05%	0.09%	0%	0%	0%	0%	0%	0%	0%	0%	0%
6	0.2.2.2.	*Actinocorallia*	0	0.04%	0.04%	0.40%	0%	0%	0%	0%	0%	0%	0%	0%	0%	0%
6	0.2.2.2.	*unclassified*	0	0.03%	0.08%	0.07%	0.05%	0%	0%	0%	0%	0%	0%	0%	0%	0.15%
6	0.2.2.3.	*Conexibacter*	0	0.01%	0.06%	0.07%	0.05%	0%	0%	0%	0%	0%	0%	0%	0%	0%
6	0.2.2.3.	*unclassified*	0	0.09%	0.29%	0.35%	0.41%	0%	0%	0%	0%	0%	0%	0%	0%	0%
6	0.2.2.4.	*unclassified*	0	0.02%	0.07%	0.08%	0.05%	0%	0%	0%	0%	0%	0%	0%	0%	0%
6	0.2.3.1.	*Armatimonas*	0	0.01%	0%	0.03%	0.09%	0%	0%	0%	0%	0%	0%	0%	0%	0%
6	0.2.3.2.	*unclassified*	0	0.08%	0.18%	0.43%	0.14%	0%	0%	0%	0%	0%	0.07%	0.05%	0.06%	0%
6	0.2.4.1.	*Bacteroides*	0	0.05%	0.04%	0%	0%	0.15%	0%	0%	0%	0.17%	0%	0%	0.19%	0%
6	0.2.4.1.	*Prevotella*	0	0.01%	0%	0%	0.09%	0%	0%	0%	0%	0%	0%	0%	0%	0%
6	0.2.4.2.	*Chryseolinea*	0	0.04%	0.10%	0.03%	0.28%	0%	0%	0%	0%	0%	0%	0.10%	0%	0%
6	0.2.4.2.	*Dyadobacter*	0	0.23%	0.67%	0.84%	0.73%	0.30%	0%	0.09%	0%	0%	0.13%	0%	0%	0%
6	0.2.4.2.	*Emticicia*	0	0.02%	0.15%	0.02%	0.05%	0%	0%	0%	0%	0%	0%	0%	0%	0%
6	0.2.4.2.	*Hymenobacter*	0	0.01%	0%	0%	0%	0%	0%	0.05%	0%	0%	0%	0.05%	0%	0%
6	0.2.4.2.	*Larkinella*	0	0%	0%	0.03%	0%	0%	0%	0%	0%	0%	0%	0%	0%	0%
6	0.2.4.2.	*Ohtaekwangia*	0	0.14%	0.53%	0.58%	0.46%	0%	0%	0.05%	0%	0%	0%	0.02%	0%	0%
6	0.2.4.2.	*unclassified*	0	0.05%	0.17%	0.33%	0%	0%	0%	0.05%	0%	0%	0.07%	0%	0%	0%
6	0.2.4.2.	*unclassified*	0	0.02%	0.06%	0.15%	0.05%	0%	0%	0%	0%	0%	0%	0%	0%	0%
6	0.2.4.3.	*Fluviicola*	0	0.03%	0.15%	0.15%	0.05%	0%	0%	0%	0%	0%	0%	0%	0%	0%
6	0.2.4.3.	*Chryseobacterium*	0	0.30%	1.77%	0.53%	0.64%	0.45%	0%	0%	0%	0%	0.07%	0.05%	0.06%	0%
6	0.2.4.3.	*Epilithonimonas*	0	0.13%	0.63%	0.76%	0.09%	0%	0%	0%	0%	0%	0.07%	0%	0.06%	0%
6	0.2.4.3.	*Flavobacterium*	0	2.68%	9.98%	8.24%	7.75%	3.92%	0%	0.18%	0.19%	0.08%	0.67%	0.57%	0.25%	0.30%

6	0.2.4.3.	*unclassified*	0	0.23%	0.76%	0.66%	0.87%	0.30%	0%	0.09%	0.06%	0%	0.07%	0%	0%	0%
6	0.2.4.4.	*Chitinophaga*	0	0.18%	0.91%	0.51%	0.55%	0%	0%	0.05%	0%	0%	0%	0%	0.13%	0%
6	0.2.4.4.	*Ferruginibacter*	0	0.01%	0.07%	0.03%	0%	0%	0%	0%	0%	0%	0%	0%	0%	0%
6	0.2.4.4.	*Filimonas*	0	0.01%	0.01%	0.07%	0%	0%	0%	0%	0%	0%	0%	0.05%	0%	0%
6	0.2.4.4.	*Taibaiella*	0	0.17%	0.81%	0.61%	0.55%	0%	0%	0%	0.06%	0%	0%	0%	0%	0%
6	0.2.4.4.	*Terrimonas*	0	0.05%	0.07%	0.20%	0.23%	0%	0%	0%	0%	0%	0.07%	0%	0%	0%
6	0.2.4.4.	*unclassified*	0	0.32%	1.49%	1.12%	0.92%	0%	0%	0.09%	0%	0%	0%	0.02%	0.25%	0%
6	0.2.4.4.	*unclassified*	0	0.01%	0.03%	0%	0.14%	0%	0%	0%	0%	0%	0%	0%	0%	0%
6	0.2.4.4.	*unclassified*	0	0.02%	0.04%	0%	0.18%	0%	0%	0%	0%	0%	0%	0%	0%	0%
6	0.2.4.4.	*Arcticibacter*	0	0.01%	0%	0%	0%	0%	0%	0%	0%	0%	0%	0%	0.13%	0%
6	0.2.4.4.	*Mucilaginibacter*	0	0.02%	0.03%	0.12%	0.09%	0%	0%	0%	0%	0%	0%	0%	0%	0%
6	0.2.4.4.	*Pedobacter*	0	0.90%	2.66%	3.99%	1.83%	1.05%	0%	0.05%	0%	0.17%	0.20%	0.30%	0.13%	0.45%
6	0.2.4.4.	*Sphingobacterium*	0	0.37%	0.70%	1.57%	1.60%	0%	0%	0%	0%	0%	0.27%	0.05%	0.06%	0.15%
6	0.2.4.4.	*unclassified*	0	0.01%	0.04%	0.07%	0.05%	0%	0%	0%	0%	0%	0%	0%	0%	0%
6	0.2.4.4.	*unclassified*	0	0.02%	0.10%	0.07%	0.05%	0%	0%	0%	0%	0%	0%	0%	0%	0%
6	0.2.4.4.	*unclassified*	0	0.01%	0.08%	0.05%	0.05%	0%	0%	0%	0%	0%	0%	0%	0%	0%
6	0.2.4.5.	*unclassified*	0	0.04%	0.25%	0.18%	0.05%	0%	0%	0%	0%	0%	0%	0%	0%	0%
6	0.2.5.1.	*unclassified*	0	0.01%	0%	0.05%	0.05%	0%	0%	0%	0%	0%	0%	0%	0%	0%
6	0.2.6.1.	*unclassified*	0	0.03%	0.22%	0.12%	0%	0%	0%	0%	0%	0%	0%	0.07%	0%	0%
6	0.2.7.1.	*Candidatus_Saccharimona*	0	0.02%	0.01%	0%	0%	0%	0%	0%	0.06%	0.08%	0.07%	0.02%	0%	0%
6	0.2.7.2.	*unclassified*	0	0.10%	0.29%	0.18%	0.41%	0%	0%	0%	0.13%	0.08%	0%	0.07%	0%	0%
6	0.2.8.1.	*Candidatus_Protochlamyd*	0	0.02%	0.14%	0.05%	0%	0%	0%	0%	0%	0%	0%	0%	0%	0%
6	0.2.8.1.	*unclassified*	0	0.01%	0%	0.03%	0.05%	0%	0%	0%	0%	0%	0%	0%	0%	0%
6	0.2.9.1.	*unclassified*	0	0%	0.04%	0.02%	0%	0%	0%	0%	0%	0%	0%	0%	0%	0%
6	0.2.9.2.	*unclassified*	0	0.03%	0.01%	0.03%	0.28%	0%	0%	0%	0%	0%	0%	0%	0%	0%
6	0.2.10.1.	*unclassified*	0	0.01%	0%	0.02%	0.05%	0%	0%	0%	0%	0%	0%	0.02%	0%	0%
6	0.2.10.2.	*Caldilinea*	0	0%	0.01%	0%	0.05%	0%	0%	0%	0%	0%	0%	0%	0%	0%
6	0.2.10.2.	*unclassified*	0	0.01%	0.04%	0.05%	0%	0%	0%	0%	0%	0%	0%	0%	0%	0%
6	0.2.10.3.	*Candidatus_Chlorothrix*	0	0.02%	0.11%	0.05%	0.09%	0%	0%	0%	0%	0%	0%	0%	0%	0%
6	0.2.10.3.	*Roseiflexus*	0	0.05%	0.10%	0.10%	0.14%	0%	0%	0%	0%	0.08%	0%	0%	0%	0.15%
6	0.2.10.4.	*unclassified*	0	0.01%	0.03%	0.03%	0%	0%	0%	0%	0%	0%	0%	0%	0%	0%
6	0.2.10.5.	*Thermosporothrix*	0	0.01%	0%	0%	0%	0%	0%	0%	0%	0%	0%	0.12%	0%	0%
6	0.2.10.6.	*unclassified*	0	0.03%	0.06%	0.15%	0.09%	0%	0%	0%	0%	0%	0.07%	0%	0%	0%
6	0.2.10.7.	*unclassified*	0	0.02%	0.11%	0.10%	0%	0%	0%	0%	0%	0%	0%	0%	0%	0%
6	0.2.10.7.	*unclassified*	0	0.36%	0.63%	1.27%	1.51%	0%	0%	0%	0%	0%	0.07%	0.07%	0%	0.74%
6	0.2.10.8.	*unclassified*	0	0.03%	0.15%	0.20%	0.05%	0%	0%	0%	0%	0%	0%	0%	0%	0%
6	0.2.11.1.	*Bacillus*	0	1.34%	0.13%	0%	0%	0.15%	0%	4.30%	0.06%	0.08%	0.27%	10.82%	0.25%	0%
6	0.2.11.1.	*Paenibacillus*	0	0.04%	0.08%	0.20%	0.14%	0%	0%	0%	0%	0%	0%	0%	0%	0%
6	0.2.11.1.	*Saccharibacillus*	0	0.23%	1.32%	0.46%	0.87%	0%	0%	0%	0%	0%	0.07%	0%	0%	0%
6	0.2.11.1.	*unclassified*	0	0.08%	0.06%	0.02%	0%	0%	0%	0.09%	0%	0%	0.07%	0.35%	0.19%	0.15%
6	0.2.11.1.	*Staphylococcus*	0	0.05%	0.06%	0.02%	0%	0%	0%	0.14%	0%	0.17%	0.07%	0.07%	0.06%	0%
6	0.2.11.1.	*Carnobacterium*	0	0.06%	0%	0.02%	0%	0%	0%	0.05%	0%	0%	0.07%	0.17%	0.06%	0.30%
6	0.2.11.1.	*unclassified*	0	0.39%	0%	0%	0%	0%	0%	1.42%	0%	0%	0%	3.32%	0%	0%
6	0.2.11.1.	*Streptococcus*	0	0.02%	0.01%	0%	0%	0%	0%	0%	0.06%	0%	0.07%	0%	0.06%	0%
6	0.2.11.2.	*Incertae_Sedis*	0	0.01%	0.06%	0.05%	0.05%	0%	0%	0%	0%	0%	0%	0%	0%	0%
6	0.2.11.2.	*Ruminococcus*	0	0%	0.03%	0%	0%	0%	0%	0%	0%	0%	0%	0%	0%	0%
6	0.2.11.2.	*unclassified*	0	0.01%	0%	0%	0%	0%	0%	0.09%	0%	0%	0%	0.05%	0%	0%
6	0.2.11.3.	*unclassified*	0	0.02%	0%	0%	0%	0%	0%	0.05%	0.13%	0%	0%	0.02%	0%	0%
6	0.2.12.1.	*Fusobacterium*	0	0.05%	0.06%	0%	0%	0%	0%	0.09%	0.13%	0.17%	0%	0%	0%	0.15%
6	0.2.13.1.	*Gemmatimonas*	0	0%	0.01%	0.03%	0%	0%	0%	0%	0%	0%	0%	0%	0%	0%
6	0.2.14.1.	*Nitrospira*	0	0%	0%	0.05%	0%	0%	0%	0%	0%	0%	0%	0%	0%	0%
6	0.2.15.1.	*unclassified*	0	0.05%	0.03%	0.31%	0.23%	0%	0%	0%	0%	0%	0%	0%	0%	0%
6	0.2.15.2.	*Gemmata*	0	0.03%	0.06%	0.26%	0.05%	0%	0%	0%	0%	0%	0%	0%	0%	0%
6	0.2.15.2.	*Pirellula*	0	0.01%	0.06%	0.03%	0.05%	0%	0%	0%	0%	0%	0%	0%	0%	0%
6	0.2.15.2.	*Planctomyces*	0	0.07%	0.24%	0.35%	0.18%	0%	0%	0.05%	0.06%	0%	0%	0%	0%	0%
6	0.2.15.2.	*Singulisphaera*	0	0.09%	0.17%	0.35%	0.37%	0%	0%	0%	0%	0.08%	0%	0.02%	0%	0.15%
6	0.2.15.2.	*unclassified*	0	0.18%	0.34%	0.86%	0.69%	0%	0%	0%	0%	0%	0.13%	0.05%	0%	0.15%
6	0.2.15.3.	*unclassified*	0	0.01%	0.11%	0.05%	0%	0%	0%	0%	0%	0%	0%	0%	0%	0%
6	0.2.16.1.	*Asticcacaulis*	0	0.05%	0.22%	0.05%	0.28%	0%	0%	0%	0%	0%	0%	0%	0%	0%
6	0.2.16.1.	*Brevundimonas*	0	0.08%	0.17%	0.13%	0.14%	0.30%	0%	0.05%	0.06%	0.08%	0%	0.05%	0%	0%
6	0.2.16.1.	*Caulobacter*	0	0.06%	0.22%	0.08%	0.37%	0%	0%	0%	0%	0%	0%	0%	0%	0%
6	0.2.16.1.	*Phenylobacterium*	0	0.01%	0.06%	0.02%	0.05%	0%	0%	0%	0%	0%	0%	0%	0%	0%
6	0.2.16.1.	*Woodsholea*	0	0.06%	0.10%	0.18%	0.46%	0%	0%	0%	0%	0%	0%	0%	0%	0%
6	0.2.16.1.	*unclassified*	0	0.03%	0.03%	0.05%	0.23%	0%	0%	0%	0%	0%	0%	0.05%	0%	0%
6	0.2.16.1.	*Chelatococcus*	0	0.02%	0.03%	0.02%	0.18%	0%	0%	0%	0%	0%	0%	0%	0%	0%
6	0.2.16.1.	*Bosea*	0	0.12%	0.32%	0.26%	0.64%	0%	0%	0%	0%	0%	0.13%	0.02%	0%	0%
6	0.2.16.1.	*Bradyrhizobium*	0	0.09%	0.27%	0.30%	0.32%	0%	0%	0.09%	0%	0%	0%	0.05%	0%	0%
6	0.2.16.1.	*Rhodopseudomonas*	0	0.06%	0.13%	0.28%	0.28%	0%	0%	0%	0.06%	0%	0%	0%	0%	0%
6	0.2.16.1.	*unclassified*	0	0%	0%	0.03%	0%	0%	0%	0%	0%	0%	0%	0%	0%	0%
6	0.2.16.1.	*Ochrobactrum*	0	0.24%	0.34%	0.61%	0.60%	0.75%	0%	0%	0.06%	0.17%	0.13%	0.07%	0%	0.15%
6	0.2.16.1.	*Angulomicrobium*	0	0.06%	0.07%	0.21%	0.05%	0.30%	0%	0%	0%	0%	0%	0%	0.06%	0%
6	0.2.16.1.	*Devosia*	0	0.38%	0.95%	1.31%	1.42%	0.75%	0%	0.09%	0.06%	0%	0%	0.02%	0%	0%
6	0.2.16.1.	*Hyphomicrobium*	0	0.21%	0.57%	0.58%	0.55%	0%	0%	0.09%	0.13%	0%	0.07%	0.05%	0.06%	0.45%
6	0.2.16.1.	*Pelagibacterium*	0	0.78%	0.36%	0.41%	0.14%	2.26%	0.55%	0.78%	0.19%	1.66%	1.00%	0.55%	1.32%	0.15%
6	0.2.16.1.	*unclassified*	0	0.07%	0.14%	0.23%	0.37%	0%	0%	0.09%	0%	0%	0%	0%	0.06%	0%
6	0.2.16.1.	*Methylobacterium*	0	0.04%	0.08%	0.07%	0.09%	0%	0%	0%	0%	0%	0.07%	0.05%	0%	0.15%
6	0.2.16.1.	*Microvirga*	0	0.03%	0.03%	0%	0.23%	0%	0%	0%	0%	0%	0.07%	0%	0%	0%
6	0.2.16.1.	*Mesorhizobium*	0	0.12%	0.10%	0.36%	0.64%	0%	0%	0.09%	0%	0.17%	0%	0%	0.06%	0%
6	0.2.16.1.	*unclassified*	0	0.77%	0.36%	0.61%	0.46%	1.66%	0.55%	0.82%	0.63%	0.91%	0.86%	0.65%	0.50%	1.19%
6	0.2.16.1.	*Kaistia*	0	0.07%	0.15%	0.18%	0.37%	0.15%	0%	0%	0%	0%	0%	0%	0%	0%
6	0.2.16.1.	*Rhizobium*	0	1.16%	2.87%	2.53%	2.66%	3.01%	0%	0.05%	0.13%	0.17%	0.27%	0.32%	0.25%	1.64%
6	0.2.16.1.	*Shinella*	0	0.11%	0.15%	0.50%	0.41%	0.15%	0%	0%	0%	0%	0%	0.05%	0%	0%
6	0.2.16.1.	*Nordella*	0	0.01%	0.03%	0.12%	0%	0%	0%	0%	0%	0%	0%	0%	0%	0%
6	0.2.16.1.	*Rhizomicrobium*	0	0.05%	0.17%	0.28%	0.05%	0%	0%	0%	0.06%	0%	0%	0%	0.06%	0%
6	0.2.16.1.	*unclassified*	0	0.01%	0%	0.05%	0.09%	0%	0%	0%	0%	0%	0%	0%	0%	0%
6	0.2.16.1.	*Azorhizobium*	0	0.01%	0.03%	0%	0%	0%	0%	0%	0%	0%	0%	0%	0%	0.15%
6	0.2.16.1.	*Labrys*	0	0.02%	0.18%	0.05%	0.05%	0%	0%	0%	0%	0%	0%	0%	0%	0%
6	0.2.16.1.	*unclassified*	0	0.46%	1.50%	2.05%	1.24%	0%	0%	0%	0.06%	0.17%	0%	0.12%	0.06%	0.30%
6	0.2.16.1.	*Amaricoccus*	0	0.04%	0.01%	0.02%	0.41%	0%	0%	0%	0%	0%	0%	0%	0%	0%

6	0.2.16.1.	*unclassified*	0	0.05%	0%	0.03%	0.46%	0%	0%	0.05%	0%	0%	0.07%	0%	0%	0%
6	0.2.16.1.	*unclassified*	0	0.03%	0.06%	0.25%	0.05%	0%	0%	0.05%	0%	0%	0%	0%	0%	0%
6	0.2.16.1.	*Roseomonas*	0	0.01%	0.03%	0.10%	0.05%	0%	0%	0%	0%	0%	0%	0%	0%	0%
6	0.2.16.1.	*unclassified*	0	0%	0.01%	0.02%	0%	0%	0%	0%	0%	0%	0%	0%	0%	0%
6	0.2.16.1.	*Azospirillum*	0	0%	0.01%	0%	0.05%	0%	0%	0%	0%	0%	0%	0%	0%	0%
6	0.2.16.1.	*Dongia*	0	0.05%	0.22%	0.25%	0.09%	0%	0%	0%	0%	0%	0%	0%	0%	0%
6	0.2.16.1.	*Skermanella*	0	0.02%	0.07%	0.13%	0.09%	0%	0%	0%	0%	0%	0%	0%	0%	0%
6	0.2.16.1.	*Candidatus_Alysiosphaera*	0	0%	0.01%	0%	0.05%	0%	0%	0%	0%	0%	0%	0%	0%	0%
6	0.2.16.1.	*Reyranella*	0	0.05%	0.13%	0.23%	0.23%	0%	0%	0%	0%	0%	0%	0%	0%	0%
6	0.2.16.1.	*unclassified*	0	0.04%	0.11%	0.26%	0.09%	0%	0%	0%	0%	0%	0.07%	0%	0%	0%
6	0.2.16.1.	*Porphyrobacter*	0	0.04%	0.08%	0.08%	0.28%	0%	0%	0%	0%	0%	0%	0%	0%	0%
6	0.2.16.1.	*Novosphingobium*	0	0.10%	0.22%	0.21%	0.55%	0%	0%	0%	0%	0.08%	0.07%	0%	0.06%	0%
6	0.2.16.1.	*Sphingobium*	0	0.16%	0.10%	0.08%	0.14%	0%	0%	0%	0.06%	0%	0%	0%	0%	1.49%
6	0.2.16.1.	*Sphingomonas*	0	0.18%	0.42%	0.68%	0.96%	0%	0%	0%	0%	0%	0.07%	0.02%	0.06%	0%
6	0.2.16.1.	*Sphingopyxis*	0	0.06%	0.18%	0.17%	0.23%	0%	0%	0%	0%	0%	0%	0%	0.13%	0%
6	0.2.16.1.	*unclassified*	0	0.06%	0.20%	0.07%	0.23%	0%	0%	0%	0.06%	0%	0%	0%	0.13%	0%
6	0.2.16.1.	*unclassified*	0	0.34%	0.10%	0.31%	0.69%	0%	0%	0%	0.19%	0.50%	2.33%	0%	0%	0%
6	0.2.16.2.	*Achromobacter*	0	0.35%	0.97%	1.44%	1.10%	0.30%	0%	0.05%	0%	0.08%	0.13%	0.02%	0.06%	0%
6	0.2.16.2.	*Burkholderia*	0	0.05%	0.03%	0.05%	0.09%	0%	0%	0%	0%	0%	0.40%	0%	0.06%	0%
6	0.2.16.2.	*Ralstonia*	0	0.02%	0%	0%	0%	0%	0%	0.05%	0.06%	0%	0.07%	0.05%	0%	0%
6	0.2.16.2.	*Acidovorax*	0	0.09%	0.49%	0.28%	0%	0%	0%	0%	0.06%	0.08%	0.07%	0%	0.06%	0%
6	0.2.16.2.	*Aquabacterium*	0	0.02%	0.07%	0.05%	0.05%	0%	0%	0.05%	0%	0%	0%	0%	0%	0%
6	0.2.16.2.	*Comamonas*	0	0.16%	0.48%	0.18%	0.32%	0.90%	0%	0%	0%	0%	0%	0%	0%	0%
6	0.2.16.2.	*Delftia*	0	0.47%	0.70%	2.61%	1.28%	0%	0%	0.09%	0%	0.17%	0.73%	0.05%	0.06%	0%
6	0.2.16.2.	*Hydrogenophaga*	0	0.06%	0.18%	0.17%	0.32%	0%	0%	0%	0%	0%	0.07%	0%	0%	0%
6	0.2.16.2.	*Mitsuaria*	0	0.02%	0.13%	0.03%	0.05%	0%	0%	0%	0%	0%	0%	0%	0%	0%
6	0.2.16.2.	*Pseudorhodoferax*	0	0.05%	0.22%	0.25%	0.18%	0%	0%	0%	0%	0%	0%	0%	0%	0%
6	0.2.16.2.	*Rhizobacter*	0	0.08%	0.35%	0.26%	0.23%	0%	0%	0%	0.06%	0%	0.07%	0%	0%	0%
6	0.2.16.2.	*Variovorax*	0	0.06%	0.45%	0.15%	0.14%	0%	0%	0%	0%	0%	0%	0%	0%	0%
6	0.2.16.2.	*unclassified*	0	0.13%	0.46%	0.38%	0.46%	0.15%	0%	0%	0%	0%	0%	0.05%	0%	0%
6	0.2.16.2.	*Duganella*	0	0.06%	0.32%	0.12%	0.18%	0%	0%	0%	0%	0%	0%	0.05%	0%	0%
6	0.2.16.2.	*Herminiimonas*	0	0.29%	0.01%	0.02%	0%	3.46%	0%	0%	0%	0%	0%	0.02%	0%	0%
6	0.2.16.2.	*Janthinobacterium*	0	0.23%	0.35%	0.83%	1.15%	0%	0%	0%	0%	0.17%	0.20%	0.07%	0%	0%
6	0.2.16.2.	*Massilia*	0	0.16%	0.43%	0.68%	0.28%	0%	0%	0%	0%	0%	0.40%	0%	0%	0.15%
6	0.2.16.2.	*Methylobacillus*	0	0.01%	0.04%	0.05%	0%	0%	0%	0%	0%	0%	0.07%	0%	0%	0%
6	0.2.16.2.	*Methylophilus*	0	0.38%	0.48%	0.40%	0.60%	2.86%	0%	0%	0.06%	0%	0.07%	0%	0.13%	0%
6	0.2.16.2.	*Methylotenera*	0	0.04%	0%	0.05%	0.18%	0.30%	0%	0%	0%	0%	0%	0%	0%	0%
6	0.2.16.2.	*unclassified*	0	0.05%	0.29%	0.13%	0.05%	0%	0%	0%	0%	0%	0%	0%	0%	0.15%
6	0.2.16.2.	*Nitrosospira*	0	0.02%	0.01%	0.08%	0.09%	0%	0%	0%	0%	0%	0%	0%	0%	0%
6	0.2.16.2.	*unclassified*	0	0.12%	0.45%	0.28%	0.32%	0.30%	0%	0%	0%	0%	0%	0%	0.06%	0%
6	0.2.16.2.	*unclassified*	0	0.02%	0.03%	0.12%	0%	0%	0%	0.05%	0%	0%	0%	0%	0%	0%
6	0.2.16.2.	*unclassified*	0	0.02%	0.10%	0.10%	0.05%	0%	0%	0%	0%	0%	0%	0%	0%	0%
6	0.2.16.3.	*Bdellovibrio*	0	0.01%	0.06%	0.03%	0%	0%	0%	0%	0%	0%	0%	0%	0%	0%
6	0.2.16.3.	*OM27_clade*	0	0.01%	0.04%	0.05%	0.05%	0%	0%	0%	0%	0%	0%	0.02%	0%	0%
6	0.2.16.3.	*Nannocystis*	0	0.04%	0.08%	0.30%	0.05%	0%	0%	0%	0%	0%	0%	0%	0%	0%
6	0.2.16.3.	*Byssovorax*	0	0.05%	0.25%	0.23%	0.09%	0%	0%	0%	0%	0%	0%	0%	0%	0%
6	0.2.16.3.	*Chondromyces*	0	0.01%	0%	0.03%	0.09%	0%	0%	0%	0%	0%	0%	0%	0%	0%
6	0.2.16.3.	*unclassified*	0	0.04%	0.13%	0.13%	0.18%	0%	0%	0%	0%	0%	0%	0%	0%	0%
6	0.2.16.3.	*unclassified*	0	0.12%	0.25%	0.68%	0.46%	0%	0%	0.05%	0%	0%	0%	0.05%	0%	0%
6	0.2.16.3.	*unclassified*	0	0.01%	0.04%	0.13%	0%	0%	0%	0%	0%	0%	0%	0%	0%	0%
6	0.2.16.4.	*Enterobacter*	0	0.43%	0.99%	2.61%	1.10%	0%	0%	0%	0%	0.08%	0.20%	0.07%	0.06%	0%
6	0.2.16.4.	*Pantoea*	0	0.71%	2.83%	0.43%	1.24%	1.20%	0%	0.23%	0.13%	0.33%	0.47%	0.20%	0.13%	1.34%
6	0.2.16.4.	*Pectobacterium*	0	0.01%	0.01%	0.10%	0%	0%	0%	0%	0%	0%	0%	0%	0%	0%
6	0.2.16.4.	*Serratia*	0	0.25%	0.29%	0.40%	1.15%	0.15%	0%	0.09%	0%	0.17%	0.40%	0.12%	0.06%	0.15%
6	0.2.16.4.	*unclassified*	0	0.98%	8.87%	2.60%	0.09%	0%	0%	0.14%	0%	0%	0.07%	0.05%	0%	0%
6	0.2.16.4.	*unclassified*	0	0%	0%	0.03%	0%	0%	0%	0%	0%	0%	0%	0%	0%	0%
6	0.2.16.4.	*Halomonas*	0	1.52%	0.76%	0.66%	0.09%	1.96%	1.10%	1.60%	1.19%	2.16%	3.73%	1.67%	2.01%	1.34%
6	0.2.16.4.	*Pseudospirillum*	0	0.01%	0.01%	0.08%	0%	0%	0%	0%	0%	0%	0%	0%	0%	0%
6	0.2.16.4.	*Acinetobacter*	0	0.24%	0.31%	0.15%	0.05%	0%	0%	0.37%	0.31%	0.17%	0.07%	0.45%	0.13%	0.89%
6	0.2.16.4.	*Psychrobacter*	0	0.09%	0.01%	0.05%	0%	0%	0%	0.14%	0%	0%	0%	0.22%	0.25%	0.45%
6	0.2.16.4.	*Cellvibrio*	0	0.05%	0.13%	0.08%	0.32%	0%	0%	0.05%	0%	0%	0%	0.07%	0%	0%
6	0.2.16.4.	*Pseudomonas*	0	2.67%	7.78%	5.28%	9.12%	1.36%	0%	0.46%	0.06%	0.58%	1.53%	0.90%	2.32%	2.68%
6	0.2.16.4.	*unclassified*	0	0.01%	0.04%	0.03%	0%	0%	0%	0.05%	0%	0%	0%	0.02%	0%	0%
6	0.2.16.4.	*Arenimonas*	0	0.02%	0.08%	0.02%	0.05%	0%	0%	0%	0%	0%	0.07%	0%	0%	0%
6	0.2.16.4.	*Dokdonella*	0	0.06%	0.08%	0.15%	0.28%	0%	0%	0.05%	0%	0.08%	0%	0.02%	0%	0%
6	0.2.16.4.	*Luteimonas*	0	0.02%	0.06%	0.10%	0.05%	0%	0%	0%	0%	0%	0%	0%	0%	0%
6	0.2.16.4.	*Lysobacter*	0	0.01%	0.03%	0.08%	0.05%	0%	0%	0%	0%	0%	0%	0%	0%	0%
6	0.2.16.4.	*Pseudofulvimonas*	0	0.01%	0.06%	0%	0.05%	0%	0%	0%	0%	0%	0%	0%	0%	0%
6	0.2.16.4.	*Pseudoxanthomonas*	0	0.12%	0.31%	0.50%	0.69%	0%	0%	0%	0%	0%	0%	0%	0%	0%
6	0.2.16.4.	*Stenotrophomonas*	0	0.55%	1.14%	1.72%	2.25%	0.60%	0%	0.09%	0.06%	0.08%	0.40%	0.10%	0.13%	0%
6	0.2.16.4.	*Xanthomonas*	0	0.01%	0%	0%	0%	0%	0%	0%	0%	0%	0.07%	0.05%	0%	0%
6	0.2.16.4.	*unclassified*	0	0.05%	0.24%	0.28%	0.09%	0%	0%	0%	0%	0%	0%	0%	0%	0%
6	0.2.16.4.	*Steroidobacter*	0	0.03%	0.27%	0.13%	0%	0%	0%	0%	0%	0%	0%	0%	0%	0%
6	0.2.16.4.	*unclassified*	0	0.15%	0.31%	0.71%	0.64%	0%	0%	0.05%	0%	0%	0.07%	0%	0%	0%
6	0.2.16.5.	*unclassified*	0	0.06%	0.11%	0.41%	0.18%	0%	0%	0%	0%	0%	0.07%	0%	0%	0%
6	0.2.17.1.	*unclassified*	0	0.01%	0%	0%	0%	0%	0%	0%	0%	0%	0.13%	0%	0%	0%
6	0.2.17.2.	*unclassified*	0	0.01%	0%	0.02%	0.05%	0%	0%	0%	0%	0%	0%	0%	0%	0%
6	0.2.17.3.	*unclassified*	0	0.04%	0.17%	0.18%	0.14%	0%	0%	0%	0%	0%	0%	0%	0%	0%
6	0.2.17.3.	*Candidatus_Xiphinematoba*	0	0.02%	0.04%	0.17%	0%	0%	0%	0.05%	0%	0%	0%	0%	0%	0%
6	0.2.17.3.	*unclassified*	0	0.01%	0%	0.07%	0%	0%	0%	0%	0%	0%	0%	0%	0%	0%
6	0.2.17.4.	*Haloferula*	0	0.02%	0.04%	0.12%	0%	0%	0%	0%	0%	0%	0%	0.05%	0%	0%
6	0.2.17.4.	*Luteolibacter*	0	0.01%	0.11%	0.07%	0%	0%	0%	0%	0%	0%	0%	0%	0%	0%
6	0.2.17.4.	*Prosthecobacter*	0	0.02%	0.10%	0.08%	0.09%	0%	0%	0%	0%	0%	0%	0%	0%	0%
6	0.2.17.4.	*Roseimicrobium*	0	0.10%	0.17%	0.88%	0.18%	0%	0%	0%	0%	0%	0%	0%	0%	0%
6	0.2.17.4.	*unclassified*	0	0.11%	0.32%	0.46%	0.28%	0%	0%	0%	0%	0%	0%	0.07%	0%	0.15%
6	0.2.17.5.	*unclassified*	0	0.02%	0.07%	0.18%	0%	0%	0%	0%	0%	0%	0%	0%	0%	0%
6	0.2.18.1.	*unclassified*	0	61.77%	17.18%	14.39%	20.77%	68.83%	97.25%	80.29%	91.33%	64.98%	55.36%	69.14%	86.40%	75.30%
6	0.3.1.1.	*unclassified*	0	6.16%	0.99%	4.12%	0.32%	0%	0.55%	5.67%	3.20%	24.56%	25.02%	6.53%	2.32%	0.60%

Supplementary Table S2 Bacterial communities with significantly difference in abundance at various level in the four four *Allium* species

	taxon	Avrage								Standard error				SAS analysis		
		CL		WO		GA		ON		CL	WO	GA	ON	LSD	F	P
Phyla	Proteobacteria	42.68029	a	10.31405	b	8.796497	b	9.195527	b	0.843861	6.376657	3.140546	1.880846	12.068	20.21	0.0004
	Bacteroidetes	20.18128	a	2.286857	b	0.775273	b	1.122443	b	1.48161	1.953979	0.411799	0.115158	4.0588	57.23	0.0001
	unclassified	17.44603	b	82.12356	a	70.55562	a	76.9479	a	1.847388	8.257203	10.75306	5.050663	23.783	16.84	0.0008
	Actinobacteria	10.27441	a	1.018407	b	1.398063	b	3.51075	b	1.025543	0.566555	0.423218	2.41194	4.4265	10	0.0044
	Chloroflexi	1.728897	a	0	b	0.07202	b	0.372397	b	0.240851	0	0.038804	0.268168	0.5911	19.8	0.0005
	Planctomycetes	1.58995	a	0.01524	b	0.092957	b	0.124133	b	0.352817	0.01524	0.020886	0.089389	0.595	17.25	0.0007
	Verrucomicrobia	1.324187	a	0.01524	b	0.044357	b	0.091147	b	0.453649	0.01524	0.044357	0.046105	0.7474	7.74	0.0094
	Firmicutes	1.184313	a	2.09257	a	0.36634	a	5.29286	a	0.28939	2.017738	0.116232	4.756644	8.4404	0.7	0.5806
Class	Flavobacteriia	11.0066	a	1.647673	b	0.39973	b	0.43223	b	1.170705	1.512573	0.237623	0.098091	3.1469	28.19	0.0001
	Sphingobacteriia	7.228433	a	0.412373	b	0.253687	b	0.569397	b	0.631459	0.325238	0.14244	0.077722	1.188	87.66	0.0001
	Cytophagia	1.741663	a	0.176607	b	0.066533	b	0.05816	b	0.126459	0.090755	0.066533	0.05816	0.2919	84.44	0.0001
	Deltaproteobacteria	1.119797	a	0.01524	b	0	b	0.024927	b	0.23457	0.01524	0	0.024927	0.3845	21.92	0.0003
	Gammaproteobacteria	19.36104	a	3.22072	b	4.154267	b	5.294513	b	2.642294	1.204934	1.548722	0.839195	5.5383	20.11	0.0004
	Planctomycetacia	1.34555	a	0.01524	b	0.092957	b	0.124133	b	0.288017	0.01524	0.020886	0.089389	0.4935	17.64	0.0007
	unclassified	17.44603	b	82.12356	a	70.55562	a	76.9479	a	1.847388	8.257203	10.75306	5.050663	23.783	16.84	0.0008
	Thermomicrobia	1.209453	a	0	b	0.022177	b	0.272943	b	0.236869	0	0.022177	0.23654	0.547	11.51	0.0028
	Alphaproteobacteria	14.3451	a	4.225587	b	3.616273	b	3.522677	b	1.544655	2.57704	1.000533	1.086438	5.4591	9.98	0.0044
	Actinobacteria	9.363293	a	1.018407	b	1.398063	b	3.44453	b	1.010651	0.566555	0.423218	2.37036	4.3571	8.33	0.0077
	Betaproteobacteria	7.618043	a	2.852493	b	1.003777	b	0.353417	b	0.540503	2.716473	0.633198	0.043269	4.6333	5.34	0.0259
	unclassified	1.811217	b	2.073107	b	17.59482	a	3.147983	b	1.16956	1.805363	7.196836	1.762843	12.581	3.93	0.054
	Bacilli	1.124467	a	2.046847	a	0.324463	a	5.267937	a	0.278162	1.972026	0.141713	4.731722	8.3742	0.71	0.571
Order	Flavobacteriales	11.0066	a	1.647673	b	0.39973	b	0.43223	b	1.170705	1.512573	0.237623	0.098091	3.1469	28.19	0.0001
	Sphingobacteriales	7.228433	a	0.412373	b	0.253687	b	0.569397	b	0.631459	0.325238	0.14244	0.077722	1.188	87.66	0.0001
	Xanthomonadales	2.90583	a	0.246527	b	0.253687	b	0.09993	b	0.356394	0.182294	0.14244	0.051942	0.698	40.07	0.0001
	Cytophagales	1.741663	a	0.176607	b	0.066533	b	0.05816	b	0.126459	0.090755	0.066533	0.05816	0.2919	84.44	0.0001
	Pseudomonadales	7.78285	a	0.802363	b	0.90686	b	2.79403	b	1.152089	0.410652	0.361133	0.679606	2.3563	20.5	0.0004
	Planctomycetales	1.34555	a	0.01524	b	0.092957	b	0.124133	b	0.288017	0.01524	0.020886	0.089389	0.4953	17.64	0.0007
	unclassified	17.44603	b	82.12356	a	70.55562	a	76.9479	a	1.847388	8.257203	10.75306	5.050663	23.783	16.84	0.0008
	Burkholderiales	6.320587	a	1.682633	b	0.938483	b	0.241157	b	0.587557	1.569711	0.601965	0.050803	2.9051	9.51	0.0051
	JG30-KF-CM45	1.139013	a	0	b	0.022177	b	0.272943	b	0.263452	0	0.022177	0.23654	0.5785	9.09	0.0059
	Micromonosporales	1.42217	a	0	b	0.044357	b	0.22761	b	0.423835	0	0.044357	0.184137	0.757	8.41	0.0074
	Rhizobiales	10.23667	a	4.07946	b	2.40441	b	2.85979	b	0.700138	2.495244	0.543775	0.667644	4.453	7.07	0.0122
	Sphingomonadales	1.62643	a	0	b	0.113897	b	0.629657	b	0.379689	0	0.015602	0.44102	0.9493	6.49	0.0155
	Enterobacteriales	7.57203	a	0.604223	b	0.612537	b	0.729127	b	2.814881	0.398117	0.290676	0.383768	4.7016	5.77	0.0212
	Corynebacteriales	2.32069	a	0.242053	b	0.390997	b	1.06375	ab	0.194799	0.131422	0.206789	0.808079	1.413	4.78	0.0341
	unclassified	1.811217	b	2.073107	b	17.59482	a	3.147983	b	1.16956	1.805363	7.196836	1.762843	12.581	3.93	0.054

	Micrococcales	3.46016	a	0.71539	b	0.800217	b	1.825867	ab	0.662724	0.436375	0.200132	1.244846	2.4292	2.94	0.099
	Bacillales	1.11428	a	1.559117	a	0.25917	a	3.96378	a	0.277861	1.484453	0.116428	3.640172	6.429	0.65	0.6056
	Flavobacteriaceae	10.89033	a	1.647673	b	0.39973	b	0.43223	b	1.145682	1.512573	0.237623	0.098091	3.1223	27.99	0.0001
	Comamonadaceae	3.641107	a	0.39713	b	0.435353	b	0.075003	b	0.390601	0.330923	0.2506	0.038223	0.9315	34.48	0.0001
	Xanthomonadaceae	2.77298	a	0.246527	b	0.253687	b	0.09993	b	0.432966	0.182294	0.14244	0.051942	0.8049	27.25	0.0001
	Chitinophagaceae	2.718877	a	0.045723	b	0.043113	b	0.15024	b	0.333616	0.045723	0.021583	0.114896	0.5812	54.9	0.0001
	Cytophagaceae	1.658083	a	0.176607	b	0.066533	b	0.05816	b	0.094522	0.090755	0.066533	0.05816	0.2577	97.59	0.0001
	unclassified	1.596	a	0	b	0.076263	b	0.161637	b	0.239506	0	0.048384	0.070306	0.4146	35.85	0.0001
	Alcaligenaceae	1.16869	a	0.11564	b	0.07202	b	0.029197	b	0.140411	0.093714	0.038804	0.018214	0.284	39.79	0.0001
	Nocardiaceae	1.42572	a	0.095923	b	0.166217	b	0.20293	b	0.188098	0.048118	0.096034	0.122219	0.4055	26.23	0.0002
	Pseudomonadaceae	7.59398	a	0.634707	b	0.724663	b	1.99796	b	1.183455	0.393632	0.429674	0.511167	2.3068	21.72	0.0003
	Sphingobacteriaceae	4.24934	a	0.366647	b	0.210573	b	0.419157	b	0.745664	0.34404	0.136284	0.088642	1.365	21.94	0.0003
	Paenibacillaceae	1.02385	a	0	b	0.022177	b	0	b	0.213713	0	0.022177	0	0.3504	22.39	0.0003
Familiy	Planctomycetaceae	1.34555	a	0.01524	b	0.092957	b	0.124133	b	0.288017	0.01524	0.020886	0.089389	0.4935	17.64	0.0007
	unclassified	17.44603	b	82.12356	a	70.55562	a	76.9479	a	1.847388	8.257203	10.75306	5.050663	23.783	16.84	0.0008
	unclassified	1.139013	a	0	b	0.022177	b	0.272943	b	0.263452	0	0.022177	0.23654	0.5785	9.09	0.0059
	Micromonosporaceae	1.42217	a	0	b	0.044357	b	0.22761	b	0.423835	0	0.044357	0.184137	0.757	8.41	0.0074
	Enterobacteriaceae	7.57203	a	0.604223	b	0.612537	b	0.729127	b	2.814881	0.398117	0.290676	0.383768	4.7016	5.77	0.0212
	Rhizobiaceae	3.276013	a	1.119657	b	0.185913	b	0.753803	b	0.081765	1.096876	0.041765	0.442979	1.9347	5.18	0.028
	Sphingomonadaceae	1.315273	a	0	b	0.092957	b	0.587887	ab	0.289113	0	0.020886	0.454798	0.8794	4.99	0.0308
	unclassified	1.811217	b	2.073107	b	17.59482	a	3.147983	b	1.16956	1.805363	7.196836	1.762843	12.581	3.93	0.054
	Microbacteriaceae	1.30097	a	0.416847	ab	0.23275	b	0.426497	ab	0.29213	0.394208	0.157238	0.31016	0.9806	2.54	0.1295
	Hyphomicrobiaceae	2.45632	a	1.638123	b	1.033723	b	0.907377	b	0.188521	0.850018	0.370653	0.2983	1.6179	2.03	0.1877
	Micrococcaceae	1.691453	a	0.268057	a	0.517627	a	1.159183	a	0.737811	0.146138	0.041416	0.764539	1.7501	1.43	0.3031
	Oxalobacteraceae	1.454343	a	1.15462	a	0.254927	a	0.099453	a	0.166894	1.15462	0.178488	0.049727	1.9261	1.27	0.3487
	Flavobacterium	8.653937	a	1.366187	b	0.312257	b	0.373843	b	0.676574	1.27583	0.179147	0.100637	2.3785	30.29	0.0001
	unclassified	1.596	a	0	b	0.076263	b	0.161637	b	0.239506	0	0.048384	0.070306	0.4146	35.83	0.0001
	Rhodococcus	1.340387	a	0.045723	b	0.11089	b	0.13671	b	0.141679	0.045723	0.11089	0.08054	0.33	37.85	0.0001
	unclassified	1.175653	a	0.030483	b	0	b	0.091853	b	0.166069	0.030483	0	0.079714	0.3045	37.11	0.0001
	Achromobacter	1.16869	a	0.11564	b	0.07202	b	0.029197	b	0.140411	0.093714	0.038804	0.018214	0.284	39.79	0.0001
	Pseudomonas	7.392353	a	0.604223	b	0.724663	b	1.964727	b	1.127647	0.398117	0.429674	0.543747	2.2537	21.53	0.0003
	Devosia	1.226903	a	0.281487	b	0.020937	b	0.00831	b	0.140958	0.237235	0.020937	0.00831	0.4515	17.29	0.0007
	unclassified	17.44603	b	82.12356	a	70.55562	a	76.9479	a	1.847388	8.257203	10.75306	5.050663	23.783	16.84	0.0008
	Stenotrophomonas	1.700543	a	0.231287	b	0.181667	b	0.075003	b	0.321034	0.18743	0.108923	0.038223	0.6347	15.12	0.001
Genus	Sphingobacterium	1.292157	a	0	b	0.08871	b	0.087107	b	0.295904	0	0.08871	0.031072	0.5063	15.86	0.001
	unclassified	1.06352	a	0	b	0.022177	b	0.107517	b	0.262898	0	0.022177	0.095324	0.4574	13.34	0.0018
	Pedobacter	2.827343	a	0.366647	b	0.12186	b	0.29028	b	0.626506	0.34404	0.061698	0.092804	1.1796	12.68	0.0021
	unclassified	1.139013	a	0	b	0.022177	b	0.272943	b	0.263452	0	0.022177	0.23654	0.5785	9.09	0.0059
	Enterobacter	1.569413	a	0	b	0.094197	b	0.045813	b	0.522616	0	0.057891	0.023172	0.8582	8.39	0.0075
	Delftia	1.53245	a	0.030483	b	0.299283	b	0.037503	b	0.565831	0.030483	0.221537	0.019113	0.9926	5.53	0.0236
	unclassified	1.811217	b	2.073107	b	17.59482	a	3.147983	b	1.16956	1.805363	7.196836	1.762843	12.581	3.93	0.054
	Rhizobium	2.687423	a	1.019257	ab	0.185913	b	0.737187	b	0.099887	0.996484	0.041765	0.450355	1.7918	3.84	0.0568
	unclassified	3.85285	a	0.045723	a	0.022177	a	0.016617	a	2.610942	0.045723	0.022177	0.016617	4.2583	2.15	0.1727

	Arthrobacter	1.638013	a	0.161367	a	0.164973	a	0.95578	a	0.763096	0.087613	0.058696	0.720168	1.7195	1.82	0.2215
	Pantoea	1.49955	a	0.477813	a	0.307773	a	0.554667	a	0.705271	0.369446	0.098923	0.392894	1.4576	1.44	0.301

Note: Different letters in same row indicate statistically significant differences at the 0.05 probability level according to Fisher’s least significant difference (LSD) test. WO: welsh onion; GA: Garlic; ON: onion.

Supplementary Table S3 Variance analysis of KEGG pathway in four *Allium* species

KEGG_Pathways	KEGG pathway	Total	Average								Error				SAS analysis		
Level_1	Level_2		CL		GA		ON		WO		CL	WO	GA	ON	LSD	F	P
Cellular Processes	Cell Communication	24	8	a	0	a	0	ab	0	a	5.291503	0	0	0	8.6283	2.29	0.1556
	Cell Growth and Death	247576	37350.33	a	13090	b	20254.67	ab	11830.33	b	9751.028	1062.788	7215.745	5365.046	21697	3.12	0.0882
	Cell Motility	878341	165853.7	a	47754	b	53236	b	25936.67	b	46440.33	7187.26	32787	17256.61	97579	4.42	0.0413
	Transport and Catabolism	115531	22464	a	5624	b	6912	b	3510.333	b	6445.007	761.0587	3601.154	2127.371	12590	5.05	0.0299
Environmental Information Processing	Membrane Transport	3944197	813097	a	162994.3	b	221384	b	117257	b	236358.4	16365.92	105141.4	61830.81	434518	5.98	0.0193
	Signal Transduction	675099	135395.3	a	30995	b	39242.67	b	19400	b	39034.37	4124.153	21483.5	11632.48	75388	5.33	0.026
	Signaling Molecules and Interaction	40750	10813	a	883.6667	b	1306	b	580.6667	b	3072.62	121.9021	715.9988	328.6144	5176.1	9.74	0.0048
Genetic Information Processing	Folding, Sorting and Degradation	869544	146674.3	a	42480	b	64285	ab	36408.67	b	40893.11	3917.739	24626.47	17260.04	83016	4	0.0519
	Replication and Repair	2719342	449350	a	132504.3	b	206388	ab	118205	b	124600.3	11384.09	75256.33	54335.34	254031	3.88	0.0556
	Transcription	747399	155008.3	a	28780	b	42476.33	b	22868.33	b	44663.21	2728.825	18411.27	11341.74	81037	6.3	0.0168
	Translation	1810473	277619.3	a	93980	b	146790.7	ab	85101	b	75836.96	7874.69	52092.23	38833.25	163343	3.14	0.0868
	Amino Acid Metabolism	3347228	664903.3	a	149086.7	b	199079	b	102673.7	b	186461.1	17109.06	94965.96	56917.95	354700	5.73	0.0216
	Biosynthesis of Other Secondary Metabolites	275906	59832.67	a	10736.67	b	14226	b	7173.333	b	17003.46	1300.601	7025.576	4040.596	30787	6.86	0.0133
	Carbohydrate Metabolism	3146600	639964.3	a	136205.3	b	181288.7	b	91408.33	b	183845.8	16248.94	90332.75	52108	345664	5.76	0.0213
	Energy Metabolism	2074489	356667.7	a	104804.3	b	149371.7	ab	80652.67	b	99766.1	11753.36	65727.22	41868.94	207313	3.92	0.0544
	Enzyme Families	579570	114435.3	a	24683.33	b	35640.67	b	18430.67	b	32646.85	2810.607	16899.48	9769.111	62193	5.48	0.0242

Metabolism	Glycan Biosynthesis and Metabolism	621560	123619	a	27856.33	b	36521.33	b	19190	b	36272.38	3407.973	17878.21	10678.58	68426	5.32	0.0261
	Lipid Metabolism	1273720	246481.7	a	60808.67	b	78034.33	b	39248.67	b	69571.84	7722.864	39237.74	23210.21	136212	5.16	0.0282
	Metabolism of Cofactors and Vitamins	1339142	255050	a	60921.33	b	85277	b	45132.33	b	71881.38	6815.524	39074.93	23833.46	139397	5.16	0.0283
	Metabolism of Other Amino Acids	576564	120269.3	a	24224.67	b	31694.67	b	15999.33	b	33808.62	2860.099	15863.49	9148.774	62869	6.35	0.0165
	Metabolism of Terpenoids and Polyketides	708831	135925.3	a	35234.67	b	43828.33	b	21288.67	b	38123.37	4914.375	23259	13363.85	76431	4.94	0.0316
	Nucleotide Metabolism	1085365	196778.7	a	49082.33	b	74497	b	41430.33	b	54972.99	4533.755	29440.99	19742.08	106914	4.86	0.0328
	Xenobiotics Biodegradation and Metabolism	1092061	236591.7	a	45587.67	b	55656.67	b	26184.33	b	65712.45	5904.119	29990.82	16667.34	121260	6.92	0.013

Note: Different letters in same row indicate statistically significant differences at the 0.05 probability level according to Fisher' s least significant difference (LSD) test. WO: welsh onion; GA: Garlic; ON: onion.

Supplementary Table S4 KEGG pathway identified in four *Allium* species by PICRUSt algorithm

KEGG_Pathways	KEGG pathway	KEGG pathway	Average								Error				SAS analysis		
Level_1	Level_2	Level 3	CL		GA		ON		WO		CL	WO	GA	ON	LSD	F	P
Cellular Processes	Cell Communication	Adherens junction	2.66667	a	0	a	0	a	0	a	1.76383	0	0	0	2.8761	2.29	0.1556
		Focal adhesion	2.66667	a	0	a	0	a	0	a	1.76383	0	0	0	2.8761	2.29	0.1556
		Tight junction	2.66667	a	0	a	0	a	0	a	1.76383	0	0	0	2.8761	2.29	0.1556
	Cell Growth and Death	Meiosis - yeast	2799	a	683	b	867.333	b	382	b	761.935	106.785	523.334	251.291	1571.6	5.17	0.0281
		p53 signaling	1499.67	a	523.667	b	950	ab	612.333	b	341.844	18.4059	220.935	226.184	759.88	3.6	0.0654
		Apoptosis	1433.33	a	525	b	952.333	ab	615	b	321.332	18.9297	221.101	226.581	736.18	3.32	0.0776
		Cell cycle - Caulobacter	31618	a	11358.3	b	17485	ab	10221	b	8331.48	939.839	6270.73	4662.02	18688	2.94	0.0988
		Cell cycle	0.33333	a	0	a	0	a	0	a	0.33333	0	0	0	0.5435	1	0.4411
	Cell Motility	Flagellar assembly	33209.3	a	8570.33	b	9214	b	4563.67	b	9128.93	1199.95	5820.39	3006.77	18426	5.32	0.0261
		Bacterial motility proteins	85268.7	a	24111	b	26123	b	12396.3	b	23977.6	3804.45	16880.1	8631.36	50227	4.52	0.039
		Bacterial chemotaxis	34676.3	a	10836.7	b	11550.3	b	5421.33	b	9923.9	1773.11	7566.99	3899.62	21515	3.88	0.0556
		Cytoskeleton proteins	12696.7	a	4236	b	6348.67	ab	3555.33	b	3416.9	419.135	2532.64	1724.32	7514.6	3.27	0.0802
		Regulation of actin cytoskeleton	2.66667	a	0	a	0	a	0	a	1.76383	0	0	0	2.8761	2.29	0.1556
	Transport and Catabolism	Lysosome	4608.67	a	308.667	b	377.667	b	178	b	1439.14	44.5172	214.945	102.666	2379.7	8.78	0.0065
		Endocytosis	25	a	1	b	4	b	0.33333	b	7.63763	1	2	0.33333	12.988	8.66	0.0068
		Phagosome	3	a	0	b	0	b	0	b	1.73205	0	0	0	2.8243	3	0.0951
		Peroxisome	17827.3	a	5314.33	b	6530.33	b	3332	b	4999.37	725.66	3387.91	2027.93	10455	4.13	0.481
Environmental Information Processing	Membrane Transport	Transporters	385976	a	66026.3	b	87468	b	44374.3	b	113076	6847.02	45449.6	24634.7	203043	6.68	0.0143
		ABC transporters	237947	a	45068	b	56688.3	b	28530	b	69768.8	5026.01	30605.6	16531.7	127384	6.29	0.0169
		Phosphotransferase system (PTS)	14988	a	1723.33	b	2590.67	b	979.333	b	5275.81	217.801	1851.23	751.341	9205.7	5.54	0.0236
		Secretion system	114992	a	31395	b	45543	b	26069.7	b	32102.2	2880.07	17745.4	12349.4	63284	4.5	0.0395
		Bacterial secretion system	59194	a	18781.7	b	29094	ab	17303.7	b	16340.3	1420.8	9754.27	7631.59	33513	3.58	0.0661
	Signal Transduction	MAPK signaling pathway - yeast	1852.33	a	104.667	b	210.667	b	95.3333	b	544.308	27.205	123.734	46.2037	914.38	9.39	0.0053
		Two-component	127127	a	29224.3	b	36316.7	b	17735	b	36715.4	3998.91	20414.4	10904.6	71070	5.32	0.0262
		Notch signaling pathway	18.6667	a	0	b	0	b	0.33333	b	8.19214	0	0	0.33333	13.369	5.12	0.0288
		Wnt signaling	18.6667	a	0	b	0	b	0.33333	b	8.19214	0	0	0.33333	13.369	5.12	0.0288
		Phosphatidylinositol signaling system	6357	a	1648.33	b	2678	b	1554	b	1755.15	127.422	927.578	678.404	3427.1	4.61	0.0373
		VEGF signaling pathway	8.66667	a	0	b	0	b	0	b	4.25572	0	0	0	6.9393	4.15	0.0478
		mTOR signaling pathway	0.33333	a	0	a	0	a	0	a	0.33333	0	0	0	0.5435	1	0.4411
		Calcium signaling pathway	13	a	17.6667	a	37.3333	a	15	a	4.04145	5.54777	27.5096	9.84886	48.942	0.56	0.6568
	Signaling Molecules and Interaction	Bacterial toxins	4624	a	408	b	585.333	b	260.333	b	1292.5	52.144	319.364	152.238	2186.7	9.88	0.0046
		Cellular antigens	4740.33	a	413.333	b	587	b	269.667	b	1343.87	54.3026	330.654	151.943	2271.9	9.63	0.0049
		Ion channels	1431	a	62.3333	b	132.667	b	50.6667	b	433.851	19.3678	66.1824	25.458	717.52	9.43	0.0053
		Neuroactive ligand-receptor interaction	5	a	0	b	0.66667	b	0	b	1.73205	0	0.66667	0	3.0262	6.74	0.014
		Glycan bindng	12	a	0	b	0.33333	b	0	b	5.2915	0	0.33333	0	8.6454	5.03	0.0301
		G protein-coupled receptors	0.66667	a	0	a	0	a	0	a	0.66667	0	0	0	1.0871	1	0.4411
	Folding, Sorting and Degradation	Ubiquitin system	1230	a	51.6667	b	100.333	b	36.3333	b	366.225	14.8474	41.434	19.6327	602.31	10.01	0.0044
		Sulfur relay system	20410.3	a	4273	b	5806.67	b	2943.67	b	5853.72	507.929	2834.13	1638.3	10968	5.83	0.0207
		Proteasome	2283.67	a	544.333	b	1018.33	b	619	b	610.838	36.8978	241.182	236.635	1139.8	5.31	0.0263
		RNA degradation	25453.7	a	7021	b	10716	b	6060	b	7069.84	645.014	4159.71	2863.28	14206	4.26	0.045
		Chaperones and folding catalysts	59347	a	16909	b	25375	b	14459.7	b	16672.4	1557.57	9746.73	6836.01	33502	4.08	0.0496
		Protein processing in endoplasmic reticulum	4494	a	1506	b	2017.67	ab	1093.67	b	1257.62	187.329	927.038	602.199	2747.3	3.28	0.0797
		Protein export	33455.7	a	12175	b	19251	ab	11196.3	b	9075.9	1000.09	6702.33	5068.38	20234	2.74	0.113
		Non-homologous end-joining	4047.33	a	163.333	b	293	b	147.333	b	1121.95	47.0791	100.271	67.8389	1841.7	11.61	0.0028
		Base excision	27579.7	a	5641.67	b	8982.33	b	5062.67	b	7663.96	472.278	3208.48	2273.3	14067	6.1	0.0183

Genetic Information Processing	Replication and Repair	Nucleotide excision repair	20180.7	a	5542.33	b	8577.67	b	4888	b	5533.48	477.586	3112.75	2261.82	11017	4.42	0.0411
		DNA repair and recombination proteins	149038	a	42130.7	b	65258	b	37297.3	b	41358.2	3652.07	23933.8	17216.3	83033	4.15	0.0478
		Chromosome	75300	a	21298	b	31552	b	17502.3	b	21172.5	2130.5	12989.7	8583.96	42994	4.07	0.0499
		Mismatch repair	40085.3	a	12969.7	b	20713	ab	12022	b	11041.7	1041.67	7200.9	5395.65	23288	3.32	0.0774
		DNA replication	32851.7	a	11037	b	17690.7	ab	10333.7	b	9020.36	866.536	6043.2	4593.62	19275	3.13	0.0875
		DNA replication proteins	55666.7	a	18597	b	29650	ab	17218.3	b	15414.6	1511.13	10343.3	7718.45	32874	3.12	0.0879
		Homologous recombination	44600.3	a	15124.7	b	23671.3	ab	13733.3	b	12301.8	1267.43	8363.61	6247.97	26390	3.1	0.0894
	Transcription	Basal transcription factors	267	a	12.3333	b	17.3333	b	7.66667	b	77.3111	7.42369	6.38575	5.36449	127.37	10.63	0.0036
		Transcription	93580.7	a	14324	b	19626.3	b	9851.67	b	27495.3	1509.73	10084.5	5421.73	48628	7.09	0.0122
		Transcription	53014.3	a	11377	b	17884	b	10103.7	b	14917.8	982.017	6698.04	4641.22	27764	5.65	0.0224
		RNA polymerase	8146.33	a	3066.67	b	4948.67	ab	2905.33	b	2187.03	240.132	1674.69	1290.96	4975.8	2.55	0.1289
	Translation	mRNA surveillance pathway	108.667	a	2.33333	b	6.33333	b	0	b	34.1435	1.85592	3.38296	0	56.028	9.5	0.0052
		Ribosome biogenesis in eukaryotes	3013	a	820	b	1317.67	b	757.333	b	823.77	67.55	445.028	329.383	1622.2	4.49	0.0397
		Ribosome Biogenesis	68844.3	a	19995	b	30104	ab	17028.3	b	19218.1	1833.02	11615.3	8086.19	39032	3.99	0.0523
		Translation factors	26163.7	a	9175.33	b	13904	ab	7979.33	b	7199.18	838.209	5155.84	3756.38	15744	2.96	0.0975
		Aminoacyl-tRNA biosynthesis	62192.3	a	22198.3	b	34876.3	ab	20326.3	b	16777.2	1811.46	12155.7	9222.61	37096	2.88	0.1028
		Ribosome	112969	a	40282.7	b	64922.7	ab	38271.7	b	30464.5	3080.99	21620.4	16872.6	67027	2.86	0.1044
		RNA	4328.67	a	1506.33	ab	1659.67	ab	738	b	1329.13	270.541	1132.23	567.884	3026.1	2.85	0.105
	Amino Acid Metabolism	Phenylalanine	25828	a	4431.67	b	5428.33	b	2723	b	7290.83	513.732	2801.48	1603.17	13028	7.41	0.0107
		Tyrosine metabolism	29493	a	4675	b	6216.67	b	3014	b	8445.24	531.474	3292.01	1752.57	15079	7.31	0.0112
		Histidine metabolism	33164	a	5695	b	6933.67	b	3162.33	b	9361	789.677	4168.87	2078.01	17098	7.17	0.0118
		Arginine and proline metabolism	75377	a	12762.7	b	17695.7	b	8862.33	b	21208.5	1412.41	8735.87	4830.39	38291	7.13	0.012
		Glycine, serine and threonine metabolism	60007	a	11234.3	b	15880.3	b	8515.33	b	16721.1	1067.73	6877.99	4292.13	30351	6.79	0.0137
		Alanine, aspartate and glutamate metabolism	51348.3	a	9755.67	b	12770	b	6345.33	b	14569.1	1173.4	6644.66	3663.34	26853	6.52	0.0153
		Cysteine and methionine metabolism	48702.3	a	9733	b	13231	b	6567.67	b	14056.1	1189.08	6806.33	3724.87	26251	5.94	0.0197
		Valine, leucine and isoleucine biosynthesis	43337.7	a	9291.33	b	13080.7	b	6989	b	12148.8	951.056	5800.22	3609.33	22780	5.9	0.02
		Phenylalanine, tyrosine and tryptophan	38931.3	a	8342.33	b	9951	b	4699	b	11196.7	1207.74	5854.31	3078.19	21296	5.84	0.0205
		Tryptophan metabolism	43740	a	10816.7	b	12751.7	b	6117.67	b	12172.2	1496.78	6873.78	3976.45	23824	5.51	0.0239
		Valine, leucine and isoleucine degradation	62478.7	a	17617.3	b	21255.7	b	10475.3	b	17142.3	2357.74	11028.7	6576.22	351356	4.74	0.0349
		Lysine degradation	35337	a	10025	b	11704.7	b	5625.67	b	9793.56	1426.09	6317.12	3714.01	20080	4.71	0.0356
		Lysine biosynthesis	37803.7	a	10741	b	15928.7	b	8871.67	b	10482.8	1027.69	6312.36	4320.93	21227	4.19	0.0468
		Amino acid related enzymes	79355.3	a	23965.7	b	36251	ab	20705.3	b	21928	2129.04	13587.5	9744.98	45099	3.82	0.0575
	Biosynthesis of Other Secondary Metabolites	Betalain biosynthesis	279.667	a	18	b	26.3333	b	16.6667	b	68.4211	4.72582	6.17342	7.33333	112.92	14.04	0.0015
		Isoflavonoid biosynthesis	29.3333	a	1	b	2	b	0.66667	b	7.51295	1	1.52753	0.33333	12.619	13.22	0.0018
		Caffeine metabolism	333.667	a	18	b	28.6667	b	14	b	85.5031	2.88675	10.8064	7.23418	141.1	13.14	0.0019
		Indole alkaloid biosynthesis	224.333	a	16.3333	b	19.3333	b	16	b	56.9044	4.25572	7.12585	7	94.462	12.78	0.002
		Stilbenoid, diarylheptanoid and gingerol	2314.33	a	126.667	b	310.333	b	88	b	620.366	35.4134	102.569	50.1431	1030.2	11.56	0.0028
		beta-Lactam resistance	2327.67	a	97.6667	b	200.667	b	77.3333	b	676.739	34.7003	111.919	38.5155	1121.7	10.28	0.0041
		Flavone and flavonol biosynthesis	291.333	a	10	b	15	b	11	b	91.0793	3.21455	7.50555	5.1316	149.34	9.3	0.0055
		Phenylpropanoid	6449.67	a	643.333	b	814.667	b	377.667	b	1913.57	91.0262	468.448	231.015	3237.8	8.68	0.0068
		Clavulanic acid biosynthesis	14	a	0.33333	b	1.33333	b	0	b	4.6188	0.33333	0.66667	0	7.6288	8.32	0.0077

		Penicillin and cephalosporin biosynthesis	4257.33	a	589	b	712.333	b	310.667	b	1226.67	89.6047	433.84	207.992	2153.5	8	0.0086
		Butirosin and neomycin biosynthesis	2689.33	a	340	b	454.333	b	200.333	b	790.704	46.6047	273.168	126.341	1381.6	7.8	0.0092
		Streptomycin biosynthesis	16601.3	a	3152.67	b	4092	b	2060.67	b	4683.72	403.279	2104.69	1188.51	8619.4	6.62	0.0147
		Tropane, piperidine and pyridine alkaloid biosynthesis	9060.67	a	1946	b	2579	b	1357.67	b	2553.34	215.818	1180.48	738.804	4755.5	6.04	0.0188
		Novobiocin biosynthesis	8413.67	a	1925.67	b	2524.67	b	1352.33	b	2389.43	212.863	1184.88	728.303	4521.5	5.58	0.0232
		Isoquinoline alkaloid biosynthesis	5097.67	a	1337	b	1863.67	b	1024.67	b	1428.33	135.015	780.947	521.604	2796	4.79	0.0339
		Flavonoid biosynthesis	1448.67	a	515	ab	581.667	ab	265.667	b	417.181	95.2593	379.027	201.397	988.27	2.89	0.102
	Carbohydrate Metabolism	Ascorbate and aldarate metabolism	12608.7	a	687	b	1166	b	489	b	3767.26	143.598	607.903	258.701	6241	9.57	0.005
		Pentose and glucuronate interconversions	27940.3	a	1939.33	b	3114	b	1352.67	b	8493.39	298.566	1779.99	743.199	14210	8.8	0.0065
		Starch and sucrose metabolism	34941.7	a	4085	b	5753	b	2461	b	10432.6	583.768	3569.95	1583.04	18189	7.7	0.0096
		Inositol phosphate metabolism	13370	a	1994.67	b	3247.67	b	1834.33	b	3813.99	181.851	1122.25	769.035	6609.5	7.48	0.0104
		Galactose metabolism	22854.7	a	3084.33	b	4059	b	1784	b	6870.79	421.199	2538.66	1164.07	12113	7.22	0.0115
		Pentose phosphate pathway	41893.7	a	7113	b	9069.33	b	4276.33	b	12253.2	949.203	5244.84	2672.74	22220	6.71	0.0142
		Fructose and mannose metabolism	35178	a	5527	b	7440	b	3410.67	b	10538.6	724.107	4276.59	2128.58	18904	6.65	0.0145
		Amino sugar and nucleotide sugar [illegible]	57682	a	10250	b	13900	b	6663	b	16881.1	1323.84	7594.51	3956.66	30941	6.34	0.0165
		Glycolysis / Gluconeogenesis	63237	a	12805.7	b	18559.3	b	9630.33	b	18107.9	1367.66	8462.36	5037.56	33685	5.89	0.0201
		Pyruvate metabolism	69488	a	15780	b	21420.3	b	11021.3	b	19804.7	1814.65	10279.8	6069.5	37822	5.44	0.0248
		Glyoxylate and dicarboxylate metabolism	50744	a	12014.3	b	15380	b	7939.67	b	14387.2	1424.62	7625.26	4505.2	27646	5.41	0.0251
		C5-Branched dibasic acid metabolism	19144.7	a	4604.33	b	5963	b	3041.33	b	5398.16	556.397	2974.09	1747.82	10485	5.3	0.0264
		Propanoate metabolism	64331.7	a	17686	b	21004	b	10168.7	b	17930	2462.78	11354.2	6586.12	36455	4.78	0.0341
		Citrate cycle (TCA [illegible]	54379	a	16042.3	b	24632.3	ab	14203.7	b	14975	1302.38	8718.91	6514.02	30261	4.02	0.0513
		Butanoate metabolism	72171	a	22592.3	b	26580.7	b	13132.3	b	20267.6	3172.6	14331.3	8463.84	43075	3.97	0.0528
	Energy Metabolism	Sulfur metabolism	18179	a	2592.67	b	3344.67	b	1500.67	b	5204.28	354.782	2057.25	965.849	9278	7.68	0.0097
		Nitrogen metabolism	46216	a	9073	b	11373.3	b	5531.67	b	13345.7	1165.32	6223.75	3348.93	24698	6.25	0.0172
		Methane metabolism	60613.7	a	16411.3	b	19125	b	8979.33	b	17439.7	2484.17	11345.2	6109.74	35589	4.55	0.0384
		Carbon fixation in photosynthetic organisms	31419.3	a	8608.33	b	12411.7	b	6819.67	b	8968.06	903.326	5272.31	3439.98	17927	4.24	0.0455
		Oxidative phosphorylation	91668.7	a	26555.7	b	44274	ab	26251.3	b	24698	1855.65	14114.4	11128.3	49900	4.06	0.0502
		Carbon fixation pathways in prokaryotes	70668	a	24323.3	b	31591	ab	16693	b	19831.2	3059.93	14931.4	9469.94	43609	3.23	0.0823
		Photosynthesi	18279	a	8205.67	a	12904	a	7125	a	4967.67	961.866	5467.6	3507.79	13427	1.53	0.2796
		Photosynthesis proteins	19572	a	8763.33	a	13790.3	a	7514.67	a	5330.71	1095.36	6080.44	3764.72	14654	1.49	0.2884
		Photosynthesis - antenna proteins	52	a	271	a	557.667	a	237.333	a	24.0069	82.9397	410.368	152.044	727.35	0.88	0.4921
	Enzyme Families	Cytochrome P450	66	a	2.33333	b	6	b	0.66667	b	14.5258	1.45297	1.73205	0.33333	23.977	18.45	0.0006
		Peptidases	89839.3	a	19291.3	b	28086.7	b	14836.3	b	25535.3	2089.11	12811.3	7675.76	48356	5.57	0.0233
		Protein kinases	24530	a	5389.67	b	7548	b	3593.67	b	7097.51	724.924	4090.91	2093.15	13838	5.17	0.0282
		Glycosphingolipid biosynthesis - lacto and neolacto series	24.6667	a	1.33333	b	0.66667	b	2.66667	b	6.56591	0.88192	0.66667	2.66667	111.695	10.44	0.0039
		Other glycan degradation	6516	a	136	b	299	b	129.667	b	2052.04	55.5068	152.67	63.4621	3358.1	9.45	0.0052
		Glycosphingolipid biosynthesis - ganglio [illegible]	1459	a	25	b	59.3333	b	28	b	462.667	10.9697	27.5096	13.8684	756.3	9.4	0.0053

		Glycosphingolipid biosynthesis - globo	2852.33	a	63	b	161.333	b	67.6667	b	907.029	24.5561	88.9688	32.3539	1487.6	9.13	0.0058
	Glycan Biosynthesis and Metabolism	various types of N-glycan	15	a	1.33333	b	1.33333	b	0	b	4.58258	0.33333	1.33333	0	7.8011	8.77	0.0066
		Glycosaminoglycan degradation	2592.33	a	267	b	309.667	b	142	b	816.751	38.0832	193.267	94.8701	1378.7	7.77	0.0093
		N-Glycan biosynthesis	1997.33	a	350.333	b	453.333	b	192.333	b	575.099	57.4524	276.821	130.314	1066.3	6.59	0.0148
		Lipopolysaccharide biosynthesis proteins	27649.3	a	5388.67	b	5758	b	2760.33	b	8388.07	810.965	3628.6	1906.27	15280	6.11	0.0182
		Glycosyltransferases	21946	a	4720	b	5809.33	b	2855.67	b	6372.06	663.042	3265.16	1792.36	12084	5.67	0.0222
		Lipopolysaccharide biosynthesis	20059.7	a	4493	b	4714.67	b	2278.33	b	6055.03	704	3017.55	1596.46	11392	5.5	0.0241
		Peptidoglycan	38507.3	a	12410.7	b	18954.7	ab	10733.3	b	10653.9	1146.53	7260.66	5097.69	22684	3.36	0.0759
		Arachidonic acid metabolism	4493	a	187	b	376	b	166.333	b	1292.91	58.2866	193.497	79.8798	2137.8	10.53	0.0038
Metabolism		Linoleic acid	3761.33	a	353.667	b	484.667	b	218.667	b	1047.25	48.1675	249.321	129.935	1769.9	9.9	0.0045
		Secondary bile acid biosynthesis	349.333	a	20.3333	b	41.6667	b	15.6667	b	108.898	5.89727	23.1828	8.76229	182.36	8.41	0.0074
		Steroid hormone biosynthesis	2630.67	a	313	b	378.333	b	167	b	790.394	45.2806	190.533	110.817	1340	8.19	0.008
		Sphingolipid metabolism	6191.67	a	576.667	b	715.667	b	322.333	b	1918.12	85.7483	432.744	214.588	3228.3	8.18	0.0081
		Biosynthesis of unsaturated	17774.7	a	2332.33	b	3339.67	b	1677.33	b	5097.59	250.156	1535.58	880.276	8808.4	8.11	0.0083
		Glycerolipid metabolism	20645	a	4067.67	b	5395.33	b	2526.33	b	5956.54	532.942	2928.57	1546.51	11147	6.05	0.0187
		Ether lipid metabolism	1074.33	a	249.667	b	259.333	b	122.333	b	306.167	39.8595	158.474	88.7888	584.12	5.94	0.0197
	Lipid Metabolism	Primary bile acid biosynthesis	1093.67	a	266.333	b	299.333	b	127.667	b	304.126	40.4159	172.86	98.1026	596.07	5.73	0.0216
		Fatty acid biosynthesis	35184.7	a	8155.33	b	11766.7	b	6330.33	b	9834.61	839.222	5157.16	3231.04	18908	5.35	0.0258
		Fatty acid elongation in mitochondria	36.3333	a	0	b	0.66667	b	0	b	15.6454	0	0.66667	0	25.534	5.32	0.0262
		alpha-Linolenic acid	3024.33	a	758.667	b	815.333	b	377.333	b	898.869	119.834	501.282	272.349	1746.9	5.04	0.03
		Lipid biosynthesis proteins	53023	a	14647	b	19065.3	b	9781.67	b	14819.7	1838.93	9300.31	5648.27	30129	4.52	0.0392
		Synthesis and degradation	11410.7	a	3508.33	b	3773	b	1713	b	3176.29	559.966	2332.14	1273.65	6814.1	4.24	0.0435
		Glycerophospholipid metabolism	32082	a	8694	b	12936.3	b	7205.67	b	9065.25	817.007	5212.27	3514.38	18037	4.32	0.0435
		Fatty acid metabolism	52570.3	a	16210.7	b	17887	b	8268	b	14652.8	2542.72	10558.5	5961.04	31288	4.21	0.0463
		Steroid biosynthesis	1136.67	a	468	ab	500	ab	229	b	333.651	86.3732	336.149	186.806	842.05	2.26	0.1586
		Vitamin B6 metabolism	10064	a	1323.33	b	1693.67	b	766.333	b	2916.44	172.455	1002.73	479.678	5097	7.99	0.0086
		Retinol metabolism	5547.67	a	924	b	1183	b	526.667	b	1568.76	124.557	680.615	342.898	2851.1	7.23	0.0115
		Folate biosynthesis	24583.3	a	4223.67	b	5924	b	3014	b	6943.19	484.061	2858.34	1613.79	12548	6.98	0.0127
		Nicotinate and nicotinamide metabolism	23270.7	a	4007.33	b	5238	b	2554.67	b	6643.07	487.273	2790.26	1490.1	12024	6.97	0.0128
		Thiamine metabolism	20301	a	3575.33	b	4812	b	2301.33	b	5891.71	469.013	2630.57	1364.93	10781	6.5	0.0154
		Biotin metabolism	8291.67	a	1730.67	b	2401.67	b	1269.33	b	2320.89	196.673	1108.95	671.02	4346.5	6.05	0.0187
		Riboflavin metabolism	15653.7	a	3355	b	4623.67	b	2460.33	b	4376.09	376.091	2093.91	1298.63	8211.9	5.97	0.0194
	Metabolism of Cofactors and Vitamins	Pantothenate and CoA biosynthesis	32613.7	a	6860	b	9133	b	4690.67	b	9278.83	801.436	4531.68	2616	17419	5.91	0.0199
		Lipoic acid metabolism	4341.33	a	1125	b	2120.67	ab	1286	b	1187.36	71.9236	588.947	495.227	2310.1	4.37	0.0423
		Porphyrin and chlorophyll	56428.7	a	16580	b	23201	b	12430.3	b	15696.1	1899.76	10554.8	6561.34	32792	3.96	0.0531
		One carbon pool by	29432.3	a	8875.33	b	12527.3	ab	6895	b	8164.82	936.397	5310.54	3522.37	16957	3.9	0.0549
		Ubiquinone and other terpenoid-quinone biosynthesis	24522	a	8341.67	b	12419	ab	6937.67	b	6921.31	845.397	4978.55	3393.14	15026	3.01	0.0947
		D-Arginine and D-ornithine metabolism	476	a	20.6667	b	43	b	23.3333	b	138.698	2.96273	24.0278	11.4066	230.33	10.03	0.0044

	Metabolism of Other Amino Acids	Cyanoamino acid metabolism	14205	a	1473.67	b	2425.33	b	1243.33	b	4108.61	169.564	1037.27	578.23	6979.2	8.59	0.007
		Phosphonate and phosphinate metabolism	4015.67	a	623.667	b	729.333	b	354.333	b	1169.57	74.2099	443.886	219.779	2074.6	7.4	0.0108
		Glutathione metabolism	27226.7	a	4679.67	b	6746.33	b	3683	b	7508.98	447.9	2927.86	1794.2	13483	7.3	0.0112
		Taurine and hypotaurine metabolism	7619	a	1243.67	b	1536	b	680.667	b	2184.59	168.272	934.409	456.346	3954.7	7.19	0.0117
		D-Alanine metabolism	4755.33	a	874	b	1039.33	b	479.667	b	1377.21	123.964	646.911	323.671	2544.6	6.52	0.0153
		beta-Alanine metabolism	32604.7	a	8281.33	b	9149.67	b	4154	b	9077.77	1231.2	5409.89	2990.45	18020	5.44	0.0247
		Selenocompound metabolism	22201.3	a	5146	b	7107.67	b	3720.33	b	6246.52	580.452	3367.64	2023.96	12070	5.34	0.0259
		D-Glutamine and D-glutamate metabolism	7165.67	a	1882	b	2918	b	1660.67	b	2007.37	166.689	1090.6	767.135	3938.9	4.51	0.0393
	Metabolism of Terpenoids and Polyketides	Biosynthesis of type II polyketide backbone	75.3333	a	0.33333	b	4	b	0.33333	b	14.0515	0.33333	2.64575	0.33333	23.328	26.65	0.0002
		Biosynthesis of type II polyketide products	16.6667	a	0	b	1.33333	b	0.33333	b	4.33333	0	0.88192	0.33333	7.2312	13.26	0.0018
		Sesquiterpenoid [illegible]	26	a	0.33333	b	3	b	0.33333	b	8.14453	0.33333	3	0.33333	14.174	8.21	0.008
		Biosynthesis of siderophore group nonribosomal [illegible]	4407	a	371.667	b	551	b	223.667	b	1406.72	51.2423	304.073	132.58	2358.2	7.78	0.0093
		Tetracycline biosynthesis	7400.33	a	1213.33	b	1548.33	b	698	b	2134.06	174.883	1006.04	462.237	3930.6	6.8	0.0136
		Polyketide sugar unit biosynthesis	8436.67	a	1675	b	1922	b	899	b	2382.67	264.727	1187.18	615.388	4476	6.49	0.0155
		Biosynthesis of ansamycins	4468.33	a	815	b	901	b	411.667	b	1366.48	121.006	572.402	291.185	2469.9	6.24	0.0172
		Limonene and pinene degradation	27563.3	a	6247	b	7221.67	b	3186.67	b	7572.31	914.123	4141.3	2274.1	14630	6.17	0.0178
		Carotenoid biosynthesis	2464.67	a	433.667	b	722.333	b	284.333	b	687.345	91.6958	485.656	192.825	1415.8	5.4	0.0252
		Biosynthesis of vancomycin group antibiotics	2892	a	782.333	b	850.667	b	391.667	b	813.071	135.327	540.904	285.996	1673.9	4.82	0.0335
		Terpenoid backbone biosynthesis	28714.3	a	7701.33	b	9938.33	b	4952	b	8061.03	1034.36	5092.74	2959.42	16366	4.62	0.0371
		Geraniol degradation	27560.3	a	9154.33	b	9648.33	b	4357.33	b	7670.19	1505.98	5882.18	3336.42	16854	3.9	0.055
		Prenyltransferases	19649	a	6060.67	b	9253	ab	5154	b	5423.66	598.991	3646.18	2498.17	11450	3.53	0.066
		Zeatin biosynthesis	2201.33	a	764	b	1227.33	ab	723.667	b	596.204	58.6884	409.159	318.85	1292.2	3.01	0.0946
		Biosynthesis of 12-, 14- and 16-membered macrolides	50	a	15.6667	b	36	b	5.66667	b	25.9422	5.36449	26.0832	5.66667	61.32	1.13	0.395
	Nucleotide Metabolism	Purine metabolism	114751	a	26701.7	b	39639.3	b	21664.3	b	32134.5	2595.16	16418	10601.4	61474	5.29	0.0265
		Pyrimidine metabolism	82027.7	a	22380.7	b	34857.7	b	19766	b	22839.3	1938.73	13030.4	9142.27	45504	4.3	0.0439
	Xenobiotics Biodegradation and Metabolism	Fluorobenzoate degradation	3925	a	187.333	b	330.667	b	142.667	b	1059.65	42.3491	130.052	70.2385	1746	11.99	0.0025
		Xylene degradation	3189.33	a	122.333	b	200	b	82.6667	b	900.048	44.2505	58.3467	39.4307	1473.9	11.43	0.0029
		Bisphenol degradation	8089.67	a	564	b	930	b	376.667	b	2182.85	109.162	346.68	204.244	3623.7	11.33	0.003
		Atrazine degradation	3774.33	a	219.667	b	370.667	b	183.333	b	1054.1	51.7472	149.122	84.9948	1743.5	10.48	0.0034
		Dioxin degradation	4423.67	a	190.333	b	308.333	b	145	b	1286.66	67.2343	119.892	68.942	2113	10.56	0.0037
		Chlorocyclohexane and chlorobenzene degradation	6963	a	634	b	913.667	b	413	b	1892.73	77.7775	453.717	223.393	3197.1	10.4	0.0039
		1,1,1-Trichloro-2,2-bis(4-chlorophenyl) ethane (DDT) degradation	276	a	5	b	11.6667	b	4.66667	b	86.1568	3.05505	3.1798	2.60342	140.73	9.71	0.0048
		Polycyclic aromatic hydrocarbon degradation	10040.3	a	1334	b	1764.33	b	772	b	2789.92	174.138	908.184	483.357	4857	8.7	0.0067
		Drug metabolism - other enzymes	12838.7	a	1464.33	b	1962.33	b	881	b	3700.69	176.409	1099.7	521.862	6358.9	8.6	0.0069
		Styrene degradation	6932	a	974.333	b	1150	b	553.667	b	1951.2	106.703	627.902	337.943	3391.9	8.49	0.0072
		Metabolism of xenobiotics by cytochrome [illegible]	11549	a	1723.33	b	2531.33	b	1382.33	b	3101.91	158.884	1062.71	659.557	5459.8	8.42	0.0074

		Drug metabolism - cytochrome P450	12701.7	a	1999	b	2865	b	1521.67	b	3410.79	194.634	1264.46	762.332	6068.7	8.16	0.0081
		Chloroalkane and chloroalkene degradation	17573	a	2663.33	b	3489.33	b	1510	b	4891.41	359.266	2005.91	980.174	8787	7.86	0.0091
		Ethylbenzene degradation	5772	a	1127.33	b	1421	b	636.333	b	1613.8	150.751	891.872	434.812	3098.8	6.26	0.0171
		Aminobenzoate [illegible]	30681.3	a	6836.33	b	7856	b	3510.33	b	8534.95	994.601	4623.5	2499.64	16424	6.11	0.0183
		Naphthalene degradation	17709.7	a	3830.67	b	4527.67	b	2029	b	4938.41	549.396	2702.12	1420.31	9509.1	6.1	0.0189
		Toluene degradation	13908.3	a	3287.33	b	5226	b	3053.67	b	3818.08	247.818	1691.94	1326.17	7156.2	5.44	0.0247
		Benzoate degradation	41036	a	10856.3	b	11830.3	b	5365.67	b	11483	1656.14	7123.99	3915.52	23099	5.16	0.0282
		Caprolactam degradation	18923.3	a	5565.67	b	5894.67	b	2665.67	b	5242	879.426	3485.62	2007.67	10869	4.74	0.0349
		Nitrotoluene degradation	6285.33	a	2003	b	2073.67	b	955	b	1835.53	328.087	1311.87	729.521	3903.2	3.89	0.0553

Note: Different letters in same row indicate statistically significant differences at the 0.05 probability level according to Fisher's least significant difference (LSD) test.
WO: welsh onion; GA: Garlic; ON: onion.

Supplementary Table S5 Correlation of the 20 bacterial genera (>1.0%) and the 23 KEGG categories

Code	Bacteria	KEGG pathway	correlation	P_value
1	g_unclassified;p_unclassified	Membrane_Transport	-0.741059258	0.0058214
2	g_unclassified;p_unclassified	Amino_Acid_Metabolism	-0.728751974	0.0071774
3	g_unclassified;p_unclassified	Carbohydrate_Metabolism	-0.731037408	0.0069093
4	g_unclassified;p_unclassified	Metabolism_of_Cofactors_and_Vitamins	-0.711985643	0.0093896
5	g_unclassified;p_unclassified	Lipid_Metabolism	-0.708113528	0.0099656
6	g_unclassified;p_unclassified	Xenobiotics_Biodegradation_and_Metabolism	-0.755629474	0.0044763
7	g_unclassified;p_unclassified	Nucleotide_Metabolism	-0.705329811	0.0103956
8	g_unclassified;p_unclassified	Transcription	-0.751916567	0.004794
9	g_unclassified;p_unclassified	Signal_Transduction	-0.713444827	0.0091791
10	g_unclassified;p_unclassified	Glycan_Biosynthesis_and_Metabolism	-0.720377588	0.0082269
11	g_unclassified;p_unclassified	Enzyme_Families	-0.723080825	0.0078763
12	g_unclassified;p_unclassified	Metabolism_of_Other_Amino_Acids	-0.745403718	0.0053922
13	g_unclassified;p_unclassified	Biosynthesis_of_Other_Secondary_Metabolites	-0.760912998	0.0040519
14	g_unclassified;p_unclassified	Transport_and_Catabolism	-0.704814906	0.0104767
15	g_unclassified;p_unclassified	Signaling_Molecules_and_Interaction	-0.813329792	0.0012927
16	g_Flavobacterium;p_Bacteroidetes	Membrane_Transport	0.824377825	0.0009719
17	g_Flavobacterium;p_Bacteroidetes	Amino_Acid_Metabolism	0.820585427	0.0010741
18	g_Flavobacterium;p_Bacteroidetes	Carbohydrate_Metabolism	0.821807339	0.0010403
19	g_Flavobacterium;p_Bacteroidetes	Metabolism_of_Cofactors_and_Vitamins	0.804002117	0.0016222
20	g_Flavobacterium;p_Bacteroidetes	Lipid_Metabolism	0.809072159	0.001436
21	g_Flavobacterium;p_Bacteroidetes	Xenobiotics_Biodegradation_and_Metabolism	0.847724932	0.0004964
22	g_Flavobacterium;p_Bacteroidetes	Nucleotide_Metabolism	0.788300285	0.0023181
23	g_Flavobacterium;p_Bacteroidetes	Cell_Motility	0.797443327	0.0018899
24	g_Flavobacterium;p_Bacteroidetes	Transcription	0.827371584	0.0008966
25	g_Flavobacterium;p_Bacteroidetes	Metabolism_of_Terpenoids_and_Polyketides	0.804553739	0.0016011
26	g_Flavobacterium;p_Bacteroidetes	Signal_Transduction	0.81648404	0.0011938
27	g_Flavobacterium;p_Bacteroidetes	Glycan_Biosynthesis_and_Metabolism	0.815689524	0.0012182
28	g_Flavobacterium;p_Bacteroidetes	Enzyme_Families	0.811056385	0.0013678
29	g_Flavobacterium;p_Bacteroidetes	Metabolism_of_Other_Amino_Acids	0.834838013	0.0007284
30	g_Flavobacterium;p_Bacteroidetes	Biosynthesis_of_Other_Secondary_Metabolites	0.844452033	0.0005489
31	g_Flavobacterium;p_Bacteroidetes	Transport_and_Catabolism	0.81090543	0.0013729
32	g_Flavobacterium;p_Bacteroidetes	Signaling_Molecules_and_Interaction	0.877111303	0.0001789
33	g_Flavobacterium;p_Bacteroidetes	Folding,_Sorting_and_Degradation	0.755420945	0.0044937
34	g_Flavobacterium;p_Bacteroidetes	Energy_Metabolism	0.758419366	0.0042482
35	g_Flavobacterium;p_Bacteroidetes	Replication_and_Repair	0.746447632	0.0052927
36	g_Flavobacterium;p_Bacteroidetes	Translation	0.701815431	0.0109581
37	g_unclassified;c_Gammaproteobacteria;p_Proteobacteria	Membrane_Transport	0.794824493	0.0020058
38	g_unclassified;c_Gammaproteobacteria;p_Proteobacteria	Amino_Acid_Metabolism	0.795213678	0.0019883
39	g_unclassified;c_Gammaproteobacteria;p_Proteobacteria	Carbohydrate_Metabolism	0.792640395	0.0021065
40	g_unclassified;c_Gammaproteobacteria;p_Proteobacteria	Metabolism_of_Cofactors_and_Vitamins	0.790234662	0.0022219
41	g_unclassified;c_Gammaproteobacteria;p_Proteobacteria	Lipid_Metabolism	0.786618113	0.0024044
42	g_unclassified;c_Gammaproteobacteria;p_Proteobacteria	Xenobiotics_Biodegradation_and_Metabolism	0.804660317	0.001597
43	g_unclassified;c_Gammaproteobacteria;p_Proteobacteria	Nucleotide_Metabolism	0.790501121	0.0022089
44	g_unclassified;c_Gammaproteobacteria;p_Proteobacteria	Cell_Motility	0.787707543	0.0023483
45	g_unclassified;c_Gammaproteobacteria;p_Proteobacteria	Transcription	0.799993631	0.0017821
46	g_unclassified;c_Gammaproteobacteria;p_Proteobacteria	Metabolism_of_Terpenoids_and_Polyketides	0.779941016	0.0027711
47	g_unclassified;c_Gammaproteobacteria;p_Proteobacteria	Signal_Transduction	0.798056616	0.0018636
48	g_unclassified;c_Gammaproteobacteria;p_Proteobacteria	Glycan_Biosynthesis_and_Metabolism	0.804412134	0.0016065
49	g_unclassified;c_Gammaproteobacteria;p_Proteobacteria	Enzyme_Families	0.790308153	0.0022183
50	g_unclassified;c_Gammaproteobacteria;p_Proteobacteria	Metabolism_of_Other_Amino_Acids	0.801338582	0.0017272
51	g_unclassified;c_Gammaproteobacteria;p_Proteobacteria	Biosynthesis_of_Other_Secondary_Metabolites	0.806682974	0.0015216
52	g_unclassified;c_Gammaproteobacteria;p_Proteobacteria	Transport_and_Catabolism	0.795081535	0.0019942
53	g_unclassified;c_Gammaproteobacteria;p_Proteobacteria	Signaling_Molecules_and_Interaction	0.8185276	0.001133
54	g_unclassified;c_Gammaproteobacteria;p_Proteobacteria	Folding,_Sorting_and_Degradation	0.77436386	0.0031088
55	g_unclassified;c_Gammaproteobacteria;p_Proteobacteria	Energy_Metabolism	0.764153295	0.0038072
56	g_unclassified;c_Gammaproteobacteria;p_Proteobacteria	Replication_and_Repair	0.775090802	0.0030631
57	g_unclassified;c_Gammaproteobacteria;p_Proteobacteria	Translation	0.74467287	0.0054627
58	g_unclassified;c_Gammaproteobacteria;p_Proteobacteria	Cell_Growth_and_Death	0.738119702	0.0061261
59	g_Pseudomonas;p_Proteobacteria	Signaling_Molecules_and_Interaction	0.707461083	0.0100652
60	g_Pedobacter;p_Bacteroidetes	Membrane_Transport	0.893348131	9.06E-05
61	g_Pedobacter;p_Bacteroidetes	Amino_Acid_Metabolism	0.880838021	0.0001544
62	g_Pedobacter;p_Bacteroidetes	Carbohydrate_Metabolism	0.886101822	0.0001243
63	g_Pedobacter;p_Bacteroidetes	Metabolism_of_Cofactors_and_Vitamins	0.866322712	0.0002674
64	g_Pedobacter;p_Bacteroidetes	Lipid_Metabolism	0.868836174	0.0002443
65	g_Pedobacter;p_Bacteroidetes	Xenobiotics_Biodegradation_and_Metabolism	0.901155636	6.28E-05
66	g_Pedobacter;p_Bacteroidetes	Nucleotide_Metabolism	0.853242227	0.0004168
67	g_Pedobacter;p_Bacteroidetes	Cell_Motility	0.837653832	0.0006717
68	g_Pedobacter;p_Bacteroidetes	Transcription	0.895905863	8.06E-05
69	g_Pedobacter;p_Bacteroidetes	Metabolism_of_Terpenoids_and_Polyketides	0.861753271	0.0003139
70	g_Pedobacter;p_Bacteroidetes	Signal_Transduction	0.872031173	0.0002172
71	g_Pedobacter;p_Bacteroidetes	Glycan_Biosynthesis_and_Metabolism	0.875674106	0.0001892
72	g_Pedobacter;p_Bacteroidetes	Enzyme_Families	0.876039584	0.0001865
73	g_Pedobacter;p_Bacteroidetes	Metabolism_of_Other_Amino_Acids	0.893382833	9.05E-05
74	g_Pedobacter;p_Bacteroidetes	Biosynthesis_of_Other_Secondary_Metabolites	0.903859236	5.49E-05
75	g_Pedobacter;p_Bacteroidetes	Transport_and_Catabolism	0.866721648	0.0002637
76	g_Pedobacter;p_Bacteroidetes	Signaling_Molecules_and_Interaction	0.931328485	1.07E-05
77	g_Pedobacter;p_Bacteroidetes	Folding,_Sorting_and_Degradation	0.822879417	0.0010113
78	g_Pedobacter;p_Bacteroidetes	Energy_Metabolism	0.8237778	0.0009875
79	g_Pedobacter;p_Bacteroidetes	Replication_and_Repair	0.813017182	0.0013028
80	g_Pedobacter;p_Bacteroidetes	Translation	0.773057007	0.0031922
81	g_Pedobacter;p_Bacteroidetes	Cell_Growth_and_Death	0.763963065	0.0038212
82	g_Pedobacter;p_Bacteroidetes	Cell_Communication	0.880046352	0.0001594
83	g_unclassified;unknow	Membrane_Transport	0.895693774	8.14E-05

84	g_unclassified;unknow	Amino_Acid_Metabolism	0.883622125	0.0001379
85	g_unclassified;unknow	Carbohydrate_Metabolism	0.88711917	0.0001191
86	g_unclassified;unknow	Metabolism_of_Cofactors_and_Vitamins	0.870590753	0.0002291
87	g_unclassified;unknow	Lipid_Metabolism	0.868443122	0.0002478
88	g_unclassified;unknow	Xenobiotics_Biodegradation_and_Metabolism	0.90206074	6.01E-05
89	g_unclassified;unknow	Nucleotide_Metabolism	0.862656316	0.0003042
90	g_unclassified;unknow	Cell_Motility	0.833240766	0.0007621
91	g_unclassified;unknow	Transcription	0.901831872	6.08E-05
92	g_unclassified;unknow	Metabolism_of_Terpenoids_and_Polyketides	0.859202515	0.0003424
93	g_unclassified;unknow	Signal_Transduction	0.871866784	0.0002185
94	g_unclassified;unknow	Glycan_Biosynthesis_and_Metabolism	0.876160937	0.0001857
95	g_unclassified;unknow	Enzyme_Families	0.880589133	0.000156
96	g_unclassified;unknow	Metabolism_of_Other_Amino_Acids	0.895995486	8.03E-05
97	g_unclassified;unknow	Biosynthesis_of_Other_Secondary_Metabolites	0.906367677	4.83E-05
98	g_unclassified;unknow	Transport_and_Catabolism	0.864494593	0.0002853
99	g_unclassified;unknow	Signaling_Molecules_and_Interaction	0.938474881	6.26E-06
100	g_unclassified;unknow	Folding,_Sorting_and_Degradation	0.832520823	0.0007777
101	g_unclassified;unknow	Energy_Metabolism	0.828215027	0.0008762
102	g_unclassified;unknow	Replication_and_Repair	0.825407933	0.0009454
103	g_unclassified;unknow	Translation	0.786608184	0.0024049
104	g_unclassified;unknow	Cell_Growth_and_Death	0.780018633	0.0027666
105	g_unclassified;unknow	Cell_Communication	0.834813484	0.0007289
106	g_unclassified;p_Chloroflexi	Membrane_Transport	0.860545874	0.0003271
107	g_unclassified;p_Chloroflexi	Amino_Acid_Metabolism	0.852106078	0.0004324
108	g_unclassified;p_Chloroflexi	Carbohydrate_Metabolism	0.853867236	0.0004085
109	g_unclassified;p_Chloroflexi	Metabolism_of_Cofactors_and_Vitamins	0.836395725	0.0006966
110	g_unclassified;p_Chloroflexi	Lipid_Metabolism	0.835638806	0.0007119
111	g_unclassified;p_Chloroflexi	Xenobiotics_Biodegradation_and_Metabolism	0.877830007	0.000174
112	g_unclassified;p_Chloroflexi	Nucleotide_Metabolism	0.826009543	0.0009303
113	g_unclassified;p_Chloroflexi	Cell_Motility	0.81374528	0.0012793
114	g_unclassified;p_Chloroflexi	Transcription	0.867890853	0.0002528
115	g_unclassified;p_Chloroflexi	Metabolism_of_Terpenoids_and_Polyketides	0.826549342	0.0009168
116	g_unclassified;p_Chloroflexi	Signal_Transduction	0.845451488	0.0005325
117	g_unclassified;p_Chloroflexi	Glycan_Biosynthesis_and_Metabolism	0.848477776	0.0004849
118	g_unclassified;p_Chloroflexi	Enzyme_Families	0.845740178	0.0005278
119	g_unclassified;p_Chloroflexi	Metabolism_of_Other_Amino_Acids	0.86761426	0.0002554
120	g_unclassified;p_Chloroflexi	Biosynthesis_of_Other_Secondary_Metabolites	0.879516943	0.0001628
121	g_unclassified;p_Chloroflexi	Transport_and_Catabolism	0.836544987	0.0006936
122	g_unclassified;p_Chloroflexi	Signaling_Molecules_and_Interaction	0.92208105	1.98E-05
123	g_unclassified;p_Chloroflexi	Folding,_Sorting_and_Degradation	0.790689677	0.0021997
124	g_unclassified;p_Chloroflexi	Energy_Metabolism	0.786232915	0.0024245
125	g_unclassified;p_Chloroflexi	Replication_and_Repair	0.784112066	0.0025374
126	g_unclassified;p_Chloroflexi	Translation	0.736412193	0.0063085
127	g_unclassified;p_Chloroflexi	Cell_Growth_and_Death	0.729780406	0.0070558
128	g_Rhodococcus;p_Actinobacteria	Membrane_Transport	0.71316346	0.0092195
129	g_Rhodococcus;p_Actinobacteria	Amino_Acid_Metabolism	0.707710647	0.010027
130	g_Rhodococcus;p_Actinobacteria	Carbohydrate_Metabolism	0.707453792	0.0100663
131	g_Rhodococcus;p_Actinobacteria	Xenobiotics_Biodegradation_and_Metabolism	0.736446305	0.0063048
132	g_Rhodococcus;p_Actinobacteria	Transcription	0.72232478	0.0079732
133	g_Rhodococcus;p_Actinobacteria	Metabolism_of_Other_Amino_Acids	0.723833387	0.0077808
134	g_Rhodococcus;p_Actinobacteria	Biosynthesis_of_Other_Secondary_Metabolites	0.735653475	0.0063908
135	g_Rhodococcus;p_Actinobacteria	Signaling_Molecules_and_Interaction	0.78383533	0.0025524
136	g_Enterobacter;p_Proteobacteria	Membrane_Transport	0.829034168	0.0008568
137	g_Enterobacter;p_Proteobacteria	Amino_Acid_Metabolism	0.80995309	0.0014054
138	g_Enterobacter;p_Proteobacteria	Carbohydrate_Metabolism	0.817734828	0.0011563
139	g_Enterobacter;p_Proteobacteria	Metabolism_of_Cofactors_and_Vitamins	0.794931937	0.002001
140	g_Enterobacter;p_Proteobacteria	Lipid_Metabolism	0.7943284	0.0020284
141	g_Enterobacter;p_Proteobacteria	Xenobiotics_Biodegradation_and_Metabolism	0.82997256	0.0008349
142	g_Enterobacter;p_Proteobacteria	Nucleotide_Metabolism	0.783857905	0.0025512
143	g_Enterobacter;p_Proteobacteria	Cell_Motility	0.74549347	0.0053836
144	g_Enterobacter;p_Proteobacteria	Transcription	0.833941645	0.0007472
145	g_Enterobacter;p_Proteobacteria	Metabolism_of_Terpenoids_and_Polyketides	0.784595949	0.0025113
146	g_Enterobacter;p_Proteobacteria	Signal_Transduction	0.795231734	0.0019874
147	g_Enterobacter;p_Proteobacteria	Glycan_Biosynthesis_and_Metabolism	0.799827661	0.001789
148	g_Enterobacter;p_Proteobacteria	Enzyme_Families	0.808922811	0.0014412
149	g_Enterobacter;p_Proteobacteria	Metabolism_of_Other_Amino_Acids	0.823711873	0.0009893
150	g_Enterobacter;p_Proteobacteria	Biosynthesis_of_Other_Secondary_Metabolites	0.836220506	0.0007001
151	g_Enterobacter;p_Proteobacteria	Transport_and_Catabolism	0.786511876	0.0024099
152	g_Enterobacter;p_Proteobacteria	Signaling_Molecules_and_Interaction	0.873115333	0.0002085
153	g_Enterobacter;p_Proteobacteria	Folding,_Sorting_and_Degradation	0.75072643	0.0048994
154	g_Enterobacter;p_Proteobacteria	Energy_Metabolism	0.749394433	0.0050194
155	g_Enterobacter;p_Proteobacteria	Replication_and_Repair	0.740766245	0.0058512
156	g_Enterobacter;p_Proteobacteria	Translation	0.701222663	0.0110551
157	g_Enterobacter;p_Proteobacteria	Cell_Communication	0.921755376	2.02E-05
158	g_Achromobacter;p_Proteobacteria	Membrane_Transport	0.818798002	0.0011251
159	g_Achromobacter;p_Proteobacteria	Amino_Acid_Metabolism	0.807248366	0.001501
160	g_Achromobacter;p_Proteobacteria	Carbohydrate_Metabolism	0.811795362	0.001343
161	g_Achromobacter;p_Proteobacteria	Metabolism_of_Cofactors_and_Vitamins	0.789228877	0.0022716
162	g_Achromobacter;p_Proteobacteria	Lipid_Metabolism	0.792322364	0.0021215
163	g_Achromobacter;p_Proteobacteria	Xenobiotics_Biodegradation_and_Metabolism	0.834600699	0.0007333
164	g_Achromobacter;p_Proteobacteria	Nucleotide_Metabolism	0.773934264	0.003136
165	g_Achromobacter;p_Proteobacteria	Cell_Motility	0.7599827	0.0041243
166	g_Achromobacter;p_Proteobacteria	Transcription	0.823409812	0.0009972
167	g_Achromobacter;p_Proteobacteria	Metabolism_of_Terpenoids_and_Polyketides	0.785083583	0.0024852
168	g_Achromobacter;p_Proteobacteria	Signal_Transduction	0.796259989	0.0019416
169	g_Achromobacter;p_Proteobacteria	Glycan_Biosynthesis_and_Metabolism	0.797253479	0.0018982
170	g_Achromobacter;p_Proteobacteria	Enzyme_Families	0.800803736	0.0017489
171	g_Achromobacter;p_Proteobacteria	Metabolism_of_Other_Amino_Acids	0.823040967	0.001007

172	g_Achromobacter;p_Proteobacteria	Biosynthesis_of_Other_Secondary_Metabolites	0.835162831	0.0007217
173	g_Achromobacter;p_Proteobacteria	Transport_and_Catabolism	0.788435268	0.0023113
174	g_Achromobacter;p_Proteobacteria	Signaling_Molecules_and_Interaction	0.876091511	0.0001862
175	g_Achromobacter;p_Proteobacteria	Folding,_Sorting_and_Degradation	0.737533265	0.0061883
176	g_Achromobacter;p_Proteobacteria	Energy_Metabolism	0.739285004	0.0060039
177	g_Achromobacter;p_Proteobacteria	Replication_and_Repair	0.727032369	0.0073842
178	g_Achromobacter;p_Proteobacteria	Cell_Communication	0.77771922	0.0029021
179	g_Devosia;p_Proteobacteria	Membrane_Transport	0.703494142	0.0106867
180	g_Devosia;p_Proteobacteria	Carbohydrate_Metabolism	0.70257895	0.010834
181	g_Devosia;p_Proteobacteria	Xenobiotics_Biodegradation_and_Metabolism	0.72894399	0.0071546
182	g_Devosia;p_Proteobacteria	Transcription	0.703147688	0.0107423
183	g_Devosia;p_Proteobacteria	Metabolism_of_Other_Amino_Acids	0.714155448	0.0090779
184	g_Devosia;p_Proteobacteria	Biosynthesis_of_Other_Secondary_Metabolites	0.722274199	0.0079797
185	g_Devosia;p_Proteobacteria	Signaling_Molecules_and_Interaction	0.752424701	0.0047496
186	g_Sphingobacterium;p_Bacteroidetes	Signaling_Molecules_and_Interaction	0.73277786	0.0067102
187	g_Delftia;p_Proteobacteria	Membrane_Transport	0.71543512	0.0088978
188	g_Delftia;p_Proteobacteria	Carbohydrate_Metabolism	0.70286061	0.0107885
189	g_Delftia;p_Proteobacteria	Xenobiotics_Biodegradation_and_Metabolism	0.716729414	0.0087183
190	g_Delftia;p_Proteobacteria	Transcription	0.722306349	0.0079756
191	g_Delftia;p_Proteobacteria	Metabolism_of_Other_Amino_Acids	0.709336095	0.009781
192	g_Delftia;p_Proteobacteria	Biosynthesis_of_Other_Secondary_Metabolites	0.723835317	0.0077805
193	g_Delftia;p_Proteobacteria	Signaling_Molecules_and_Interaction	0.769029503	0.0034602
194	g_Delftia;p_Proteobacteria	Cell_Communication	0.862513936	0.0003057
